# Augmented and Virtual Reality in Dental Medicine Updated: A Systematic Review and Meta‐Analysis

**DOI:** 10.1111/jre.70115

**Published:** 2026-04-25

**Authors:** Sofya Sadilina, Syed Majeed Quadri, Ronald E. Jung, Eugenia Settecase, Marc Balmer, Tim Joda

**Affiliations:** ^1^ Clinic of Reconstructive Dentistry, Center for Dental Medicine University of Zurich Zurich Switzerland; ^2^ Department of Prosthodontics and Oral Rehabilitation University of Coimbra Coimbra Portugal; ^3^ Department of Reconstructive Dentistry, University Center for Dental Medicine Basel University of Basel Basel Switzerland

**Keywords:** augmented reality, computer simulation, dentistry, digital technology, mixed reality, oral medicine, virtual reality

## Abstract

**Aim:**

This updated systematic review aimed to synthesize and evaluate the available evidence on the use of augmented reality (AR), mixed reality (MR), and virtual reality (VR) technologies in dental medicine and education. Both qualitative synthesis and quantitative analysis through pairwise meta‐analysis, where applicable, were performed to assess their effectiveness and potential applications in clinical and educational settings.

**Methods:**

A systematic electronic search was conducted, incorporating an updated search strategy from the original publication to reflect updated terminology, for articles published between January 1, 2019, and July 14, 2025. Articles reporting the use of AR, MR, or VR in clinical treatment or dental education were eligible for inclusion. Data on study characteristics, target population, dental discipline, application concept, type of reality, primary and secondary outcomes were extracted. Pairwise frequentist meta‐analysis was conducted on randomized controlled trials (RCTs) assessing VR and objective stress‐related clinical parameters such as heart rate. Study quality was appraised using the corresponding scale according to the study design.

**Results:**

A total of 110 studies, reporting the use of AR, MR or VR, encompassing 8573 participants, were included. VR was the most frequently investigated technology (*n* = 94), followed by AR (*n* = 11) and MR (*n* = 2). Most studies evaluated multiple applications: clinical or patient‐centered applications were evaluated in 71 trials, operator‐ or clinician‐orientated applications in 33, and educational applications in 132 studies. Among primary outcomes, patient‐reported (*n* = 30) and educational outcomes (*n* = 57) predominated, whereas clinical, clinician‐, and operator‐related outcomes were less frequently assessed. Results from the pairwise meta‐analyses of RCTs showed that VR significantly reduced anxiety levels in children patients when compared to tell‐show‐do techniques (SMD = −1.32, 95% CI −1.93 to −0.72); meanwhile, there was a borderline significant reduction in heart rate levels of children patients under the VR technique compared to tell‐show‐do technique (MD = −3.26, 95% CI −6.51 to −0.00). On the other hand, no significant differences were found in terms of physiological and reported outcomes in adult patients.

**Conclusion:**

AR, MR and VR are increasingly used in dental medicine and education, mainly in undergraduate and postgraduate training. Current evidence supports the effectiveness of immersive technologies, particularly VR, in enhancing learning outcomes for students and improving patient experience, especially in anxiety and pain management. However, conclusions are limited by heterogeneity and a small number of high‐quality studies, with overall low to moderate certainty of evidence.

**Trial Registration:**

PROSPERO number: CDR42023403360 and date of registration 10/03/2023. Start date of systematic review 13/03/2023.

## Graphical abstract

Immersive technologies, as augmented reality, mixed reality, and virtual reality, are increasingly used in dental medicine and education, showing benefits for learning outcomes and anxiety reduction, while clinical evidence remains heterogeneous and limited.

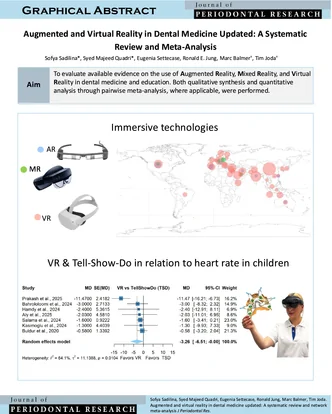

## 1 Introduction

The rapid development of digital technologies over the past decades, along with their stable integration in the field of dental medicine, has led to significant improvements in clinical workflows and educational methodologies [1, 2, 3, 4]. These technologies have been opening new paths for clinical practice, patient care and learning experiences for students and dental professionals and providers [5]. In dental medicine, augmented reality (AR), mixed reality (MR) and virtual reality (VR) technologies are mostly applied across three main outcome domains: clinical and patient‐orientated, clinician‐/operator‐centered and educational or training applications. Similar domains were reported in a recently published scoping review, which explored clinician‐ and patient‐reported outcomes of AR and MR within the narrow clinical field of implant dentistry [6].

AR and VR technologies are a part of the reality‐virtuality continuum, combining different amounts of true and artificial environments, both spatially and cognitively. They serve as the technological basis for integrating different imaging modalities, creating virtual patients, and non‐invasive simulations. A growing body of evidence demonstrates a rising interest in the implementation of AR and VR in clinical and academic settings [7, 8].

AR involves superimposing virtual content onto the real world. While various display technologies can enable AR, it is most realized using optical see‐through head‐mounted devices. In dentistry, one of its primary advantages is the real‐time overlay of radiological data directly onto the surgical field [9]. This approach, for example, eliminates the need for frequent switches between navigation data on the monitor and the operation field [10]. This improves ergonomics and minimizes hand‐eye coordination issues [11, 12]. VR has shown benefits in facilitating patient communication, particularly in the explanation of comprehensive treatment planning. Three‐dimensional (3D) visualization can help patients better understand complex staged treatment [13]. Moreover, VR distraction, as a behavior guidance technique, can reduce pain and anxiety associated with various dental treatments or anesthesia, especially in pediatric dentistry, similar to traditional approaches such as tell‐show‐do (TSD) in which the dentist explains the procedure, demonstrates it using instruments or models, and then performs the treatment—and audiovisual distraction, where children watch cartoons or videos during dental treatment on television (TV) or on tablet applications (apps) [14, 15]. Additionally, VR can be useful in early oral health education, promoting dental care learning in children [16].

In the education, VR and MR offer interactive and immersive learning experiences that are particularly valuable in interdisciplinary medical fields such as anatomy and pathology [17]. These technologies allow students to repeatedly view educational content at their own pace, enhancing interest and self‐directed learning. VR places students inside an entirely computer‐generated environment, whereby handling three‐dimensional holograms—rotating, enlarging, and examining anatomical structures—users receive a clearer spatial understanding of complex anatomical regions [18]. Moreover, haptic VR simulators can facilitate learning procedures with force feedback, such as dental cavity preparation or implant placement [19]. MR further expands these capabilities by overlaying digital information onto real‐world environments, allowing for simulated surgical training in a safe and controlled environment. The integration of VR and MR into education has accelerated since the COVID‐19 pandemic [20, 21].

Our systematic review with pairwise meta‐analysis is an update of the systematic review (SR) conducted by Joda and colleagues [22], which evaluated the scientific progress of AR and VR in dentistry. The original review identified a limited number of studies investigating AR/VR technologies in the field of dental medicine and an increasing interest in the utilization of current technologies in dental education. Additionally, it emphasized the need for future research to establish technological standards for generating high data quality and to develop clinically validated AR/VR applications for daily use. Since the publication of the original review, the field of immersive technologies has experienced rapid growth, with new studies published not only about AR and VR, but also MR, as a relatively new defined area. Moreover, several non‐systematic reviews aimed to map the available evidence were published in different fields of dentistry, analyzing one or several immersive technologies [6, 15]. Some articles identified in the previously published scoping reviews can also be eligible for inclusion in the present SR. In contrast to them, the present updated review therefore aims to systemically synthesize newly available evidence on the use of AR, MR, and VR technologies, with particular focus on their clinical and educational implementation as well as on their effectiveness based on clearly defined primary and secondary outcomes. That fact makes it relevant to synthesize the newly available evidence to increase the likelihood of achieving a more comprehensive and accurate conclusion. Conducting a meta‐analysis allows for quantitatively pooling the data, enhances statistical power, and provides generalizable conclusions regarding the effectiveness and applicability of AR and VR technologies in dental medicine.

## 2 Methods

### 2.1 Protocol and Registration

This review update followed the methodological outlines of the original study, with minor modifications to reflect recent technological developments in the field. The current systematic review is reported according to the guidelines of the Preferred Reporting Items of Systematic Reviews and Meta‐Analyses 2020 statement [23], and the Cochrane collaboration guidelines [24]. Before the start of the review process, a detailed protocol has been designed and registered on PROSPERO, an international prospective register of systematic reviews (CRD42023403360; March 10, 2023).

### 2.2 Terminology

Due to the heterogeneity in the terminology reported in the literature, the following definitions, based on the Glossary of Computer‐assisted Implant Surgery and related terms [25] and the US Food and Drug Administration [26] were adopted to classify virtual simulation technologies applied in implant dentistry and prosthodontics:

Augmented reality (AR) are technologies which superimpose digital content onto the real world, typically through a screen, such as a smartphone, tablet, head‐up display or AR glasses. AR adds layers of information on top of the real‐world view but does not necessarily integrate or interact with it.

Mixed reality (MR) are technologies which seamlessly integrate digital objects into the real world. MR combines both real and virtual elements in ways that they can interact with each other, creating a more immersive experience than AR. MR enables a higher level of interaction and environmental awareness.

Virtual reality (VR) are technologies which create a 3D immersive, digital environment, isolating the user from the real world, with or without interaction. The digital environment can typically be accessed through a VR headset.

### 2.3 Classification of AR, MR and VR Application Protocols

Given the wide range of applications of AR, MR, and VR technologies in dentinal medicine, intervention protocols were classified according to their application context. Accordingly, included studies were categorized into clinical (patient‐centered) applications, operator‐ or clinician‐centered applications, and educational or training applications.

Clinical (patient‐centered) applications aimed to improve the patient experience during dental procedures, primarily through distraction or anxiety reduction. Target populations included adult or pediatric patients. Outcomes comprised subjective patient‐reported measures (e.g., anxiety, pain, comfort) and objective physiological parameters (e.g., heart rate, blood pressure).

Operator‐ or clinician‐centered applications focused on supporting clinical procedures by enhancing visualization, procedural accuracy, or navigation. Outcomes included technical performance indicators, such as accuracy, precision, procedural time, error rates, and other technical performance indicators.

Educational or training applications were designed to facilitate learning and skill acquisition in undergraduate, postgraduate, or continuing dental education. Outcomes included cognitive outcomes, as knowledge acquisition, skill performance, simulation accuracy, task completion time; and learner‐related measures such as usability, acceptance, and satisfaction.

### 2.4 Research Question

The updated systematic review follows the methodological approach of the original publication and is formulated using a modified PICOs framework, omitting the comparison element due to the exploratory nature of the review. The focused question was:

“What are the applications and effects of augmented, mixed or virtual reality technologies in dental medicine, research and education?”

*Population (P)*: Patients of any age requiring any dental treatment; undergraduate and postgraduate dental students; dental professionals; and preclinical or experimental models, depending on the application domain;

*Intervention (I)*: The use of augmented, mixed or virtual reality technologies;

*Comparison (C)*: Omitted;

*Outcomes (O)*: Application‐specific outcomes:

- Any type of clinical outcomes, such as accuracy, treatment success rates, complication rates, and other standardized outcomes.
- Any type of clinician‐reported outcomes, as perceived usability, acceptance, overall satisfaction or other outcomes evaluated with questionnaires, VAS, or any other validated methods.
- Any type of patient‐reported outcomes, including pain perception, satisfaction, comfort, anxiety, or other outcomes evaluated with questionnaires, VAS, or any other standardized instruments.
- Any type of operator‐related outcomes, such as overall hand‐eye coordination, posture ergonomics, precision of movements, and other outcomes.
- Any type of educational and training outcomes, including standardized indices assessing students' performance, learning curves, tasks completion, failure rate or other standardized outcomes.

*Study* (S): Clinical trials, as randomized controlled trials (RCTs) of parallel, split‐mouth or crossover design, non‐randomized controlled trials (non‐RCTs), prospective and retrospective cohort studies, cross‐sectional and observational studies, case–control studies, case series, feasibility and proof‐of concept studies; preclinical studies, as in vitro, in vivo, ex vivo and in silico studies and educational, as true experimental, quasi‐experimental and pre‐experimental studies.

Study eligibility criteria were assessed based on the PIO framework and in detail reported in Table 1. All decisions regarding the eligibility criteria were made by the group of review authors, including dentists with professional experience in various fields of dental medicine.

**TABLE 1:** Summary of review search strategy and eligibility criteria.

Database search	
Electronic (Databases and Registers)	MEDLINE via PubMed Embase Cochrane Central Register of Controlled Trials (CENTRAL)
Manual (Journals)	American Journal of Orthodontics & Dentofacial Orthopedics British Journal of Oral & Maxillofacial Surgery Clinical Implant Dentistry & Related Research Clinical Oral Implants Research Clinical Oral Investigations Dentomaxillofacial Radiology European Journal of Oral Implantology European Journal of Orthodontics Facial & Plastic Surgery Clinics of North America Implant Dentistry International Journal of Oral & Maxillofacial Implants International Journal of Oral & Maxillofacial Surgery Journal of Clinical Periodontology Journal of Clinical Prosthodontics Journal of Cranio‐Maxillofacial Surgery Journal of Computerized Dentistry Journal of Dental Research Journal of Oral & Maxillofacial Surgery Journal of Oral Implantology Journal of Periodontology Journal of Periodontal & Implant Science Journal of Prosthetic Dentistry Journal of Prosthodontics Journal of Prosthodontic Research Oral Surgery Oral Medicine Oral Pathology & Radiology Orthodontics & Craniofacial Research
Other sources	Forward and backward reference checking from reviewed papers, relevant systematic reviews on the topic
Key terms	*Population/Topic*: existing scientific literature; *Intervention/Method*: (virtual reality) OR (augmented reality) OR (mixed reality); *Outcome/Interest*: (dentistry) OR (dental medicine) OR (prosthodontics) OR (endodontology) OR (periodontology) OR (implantology) OR (orthodontics) OR (oral surgery) OR (maxillofacial surgery)
Limits	
Dates	January 1st, 2019—July 14th, 2025
Filter	Humans
Language	English
Location	International
Article type	Academic
Eligibility criteria	
Types of studies	All types of clinical studies: randomized controlled trials, prospective/retrospective cohort studies, case series and case report(s); All types of educational studies: quantitative, qualitative, and mixed‐method studies in association of the dental field of interest
Inclusion criteria	Examination of at least one patient, dentist or dental student related to the topic of AR and/or MR and/or VR in the context of dentistry or dental education; Detailed information available on the AR‐, and MR‐ and VR‐technology used and its association of the dental field of interest
Exclusion criteria	Inadequate study type (e.g., abstracts, reviews, book chapters etc.); Insufficient information on the used AR/MR/VR‐technology; Studies not related to dentistry (e.g., those not directly related to teeth and focusing on maxillofacial surgery, otorhinolaryngology or other medical fields) or dental education; Multiple publications on the same population; Trial registered, not yet published; Inclusion of less than 15 participants per study arm or sample^a^; Studies not published in English

Abbreviations: AR, augmented reality; MR, mixed reality; VR, virtual reality.

^a^ The minimum sample size threshold was applied to limit the influence of severely underpowered studies; however, it may have led to the exclusion of early proof‐of‐concept studies.

Only articles published in English were considered.

### 2.5 Information Source and Search

The search strategy was adapted from the original SR [22] to reflect updated terminology. An electronic search was conducted in MEDLINE (via PubMed), EMBASE and Cochrane Central Register of Controlled Trials (CENTRAL) covering the period from January 1st, 2019 (the final search date of the original review) to July 14th, 2025. No further searches were performed after the last update. The electronic search was performed independently and in duplicate by two review authors (SS, QS) to verify the number of identified records. A combination of “Medical Subject Headings” and free‐text terms was used, with the filter “humans” applied. The search was restricted to publications in English in peer‐reviewed journals. The complete database specific search strategy was provided in Table 2. To maintain consistency with the original publication, a manual search of 26 relevant journals was performed (SS) to identify records not available electronically or lacking an electronic abstract (Table 1). Additionally, both forward and backward reference checking of the included manuscripts and relevant systematic reviews [27, 28, 29, 30] on the topic was performed to maximize the pool of relevant studies (SS).

**TABLE 2:** Search strategies.

Databases	
Medline (via PUBMED)	#1 (((virtual reality) OR (augmented reality) OR (mixed reality)) AND ((dentistry) OR (dental medicine) OR (prosthodontics) OR (endodontology) OR (periodontology) OR (implantology) OR (orthodontics) OR (oral surgery) OR (maxillofacial surgery))) #2 (2019:2025[pdat]) #3 #1 AND #2 #4 #3 AND (humans[Filter]) AND (English[Filter])
Embase	#1 (((“virtual reality”) OR (“augmented reality”) OR (“mixed reality”)) AND ((dentistry) OR (“dental medicine”) OR (prosthodontics) OR (endodontology) OR (periodontology) OR (implantology) OR (orthodontics) OR (“oral surgery”) OR (“maxillofacial surgery”))) #2 (2019:py OR 2020:py OR 2021:py OR 2022:py OR 2023:py OR 2024:py OR 2025:py) #3 #1 AND #2 #4 #3 AND [english]/lim AND [humans]/lim
Cochrane Central Register of Controlled Trials (CENTRAL)	#1 MeSH descriptor: [Virtual Reality] explode all trees #2 MeSH descriptor: [Augmented Reality] explode all trees #3 (virtual reality) OR (augmented reality) OR (mixed reality) #4 #1 OR #2 OR #3 #5 MeSH descriptor: [Dentistry] explode all trees #6 ((dentistry) OR (“dental medicine”) OR (prosthodontics) OR (endodontology) OR (periodontology) OR (implantology) OR (orthodontics) OR (“oral surgery”) OR (“maxillofacial surgery”)) #7 #5 OR #6 #8 #4 AND #7 Limits: Jan 2019 to Apr 2025 Limits: English, Humans

Abbreviation: MeSH, Medical Subject Headings.

### 2.6 Study Selection

Based on the inclusion and exclusion criteria (Table 1), two calibrated reviews (SS, QS) independently performed titles, abstracts, and keywords screening using the Rayyan Online Software (Qatar Computing Research Institute). Inter‐examiner calibration was achieved through open discussion and comparison between two reviewers (SS, QS) after independently assessing the first 100 records (approximately 8% of the total). Articles with unclear information regarding AR, MR, and VR technologies in the abstract but with the potential for relevant data in full text were included for full‐text analysis. No restrictions were made in terms of study design, as this review did not focus on treatment efficacy. However, at this stage, the language of included studies was restricted to English. Subsequently, both reviewers independently assessed the full‐text version of potentially eligible studies after calibrating their assessment on 20 articles. When disagreement regarding the inclusion of a specific article occurred, both reviewers had an open discussion. If no agreement was achieved, another co‐author (MB) made the final decision. Following article selection, Cohen's kappa coefficient (*k*) was calculated to assess the degree of inter‐examiner reliability.

### 2.7 Data Extraction and Management

Relevant information from the included articles was extracted using a pilot‐tested data extraction table (Excel, Microsoft Corporation) similar to the original SR, with minor modifications to align with technological advancements in the field (SS, TJ). After calibration and validation, data extraction was performed entirely by the research team. In case of missing relevant information in the main manuscript and in Supporting Information, the respective article was ultimately excluded from the review.

For each of the eligible studies the following data were recorded: (1) bibliographic information: first author, publication year, journal and country of origin; (2) study characteristics: study type, design, investigated filed, dental discipline and application context; (3) participants' characteristics: total number of participants and evaluated models or tests; (4) type of reality; (5) device characteristics: brand and model of the device; (6) main primary and secondary outcomes of the study, (8) outcome domain, and (9) key findings.

### 2.8 Data Analysis and Synthesis

All data analyses were performed by the research team, and all the data synthesis was performed by an experienced statistician (ES). Due to the large number of included studies and the significant heterogeneity in their research methodologies, design, aims and utilized technologies, for the purpose of data synthesis, studies were categorized according to the application settings (clinical or educational), target population and type of outcomes assessed. A narrative synthesis was employed to overall pool of received data within application settings. The scope of this study was not to compare interventions or their efficacy, rather to provide an overview. Therefore, the synthesis process began with a preliminary analysis, during which data were extracted and the outcomes organized into tabular form. This approach provided a comprehensive summary of the findings and facilitated the identification of potential patterns within the data.

The largest treatment approach was represented by the VR outcome trials. Therefore, frequentist pairwise meta‐analyses were conducted for the RCTs conducted over VR. To assess the high heterogeneity of the trials, the meta‐analyses were conducted over trial subgroups. For AR and MR technologies, a meta‐analysis was not feasible due to the smaller sample and substantial heterogeneity of the evaluated outcomes.

Data extraction for the quantitative analyses was restricted to objective, stress‐related clinical parameters, specifically post‐treatment heart rate (HR), clinician‐reported outcome measures and patient‐reported outcome measures (PROMs) for pain and anxiety. The selected RCTs evaluated different distraction techniques used during dental treatments, including VR, tell–show–do (TSD) and TV, across different age group patients, children and adults. Meta‐analyses were conducted to compare of VR with TSD and VR with TV, with results analyzed separately for children and adults. Means and standard deviations of HR measurements were extracted from each study. Statistical heterogeneity among the included RCTs was assessed using Cochran's [31] statistic to evaluate the presence of heterogeneity, and quantified the inconsistency index ($I^{2}$) [32], and between study variance ($\tau^{2}$). Based on these statistics, the use of a fixed‐effect or a random‐effect model was determined. Given the differences in patient characteristics and the several clinical procedures implemented, sensitive analyses were conducted to explore potential sources of heterogeneity. All analyses were performed using R statistical software (version 4.5.2; R Core Team, 2025). For the HR measurement, the analysis was based on mean differences (MD) and standard errors (SE). For the remaining outcomes, a standardized mean differences (SMD) and standard errors (SE) were considered. For the latter, the values were calculated based on Hedge's effect size formula [27, 29, 33, 34, 35].

Subgroup analyses were conducted based on intervention type and age group. Interventions were categorized according to their level of invasiveness into minimally invasive (injection‐based), intermediate invasive (injection‐based/restorative), and surgical or highly invasive procedures.

For age, pediatric studies were grouped into three categories to reflect increasing age ranges across trials. Due to variability in how age was reported and overlap in age ranges between studies, these categories were defined pragmatically to allow comparison rather than based on strict predefined age cut‐offs.

### 2.9 Assessment of Risk of Bias and Quality of Evidence

All included articles were independently evaluated by two reviewers (SS, QS) for overall risk of bias using the corresponding scale according to the study design. The Cochrane Collaboration's tool for assessing risk of bias (RoB 2) tool [36] was used for randomized clinical trials, the risk of bias in non‐randomized studies of interventions (ROBINS‐I) tool was utilized for prospective controlled clinical studies to assess the risk of bias assessment and the Joanna Briggs Institute Critical Appraisal tool was used for quasi‐experimental studies and for the cross‐sectional studies Joanna Briggs Institute Critical Appraisal tool was used for cross‐sectional studies.

The quality appraising of dental education research was performed with the Medical Education Research Study Quality Instrument (MERSQI) [37]. The MERSQI evaluates studies based on six domains (study design, sampling type of data, validity of the evaluated instruments or methods, data analysis and outcomes). Each of the domains is scored on a three‐point scale, giving a total score out of 18 for each study. Studies with a MERSQI ≤ 8 score were defined as lower methodological quality, between 9 to 12 as moderate and ≥ 13 score as a high methodological quality. Disagreement between reviewers was resolved by open discussion [38]. In case no agreement could be achieved, the final decision was made by another co‐author (MB).

The quality of evidence for each estimated effect size was evaluated using grading of recommendations assessments, development and evaluation (GRADE) guidelines [39], considering factors such as inconsistencies in study results, indirectness of evidence, and study imprecision. The GRADE guidelines were used to categorize heterogeneity, with < 40% indicating low, 30%–60% representing moderate, 50%–90% signifying substantial, and 75%–100% reflecting considerable heterogeneity [40].

## 3 Results

### 3.1 Study Selection

The study selection process is presented in the PRISMA flow‐chart in Figure 1. Due to the rapid development of technologies and slight modification of the eligibility criteria the PRISMA flow‐chart for a new systematic review was used and the results of initial and updated manuscripts were analyzed separately. The electronic search yielded a total of 1915 entries, of which 961 were retrieved in MEDLINE (via PubMed), 768 in EMBASE, and 186 in Cochrane Central Register of Controlled Trials (CENTRAL). Following duplicate removal, 1317 articles remained. After title and abstract screening, 287 articles were selected for full‐text analysis (kappa = 0.84). Of these, 177 publications were excluded (kappa = 0.82) for various reasons, of which the most frequent was inadequate study type (*n* = 110) and trail registered, not yet published (*n* = 33). The list of excluded articles and reasons for exclusion are displayed in Table S1. No further manuscripts were found through manual search and cross‐reference checking. Finally, 110 studies met the inclusion criteria and were eligible for further analysis.

**FIGURE 1:**
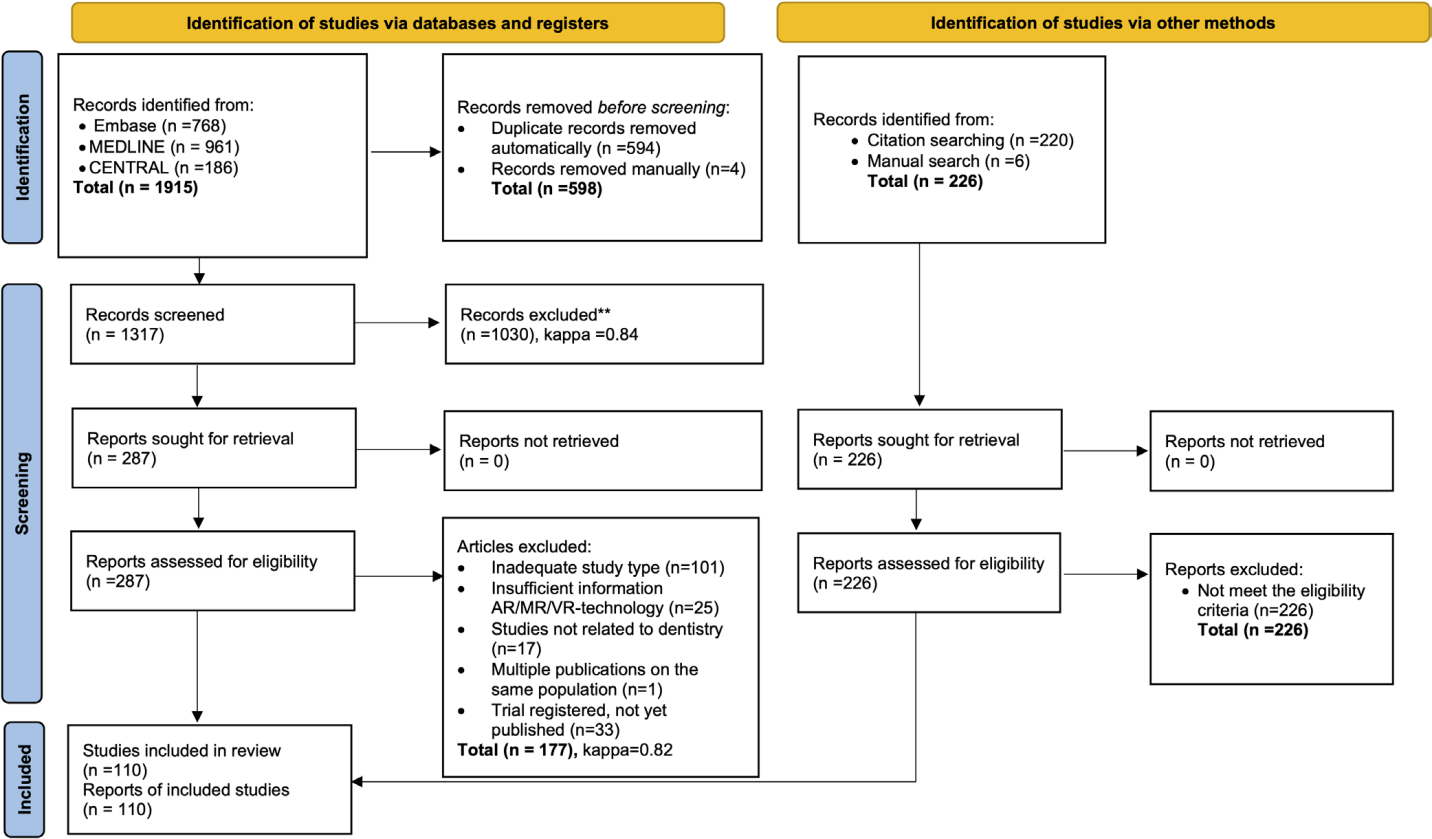
PRISMA 2020 flow diagram for new systematic reviews which included searches of databases, registers and other sources.

Overall, 110 articles reporting on 109 unique patient populations were included and a total of 8573 participants were evaluated in this updated systematic review.

### 3.2 Overall Characteristics of Included Studies

Study characteristics of included articles are presented in Table S2. Among them, eight were published in 2019, 10 in 2020, 14 in 2021, 14 in 2022, 15 in 2023, 32 in 2024, and 17 in 2025. A total of 62 studies were published in Asia, with the majority of 15 studies conducted in China and 11 trials in India. In Europe, 29 manuscripts were published, with Germany contributing the largest number of seven studies. In North America were performed 11 trials, all of them in the USA; in Africa, three studies; in Australia, three manuscripts; and in South America, two articles. A schematic representation of the differences is explained in Figure 2. The studies covered a wide range of dental disciplines, with the majority of 27 trials in pediatric dentistry. Fifteen were published in conservative dentistry, 15 studies in oral surgery, 14 manuscripts in prosthodontics, nine studies in endodontics, five articles in periodontology, five manuscripts in implant dentistry, five articles in restorative dentistry, four studies in general dentistry, four articles in dental radiology, four in anatomy, and three in orthodontics. A schematic illustration is presented in Figure 3. Of the included studies, 41 were clinical trials and 69 were educational studies. The most investigated technology was VR, reported in 94 studies. AR was examined in 11 trials, and MR in 2 manuscripts. Three studies tested multiple technologies. The most frequently used commercial systems included Oculus Quest (Facebook, USA), Simodont dental trainer (Moog, Netherlands), and the Virteasy dental simulator (Changé, France), while several studies employed self‐developed VR/AR systems. A graphical representation is presented in Figure 4.

**FIGURE 2:**
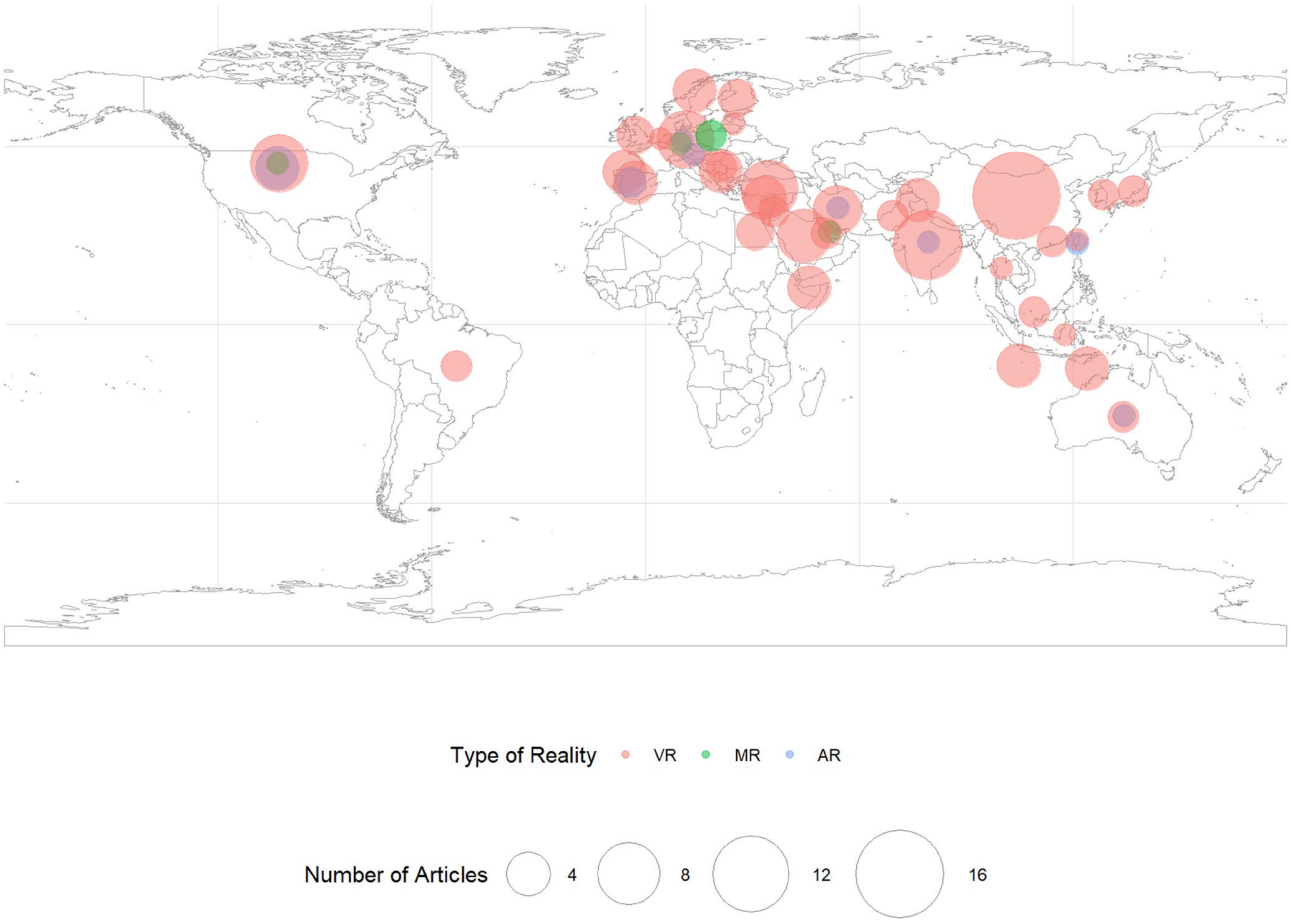
Geographic distribution of included studies according to the number of included studies and type of reality.

**FIGURE 3:**
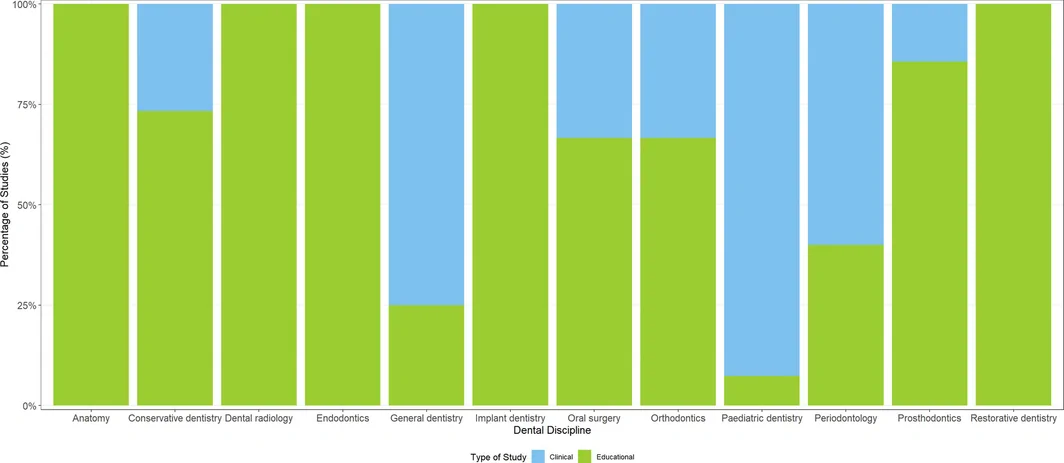
Distribution of included studies according to the study type and dental discipline.

**FIGURE 4:**
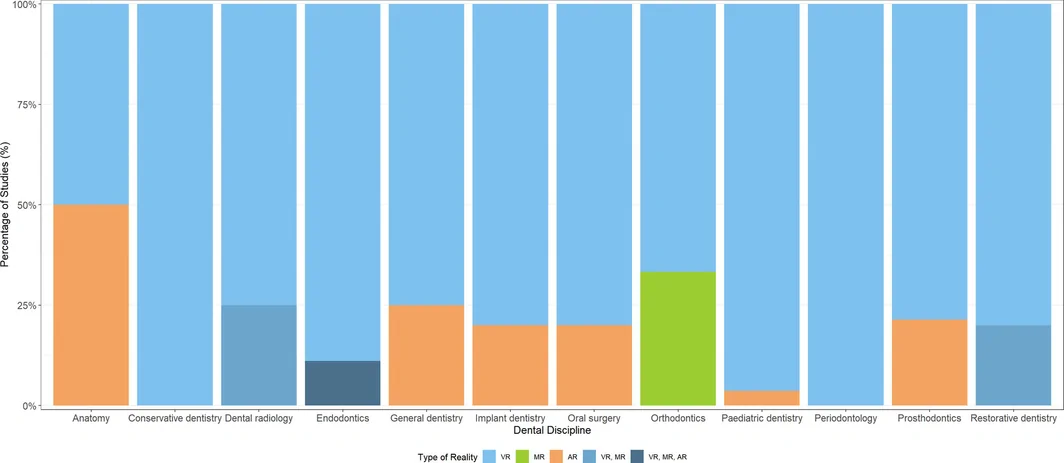
Distribution of included studies according to the type of reality and dental discipline.

The evaluated primary and secondary outcomes, as well as the scope of included studies, were highly heterogeneous. Based on the application domains, a total of 71 studies evaluated clinical or patient‐centered applications, 33 studies investigated operator‐ or clinician‐centered applications, and 132 manuscripts analyzed educational or training applications. Among primary outcomes, 30 manuscripts evaluated patient‐reported outcomes, seven clinical outcomes, seven clinician‐related outcomes, nine operator‐related outcomes, and 57 educational or training outcomes. Among secondary outcomes, 24 manuscripts evaluated patient‐reported outcomes, 10 clinical outcomes, 10 clinician‐reported outcomes, 7 operator‐related outcomes, and 59 educational or training outcomes.

### 3.3 Study Characteristics According to the Application Context

#### 3.3.1 Educational and Training Studies

Sixty‐eight studies investigated educational or training applications of immersive technologies, including 40 true experimental studies, 12 quasi‐experimental studies, 15 pre‐experimental trials, and one cross‐sectional survey. Sample sizes ranged from 20 to 264 participants. Most studies (*n* = 60) evaluated dental students across different years of undergraduate education, while seven studies included both dental students and dental practitioners, and one study exclusively assessed dental practitioners. Regarding the technologies investigated, 55 studies evaluated VR alone, two studies combined VR and MR, two studies focused on MR, eight studies investigated AR, and one study evaluated all immersive technologies. Studies were most frequently reported in prosthodontics and restorative dentistry, with 17 studies conducted in these fields.

Across educational trials, the majority reported positive effects of VR, AR, and MR on learning‐related outcomes. Common findings included improved theoretical knowledge, enhanced manual skills and simulation performance, increased learner engagement, and higher levels of student satisfaction. Haptic‐enabled simulators and immersive VR environments were frequently associated with improved objective performance metrics, reduced task completion times, and higher task scores. Nevertheless, substantial heterogeneity was observed in outcome measures and assessment methods across studies, limiting direct comparability of results.

#### 3.3.2 Clinical Studies

A total of 42 clinical studies were evaluated, including 36 RCTs, three non‐RCTs, two feasibility studies and one cross‐sectional study. Sample sizes ranged from 20 to 277 participants. Eighteen studies evaluated adult populations, while 24 studies included pediatric patients aged between 4 and 14 years, which explains the predominance of studies conducted in pediatric dentistry. Among pediatric studies, 23 evaluated VR‐based interventions and one RCT investigated AR technology. In these studies, VR was primarily used for intraoperative distraction and anxiety reduction, whereas AR was applied for oral hygiene education and behavior modification in children. The most frequently investigated procedures in pediatric populations were local anesthesia (13 trials) and various dental treatments (11 studies), including tooth extraction, pulpotomy, caries treatment, and oral hygiene procedures. In adult populations, VR was evaluated in 16 studies and AR in two studies. The most investigated interventions were local anesthesia (five trials), tooth extraction (five articles), scaling and root planning or other periodontal treatments (three studies), management of dental phobia (two studies), and smoking cessation (one trial). Clinical studies predominantly measured subjective patient‐reported outcomes such as anxiety and pain, as well as objective stress‐related clinical parameters, including heart rate and blood pressure, and the efficacy of VR/AR technologies. Educational studies commonly assessed learning and skill acquisition, student performance, and user acceptance. The principal outcome domains across included studies can be grouped as follows: learning and performance in 52 studies, anxiety, pain and stress reduction in 43 trials, simulation or technical performance in 20 manuscripts, and accuracy or precision of clinical tasks in nine studies.

Out of 36 RCTs, only those evaluating the same technologies in healthy participants and reporting comparable outcome measures were included in the pairwise meta‐analyses, while the remaining trials were synthesized qualitatively.

### 3.4 Data Analysis and Synthesis

Among the included RCTs, only studies evaluating VR interventions with HR as an outcome were initially selected, yielding a total of 16 RCTs. Of these, 12 (75.0%) compare VR with TSD, 4 (25.0%) compared VR with TV, and 10 trials (62.5%) involved pediatric populations, while 6 (37.5%) enrolled adult patients.

Clinician‐reported outcome measures were assessed in nine RCTs, the majority of which were conducted in pediatric patients (eight trials, 88.9%). Pain PROMs were reported in 13 RCTs, including 9 (69.23%) pediatric and 4 (30.77%) adult studies. Anxiety PROMs were evaluated in 14 RCTs, of which 11 (78.57%) involved children and 3 (21.43%) involved adults.

### 3.5 Pairwise Meta‐Analyses

A frequentist pairwise meta‐analysis (MA) considering a random effects model was conducted to evaluate the effect of various dental treatment interventions on patients' heart rate (HR) (Table S3). Firstly, the MA was conducted separately for each age group (children and adults). In the pediatric trials, seven studies (306 patients) compared VR with TSD, while the remaining four studies (206 patients) compared VR with TV. Most of the trials had a single comparison group, but one study included two groups, comparing both TSD and TV with VR.

In the comparison between VR and TSD (Figure 5), the pooled analysis using a random‐effects model showed a borderline statistically significant effect favoring VR (MD = −3.26, 95% CI −6.51 to −0.00; *p* = 0.0497). Considerable variability in effect estimates was indicated by substantial heterogeneity across studies (*I* ^2^ = 64.1%, *τ* ^2^ = 11.14, *p* = 0.010).

**FIGURE 5:**
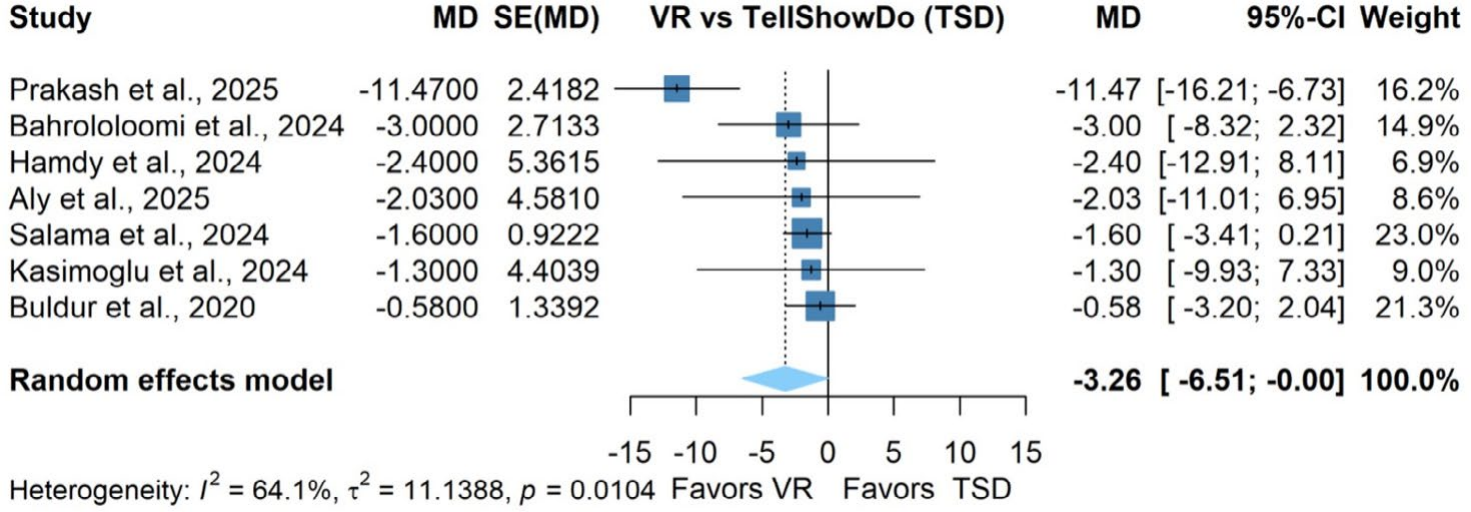
Meta‐analysis and forest plot between virtual reality (VR) and Tell‐Show‐Do techniques in relation to heart rate measured in pediatric patients (*k* = 7).

Leave‐one‐out sensitivity analysis demonstrated that exclusion of the study by Prakash et al. (2025) reduced the pooled effect size (MD = −1.43, 95% CI −2.81 to −0.04; *p* = 0.0433), while remaining statistically significant. Moreover, heterogeneity was no longer observed (*I* ^2^ = 0%), although the confidence interval for *I* ^2^ remained wide (0%–74.6%), indicating uncertainty due to the limited number of studies.

Excluding other individual studies did not alter the direction of the effect but resulted in a loss of statistical significance and the persistence of substantial heterogeneity. The study by Prakash et al. had the greatest impact on between‐study heterogeneity, with a larger effect size than the other included studies.

As for the subgroup analyses, intervention type did not significantly influence the effect (*p* = 0.869), whereas age‐based subgroup differences were observed but were largely driven by a single study [41].

No significant difference was found between VR and TV (MD = −0.02, 95% CI −8.95 to 8.91), with considerable heterogeneity (*I* ^2^ = 89.7%) and inconsistent results across studies (Figure S1). For studies with adult patients, six RCTs (577 patients) (Figure S2) were included comparing VR with TSD. The pooled analysis showed no statistically significant difference between interventions (MD = −3.01, 95% CI −7.20 to 1.17; *p* = 0.158), with substantial heterogeneity across studies (*I* ^2^ = 91.3%, *τ* ^2^ = 22.13, *p* < 0.001). The direction and magnitude of effects varied considerably among studies. Leave‐one‐out sensitivity analysis indicated that exclusion of one study reduced heterogeneity (*I* ^2^ = 49.8%) and weakened the pooled effect (MD = −1.23, 95% CI −4.33 to 1.86), although results remained non‐significant.

Subgroup analysis by intervention type did not show significant differences between subgroups (*p* = 0.208), although estimates were based on a limited number of studies within each category. Further analyses were performed for clinical‐reported outcomes and patient‐related outcomes (PROMs), such as pain and anxiety. Clinical‐reported outcomes were analyzed jointly for children and adult patients across seven RCTs (460 patients) (Figure S3). The pooled standardized mean difference showed no statistically significant difference between VR and TSD (SMD = −0.81, 95% CI −1.86 to 0.23; *p* = 0.126). There was considerable heterogeneity across studies (*I* ^2^ = 95.1%, *τ* ^2^ = 1.89, *p* < 0.001), with substantial variability in both the magnitude and direction of effects. As for the subgroup analyses, neither age group nor intervention type had a significant influence on the effects (*p* = 0.08; *p* = 0.98, respectively). No significant difference was observed between VR and TV (SMD = −0.41, 95% CI −1.15 to 0.32), with substantial heterogeneity (*I* ^2^ = 82.2%) (Figure S4).

PROMs were analyzed separately by age group. Statistically significant differences were observed only for anxiety‐related PROMs in studies for the comparison between VR and TSD involving children (Figure 6). In children, ten comparisons (418 patients) were included. The pooled effect remained significant in favor of VR (SMD = −1.32, 95% CI −1.93 to −0.72; *p* < 0.001), with high heterogeneity (*I* ^2^ = 89.5%, *τ* ^2^ = 0.86, *p* < 0.001). Subgroup analysis by intervention type showed significant differences between subgroups (*p* < 0.001), with a larger effect observed in surgical/highly invasive procedures (SMD = −2.72, 95% CI −3.30 to −2.14) than in intermediate invasive procedures (SMD = −0.98, 95% CI −1.48 to −0.49). In contrast, subgrouping by age category did not show significant differences (*p* = 0.392).

**FIGURE 6:**
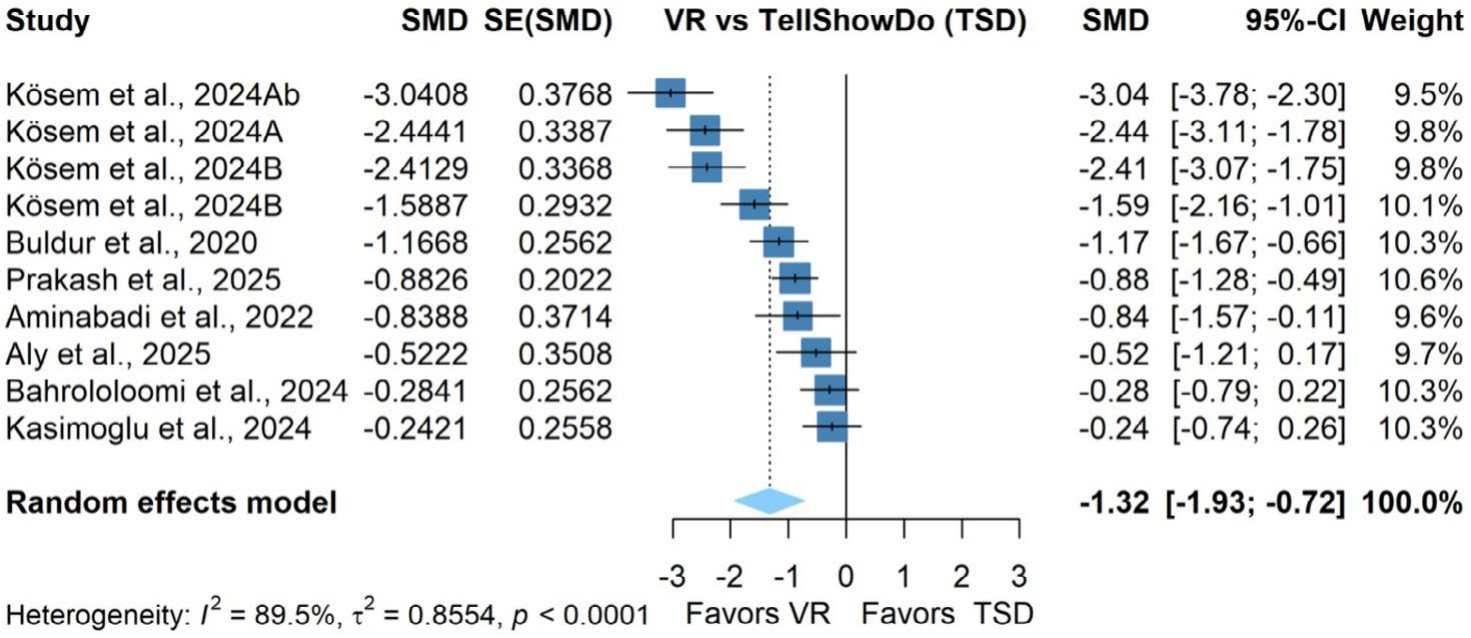
Meta‐analysis and forest plot between virtual reality (VR) and Tell‐Show‐Do (TSD) techniques in relation to patient reported outcomes anxiety levels measured in pediatric patients (*k* = 10).

In adults, three (439 patients) studies were included (Figure S5). The pooled estimate showed a non‐significant trend favoring VR (SMD = −0.73, 95% CI −1.51 to 0.06; *p* = 0.070), again with substantial heterogeneity (*I* ^2^ = 90.6%, *τ* ^2^ = 0.45, *p* < 0.001).

For the comparison between VR and TV, only two studies were available. The pooled estimate showed a non‐significant trend favoring VR (SMD = −0.96, 95% CI −2.02 to 0.09; *p* = 0.074), with substantial heterogeneity (*I* ^2^ = 87.0%).

Seven studies (396 patients) were included in the pediatric analysis to compare pain levels VR with TSD (Figure S6). The pooled standardized mean difference showed no statistically significant difference between VR and TSD (SMD = −0.65, 95% CI −1.57 to 0.26; *p* = 0.163), although the direction of effect favored VR. There was considerable heterogeneity across studies (*I* ^2^ = 96.2%, *τ* ^2^ = 1.43, *p* < 0.001), with substantial variability in both the magnitude and direction of effects.

Subgroup analysis by age did not show significant differences between groups (*p* = 0.147). Subgroup analysis by intervention type showed statistically significant differences (*p* = 0.001), although this was driven by subgroups containing single studies, limiting interpretability (Figure S7).

Leave‐one‐out analysis indicated that exclusion of one study resulted in a statistically significant effect favoring VR (SMD = −1.05, 95% CI −1.64 to −0.47), with reduced heterogeneity (*I* ^2^ = 87.8%), suggesting this study contributed substantially to the observed inconsistency.

For the adults' trials, four studies (302 patients) were included in the analysis to compare pain levels of VR with TSD (Figure S8). The pooled estimate showed a borderline non‐significant effect favoring VR (SMD = −0.54, 95% CI −1.11 to 0.02; *p* = 0.059), with substantial heterogeneity (*I* ^2^ = 84.8%, *τ* ^2^ = 0.29, *p* < 0.001).

Sensitivity analysis indicated that exclusion of one study reduced heterogeneity (*I* ^2^ = 45.4%) and attenuated the effect size (SMD = −0.29, 95% CI −0.58 to 0.01), although results remained non‐significant. For the comparison between VR and TV for pain PROMs, only one study was available; therefore, no meta‐analysis was performed (Figure S9).

### 3.6 Risk of Bias Assessment and Certainty of Evidence Assessment

The quality assessment of the 36 included RCTs using RoB2 risk of bias assessment tool is illustrated on Figure 7. Nine RCTs were judged to have an overall low risk of bias, while 24 RCTs rated as having some concerns. Three RCTs had a high risk of bias, primarily due to the issues arising from the randomization process (domain 1) and selective reporting (domain 5).

**FIGURE 7:**
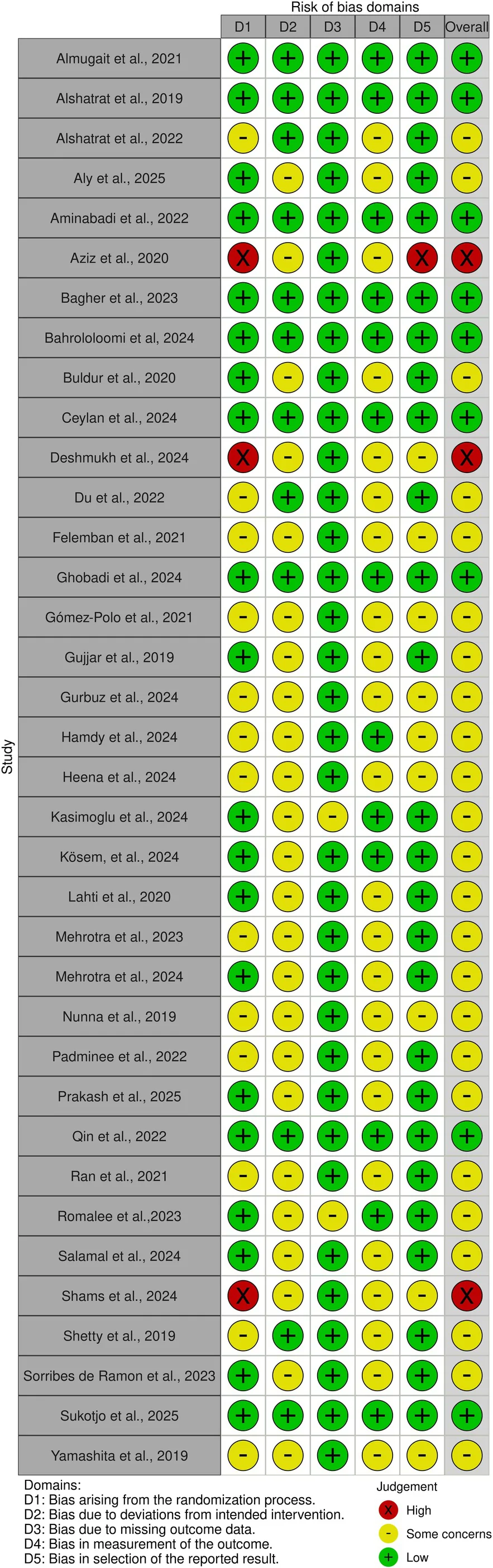
Risk of bias assessment for randomized controlled trials.

The methodological quality of the included 68 educational studies, assessed with the MERSQI tool, was overall moderate. The complete risk of bias assessment is displayed in Table S4. Most articles achieved higher scores in the domains of study design, type of data, and data analysis, whereas lower scores were observed for validity evidence of assessment instruments and outcome levels. The complete risk of bias assessment for the remaining studies is displayed in Figure S10, Tables S5 and S6.

In addition, the certainty of evidence was evaluated using the GRADE approach. Given that the analysis was conducted as a pairwise meta‐analysis, the standard GRADE framework was applied. The certainty of evidence was rated across the domains of risk of bias, inconsistency, indirectness, imprecision, and publication bias.

## 4 Discussion

This systematic review and pairwise meta‐analysis update the findings of the original publication [22] and expand the evidence summarized in previously published scoping reviews of our group [6, 10] (Table S7). Overall, the present results agree with most of the findings of the original systematic review (SR). Both studies highlight:

- The growing research interest in augmented reality (AR), mixed reality (MR), and virtual reality (VR) technologies in dental medicine, with most published studies focusing on under‐ and postgraduate dental education.
- VR technologies were evaluated more frequently than AR and MR, with the most clinical studies focusing on VR applications as a distraction method during dental treatment in both pediatric and adult populations.

The original SR was published in 2019 and focused on various AR and VR applications in dentistry, maxillofacial surgery, and dental education. It included 16 articles published between 2013 and 2018 in a peer‐reviewed journal. Given the rapid development of digital and immersive technologies and the introduction of new regulatory frameworks, the current SR adopted definitions in accordance with the Glossary of Computer‐assisted Implant Surgery and related terms [25] and the US Food and Drug Administration [26]. To reduce the heterogeneity and ensure higher methodological standards, only articles directly related to dentistry and dental education were included in the updated SR. Specifically, studies evaluating AR, MR, and VR technologies in dental medicine and/or under‐ or postgraduate dental education were analyzed. In total, 110 studies published from 2019 to 2025, encompassing 8573 participants, were evaluated. Among them, 94 studies utilized VR technologies. The prominent increase in publication activity over recent years reflects the rapid development of this area. This trend shows both technological advancements and growing integration of immersive systems into dental medicine and education. Despite this promising tendency, the overall number of high‐quality clinical studies, as RCTs, remains relatively low. The overall strength of evidence was rated as low to moderate according to GRADE guidelines mainly due to the high heterogeneity in the research methodology [39].

In this systematic review and pairwise meta‐analysis, most of the included articles were conducted in Asia, followed by Europe. A similar geographic distribution was observed in the original publication. These findings are in line with a recently published scoping review evaluating the utilization of AR and MR technologies in a narrow clinical domain of implant dentistry in eight clinical studies [6]. Likewise, another SR limited to the focus on VR technologies in dental education also highlighted a substantial representation of studies from Asian and European countries [42]. Current trends might reflect greater governmental support for healthcare and dental technological innovations in several Asian countries. It can be hypothesized that dentists, patients, students, and other stakeholders in these regions demonstrate a higher level of openness to adopt digital technologies and innovations.

Overall, the obtained findings show a trend towards immersive technologies being associated with improvements in both educational and clinical outcomes. These results are in line with recently published SR [28, 29], further supporting the growing evidence in this area. However, AR, MR, or VR technologies are relatively new and still require appropriate learning and adaptation. It is crucial that both dentists and patients understand how to effectively use these technologies in clinical practice to improve treatment results.

Immersive technologies have been applied across various dental disciplines, with the majority of 27 included studies performed in pediatric dentistry. One possible explanation for this focus is the well‐recognized challenge in managing children's anxiety and ensuring good cooperation during dental treatment. Several RCTs compared different behavior and distraction approaches, such as TSD [43], app [44], television [45], clown [41], and VR to reduce pain and anxiety. By different measures, VR showed a consistent tendency towards improving patients' experience compared with conventional behavioral techniques. However, the results varied depending on the population and the investigated clinical, clinician‐ and patient‐reported outcomes. For physiological outcomes such as heart rate, a borderline statistically significant effect favoring VR was observed in pediatric populations. However, this finding was influenced by a single study which substantially contributed to heterogeneity (*I* ^2^ = 64.1%). Nevertheless, sensitivity analysis showed that the direction of the effect remained consistent. In contrast, no significant differences were found for heart rate in adult populations or for comparisons between VR and TV, with substantial heterogeneity (*I* ^2^ often exceeding 80%–90%). Similarly, pain and clinician‐reported outcomes did not reach statistical significance, although most analyses indicated a trend favoring VR and were frequently influenced by individual studies.

Notably, anxiety levels demonstrated a statistically significant reduction with VR, particularly in children, with stronger effects observed in more invasive procedures. However, even for this outcome, heterogeneity remained relatively high. Across all analyses, subgroup effects were generally inconsistent or driven by small subgroups or single studies, limiting their interpretability. Overall, the substantial heterogeneity, variability in study characteristics, and dependence on individual trials reduce confidence in the pooled estimates, highlighting the need for more standardized and methodologically robust studies.

An original publication highlighted that AR and VR technologies offer benefits for both clinical and educational applications. Similar findings were observed in the present updated SR. Among 110 included studies, 64 manuscripts focused on educational purposes, consistently demonstrating improvements in theoretical knowledge, manual skill acquisition, learner engagement, and higher students' satisfaction. This alignment of findings across diverse educational contexts, from haptic‐enabled simulators to immersive VR scenarios, integrated into various disciplines of the dental curriculum—underscores the potential broad applicability of these technologies. Notably, VR provides 24/7 access to the learning materials, which offers students greater flexibility and increased responsibility for self‐directed learning. Such competencies are essential for clinical practice. Immersive technologies are promising for dental education [46]. They are progressively being integrated into under‐ and postgraduate education across the world. However, the long‐term impact of these technologies on the quality of specialist training and clinical outcomes remains insufficiently explored and needs to be further investigated.

Despite promising results of AR, MR, and VR technologies, the appropriate target group needs to be used for their implementation. Moreover, implementation of immersive technologies involves substantial financial costs related to equipment, software, training, and maintenance. Therefore, rigorous cost–benefit analysis supported by robust scientific evidence is essential to justify and facilitate the adoption of these technologies into university curriculum and daily clinical practice.

### 4.1 Limitations

Several important limitations need to be acknowledged. Included articles were heterogenous in terms of study design as well as utilized methodology. Moreover, the exclusion of studies with fewer than 15 participants per arm (or per sample), while intended to reduce the influence of underpowered trials, may introduce a potential source of selection bias and exclude valuable proof‐of‐concept studies, particularly in emerging AR and MR technologies. The restriction to English‐language publications introduces potential geographic and cultural bias, particularly relevant given the global nature of technological innovation in healthcare. Moreover, a considerable number of included studies were assessed as having high or moderate risk of bias.

The heterogeneity in utilized devices, outcome measures, and assessment methods across studies reflects the rapidly evolving technological landscape and lack of standardized protocols. This variability complicates data synthesis and clinical implementation. It further limited the analysis, as the meta‐analyses could only be conducted for VR on a small number of RCTs and was characterized by substantial heterogeneity. In contrast, the number of AR and MR studies was insufficient, emphasizing the need for consensus guidelines on optimal hardware specifications, software requirements, and evaluation frameworks.

In particular, the RCTs included in the meta‐analyses varied considerably in terms of clinical procedures, patient characteristics, outcome definitions, and VR implementation. Although intervention‐specific and age‐stratified analyses were conducted where feasible, substantial residual clinical and methodological heterogeneity likely reduced the precision of pooled effect estimates and limited the reliability of indirect and network comparisons.

### 4.2 Future Outlook

The integration of AR, MR, and VR technologies with existing digital workflows offers significant opportunities to enhance patient care and clinical efficiency. The synergetic use of immersive technologies with intraoral scanning, digital imaging, computer‐assisted implant surgery (CAIS), and computer‐aided design/manufacturing (CAD/CAM) systems could strengthen multidisciplinary treatment planning, facilitate expectation management and patient communication, and improve treatment results across multiple dental disciplines. Moreover, integration of VR into dental treatment workflows, such as CAD/CAM prosthetics, implant planning, and CAIS, appears very promising. MR's ability to combine real‐world clinical interactions with digital environment could address limitations of purely virtual surroundings while maintaining immersive benefits. To obtain robust results, future research should prioritize well‐designed, adequately powered RCTs with standardized outcome measures, as well as more homogeneous patient populations and clinical procedures. This will strengthen the reliability of comparative effectiveness estimates.

To sum up, technological progress is not following a linear trajectory but rather evolves through rapid, exponential jumps. Accordingly, regularly updated systematic reviews are essential for capturing and monitoring developments in this field.

## 5 Conclusions

This updated systematic review with meta‐analysis indicates that augmented reality (AR), mixed reality (MR), and virtual reality (VR) technologies are increasingly applied in dental medicine and education, with most published studies focusing on under‐ and postgraduate training.

### 5.1 Implications for Clinical Practice

Current evidence shows a trend towards the effectiveness of immersive technologies, particularly VR, in improving patient experience, especially through anxiety and pain management, and in enhancing learning outcomes for dental students. However, these findings should be interpreted with caution due to heterogeneity in study designs, populations, interventions, and outcome measures, as well as the limited number of high‐quality randomized controlled trials. Overall, the certainty of evidence is low to moderate. Although AR, MR, and VR technologies show promising results for clinical application, the existing evidence is insufficient to support definitive clinical recommendations.

### 5.2 Implications for Research

Future studies should focus on well‐designed, adequately powered randomized clinical trials with clearly defined indications, standardized protocols, and prespecified outcome measures. Such studies are necessary to strengthen the evidence base and enable the development of evidence‐based clinical recommendations for use of AR, MR, and VR technologies.

## Author Contributions

**Tim Joda:** conceptualization, methodology, resources, supervision, writing – reviewing and editing. **Sofya Sadilina:** data curation, project administration, investigation, visualization, writing – original draft preparation. **Eugenia Settecase:** formal analysis, validation, visualization, writing – original draft reviewing and editing. **Ronald E. Jung:** conceptualization, writing – reviewing and editing; **Syed Majeed Quadri:** data curation, software, investigation, writing – original draft preparation. **Marc Balmer:** conceptualization, funding acquisition, software, writing – reviewing and editing.

## Funding

The present study was funded by the authors and their institutions.

## Ethics Statement

Ethical approval was not required for this systematic review and pairwise meta‐analysis.

## Conflicts of Interest

The authors declare no conflicts of interest.

## Supporting information


**Figure S1:** Meta‐analysis and forest plot between VR and TV techniques in relation to heart rate measured in pediatric patients (*k* = 4).
**Figure S2:** Meta‐analysis and forest plot between virtual reality (VR) and Tell–Show–Do (TSD) techniques in relation to heart rate measured in adult patients (*k* = 6).
**Figure S3:** Meta‐analysis and forest plot between VR and Tell‐Show‐Do techniques in relation to clinicians reported outcomes measured in adults and pediatric patients (*k* = 7).
**Figure S4:** Meta‐analysis and forest plot between VR and TV techniques in relation to clinicians reported outcomes measured in pediatric patients (*k* = 3).
**Figure S5:** Meta‐analysis and forest plot between VR and Tell‐Show‐Do techniques in relation to patient reported outcomes anxiety levels measured in adult patients (*k* = 3).
**Figure S6:** Meta‐analysis and forest plot between VR and Tell‐Show‐Do techniques in relation to patient reported outcomes pain levels measured in pediatric patients (*k* = 7).
**Figure S7:** Meta‐analysis and forest plot between VR and Tell‐Show‐Do techniques in relation to patient reported outcomes anxiety levels measured in pediatric patients ‐subgrouping analysis by intervention (*k* = 9).
**Figure S8:** Meta‐analysis and forest plot between VR and Tell‐Show‐Do techniques in relation to patient reported outcomes pain levels measured in adult patients (*k* = 4).
**Figure S9:** Meta‐analysis and forest plot between VR and TV techniques in relation to patient reported outcomes anxiety levels measured in pediatric patients (*k* = 2).
**Figure S10:** Risk of bias assessment of the non‐randomized studies based in ROBINS‐I tool.
**Table S1:** Excluded studies after full texts analysis and reasons for exclusion.
**Table S2:** Study characteristics of included studies.
**Table S3:** Study characteristics of included studies included in the network meta‐analysis.
**Table S4:** Risk of bias assessment for the educational studies with the Medical Education Research Study Quality Instrument.
**Table S5:** Risk of bias assessment for quasi‐experimental studies the Joanna Briggs Institute Critical Appraisal tool.
**Table S6:** Risk of bias assessment for cross‐sectional studies the Joanna Briggs Institute Critical Appraisal tool.
**Table S7:** Comparative table between current updated systematic review with pairwise meta‐analysis and scoping reviews.

## Data Availability

The datasets used in the study are available from the corresponding author upon reasonable request.

## References

[1] M. Romandini , E. Ruales‐Carrera , S. Sadilina , C. H. F. Hämmerle , and M. Sanz , “Minimal Invasiveness at Dental Implant Placement: A Systematic Review With Meta‐Analyses on Flapless Fully Guided Surgery,” Periodontology 2000 91, no. 1 (2023): 89–112. 10.1111/prd.12440 35906928

[2] J. Y. Shi , X. Y. Wu , X. L. Lv , et al., “Comparison of Implant Precision With Robots, Navigation, or Static Guides,” Journal of Dental Research 104, no. 1 (2025): 37–44. 10.1177/00220345241285566 PMC11667196 39586816

[3] T. Joda , M. M. Bornstein , R. E. Jung , M. Ferrari , T. Waltimo , and N. U. Zitzmann , “Recent Trends and Future Direction of Dental Research in the Digital Era,” International Journal of Environmental Research and Public Health 17, no. 6 (2020): 1987. 10.3390/ijerph17061987 PMC7143449 32197311

[4] A. Lanis , P. Llorens , and O. Alvarez Del Canto , “Selecting the Appropriate Digital Planning Pathway for Computer‐Guided Implant Surgery,” International Journal of Computerized Dentistry 20, no. 1 (2017): 75–85. 28294207

[5] W. Ricciardi , P. Pita Barros , A. Bourek , et al., “How to Govern the Digital Transformation of Health Services,” European Journal of Public Health 29, no. Suppl 3 (2019): 7–12. 10.1093/eurpub/ckz165 PMC6859522 31738442

[6] S. Sadilina , A. Unkovskiy , R. Jung , M. Balmer , and T. Joda , “Augmented and Mixed Reality in Implant Dentistry: A Scoping Review Exploring Clinician‐ and Patient‐Reported Outcomes,” International Journal of Computerized Dentistry (2026), 10.3290/j.ijcd.b6952432. 41805871

[7] H. N. Mai , H. C. Ngo , S. H. Cho , C. P. Duong , H. Y. Mai , and D. H. Lee , “Effectiveness of Virtual Reality Interactive Simulation Practice in Prosthodontic Education: A Systematic Review and Meta‐Analysis,” European Journal of Dental Education: Official Journal of the Association for Dental Education in Europe 29 (2024): 594–607. 10.1111/eje.12997 38433575

[8] A. Towers , J. Field , C. Stokes , S. Maddock , and N. Martin , “A Scoping Review of the Use and Application of Virtual Reality in Pre‐Clinical Dental Education,” British Dental Journal 226, no. 5 (2019): 358–366. 10.1038/s41415-019-0041-0 30850794

[9] S. Sadilina , K. Vietor , R. Doliveux , et al., “Beyond Accuracy: Clinical Outcomes of Computer Assisted Implant Surgery,” Clinical and Experimental Dental Research 11, no. 3 (2025): e70129. 10.1002/cre2.70129 PMC12081946 40375737

[10] S. Sadilina , F. J. Strauss , R. E. Jung , T. Joda , and M. Balmer , “Use of Optical See‐Through Head‐Mounted Display (OST‐HMD) in Dentistry: A Scoping Review,” International Journal of Computerized Dentistry 28, no. 2 (2025): 101–116. 10.3290/j.ijcd.b5394865 38801193

[11] H. H. M. Elhag , G. K. L. Rao , S. N. F. B. M. Noor , M. N. B. Zakaria , and N. B. Mokhtar , “Augmented Reality in Dental Implants: A Systematic Review,” Journal of Clinical and Diagnostic Research 18, no. 1 (2024): ZE20–ZE25.

[12] M. Kivovics , A. Takacs , D. Penzes , O. Nemeth , and E. Mijiritsky , “Accuracy of Dental Implant Placement Using Augmented Reality‐Based Navigation, Static Computer Assisted Implant Surgery, and the Free‐Hand Method: An In Vitro Study,” Journal of Dentistry 119 (2022): 104070. 10.1016/j.jdent.2022.104070 35183695

[13] T. Joda , M. Balmer , R. E. Jung , and A. Ioannidis , “Clinical Use of Digital Applications for Diagnostic and Treatment Planning in Prosthodontics: A Scoping Review,” Clinical Oral Implants Research 35 (2023): 782–792. 10.1111/clr.14230 38140771

[14] M. Almugait and A. AbuMostafa , “Comparison Between the Analgesic Effectiveness and Patients' Preference for Virtual Reality vs. Topical Anesthesia Gel During the Administration of Local Anesthesia in Adult Dental Patients: A Randomized Clinical Study,” Scientific Reports 11, no. 1 (2021): 23608. 10.1038/s41598-021-03093-2 PMC8654925 34880344

[15] D. Martinez‐Bernal , C. Vidovich , C. Keenan , et al., “The Use of Virtual Reality to Reduce Pain and Anxiety in Surgical Procedures of the Oral Cavity: A Scoping Review,” Journal of Oral and Maxillofacial Surgery 81, no. 4 (2023): 467–482. 10.1016/j.joms.2022.11.011 36572388

[16] Y. Huang , X. Cheng , U. Chan , et al., “Virtual Reality Approach for Orthodontic Education at School of Stomatology, Jinan University,” Journal of Dental Education 86, no. 8 (2022): 1025–1035. 10.1002/jdd.12915 35307835

[17] J. Lee , H. Kim , and F. Kron , “Virtual Education Strategies in the Context of Sustainable Health Care and Medical Education: A Topic Modelling Analysis of Four Decades of Research,” Medical Education 58, no. 1 (2024): 47–62. 10.1111/medu.15202 37794709

[18] A. Ayoub and Y. Pulijala , “The Application of Virtual Reality and Augmented Reality in Oral & Maxillofacial Surgery,” BMC Oral Health 19, no. 1 (2019): 238. 10.1186/s12903-019-0937-8 PMC6839223 31703708

[19] J. Y. Shi , B. L. Liu , X. Y. Wu , et al., “Improved Positional Accuracy of Dental Implant Placement Using a Haptic and Machine‐Vision‐Controlled Collaborative Surgery Robot: A Pilot Randomized Controlled Trial,” Journal of Clinical Periodontology 51, no. 1 (2024): 24–32. 10.1111/jcpe.13893 37872750

[20] N. Y. Tak , H. J. Lim , D. S. Lim , Y. S. Hwang , and I. H. Jung , “Effect of Self‐Learning Media Based on 360 Degrees Virtual Reality for Learning Periodontal Instrument Skills,” European Journal of Dental Education 27, no. 1 (2023): 1–8. 10.1111/eje.12769 35000247

[21] M. Chandra , K. Kumar , P. Thakur , S. Chattopadhyaya , F. Alam , and S. Kumar , “Digital Technologies, Healthcare and Covid‐19: Insights From Developing and Emerging Nations,” Health and Technology 12, no. 2 (2022): 547–568. 10.1007/s12553-022-00650-1 PMC8898601 35284203

[22] T. Joda , G. O. Gallucci , D. Wismeijer , and N. U. Zitzmann , “Augmented and Virtual Reality in Dental Medicine: A Systematic Review,” Computers in Biology and Medicine 108 (2019): 93–100. 10.1016/j.compbiomed.2019.03.012 31003184

[23] M. J. Page , J. E. McKenzie , P. M. Bossuyt , et al., “The PRISMA 2020 Statement: An Updated Guideline for Reporting Systematic Reviews,” Journal of Clinical Epidemiology 88 (2021): 105906. 10.1016/j.jclinepi.2021.03.001 33789819

[24] J. Chandler , M. Cumpston , T. Li , M. J. Page , and V. Welch , Cochrane Handbook for Systematic Reviews of Interventions (Wiley, 2019). 4(1002):14651858.

[25] A. Jorba‐Garcia , A. Pozzi , Z. Chen , et al., “Glossary of Computer‐Assisted Implant Surgery and Related Terms. First Edition,” Clinical and Experimental Dental Research 11, no. 4 (2025): e70148. 10.1002/cre2.70148 PMC12229244 40618393

[26] Food US. Drug administration (FDA) . (2021).

[27] R. Bjelovucic , J. Wolff , S. E. Norholt , R. Pauwels , and P. Taneja , “Effectiveness of Mixed Reality in Oral Surgery Training: A Systematic Review,” Sensors (Basel) 25, no. 13 (2025): 3945. 10.3390/s25133945 PMC12251658 40648202

[28] H. N. Mai , H. C. Ngo , S. H. Cho , C. P. Duong , H. Y. Mai , and D. H. Lee , “Effectiveness of Virtual Reality Interactive Simulation Practice in Prosthodontic Education: A Systematic Review and Meta‐Analysis,” European Journal of Dental Education 29, no. 3 (2025): 594–607. 10.1111/eje.12997 38433575

[29] Y. A. Algarni , R. S. Saini , S. K. Vaddamanu , et al., “The Impact of Virtual Reality Simulation on Dental Education: A Systematic Review of Learning Outcomes and Student Engagement,” Journal of Dental Education 88, no. 11 (2024): 1549–1562. 10.1002/jdd.13619 38807268

[30] D. X. Barros Padilha , N. J. Veiga , A. C. V. Mello‐Moura , and P. Nunes Correia , “Virtual Reality and Behaviour Management in Paediatric Dentistry: A Systematic Review,” BMC Oral Health 23, no. 1 (2023): 995. 10.1186/s12903-023-03595-7 PMC10717698 38087294

[31] W. G. Cochran , “The Comparison of Percentages in Matched Samples,” Biometrika 37, no. 3/4 (1950): 256–266. 14801052

[32] S. Balduzzi , G. Rücker , A. Nikolakopoulou , et al., “Netmeta: An R Package for Network Meta‐Analysis Using Frequentist Methods,” Journal of Statistical Software 106 (2023): 1–40.

[33] L. V. Hedges , “Distribution Theory for Glass's Estimator of Effect Size and Related Estimators,” Journal of Educational Statistics 6, no. 2 (1981): 107–128.

[34] B. Rouse , A. Chaimani , and T. Li , “Network Meta‐Analysis: An Introduction for Clinicians,” Internal and Emergency Medicine 12, no. 1 (2017): 103–111. 10.1007/s11739-016-1583-7 PMC5247317 27913917

[35] J. P. Higgins , S. G. Thompson , J. J. Deeks , and D. G. Altman , “Measuring Inconsistency in Meta‐Analyses,” BMJ 327, no. 7414 (2003): 557–560. 10.1136/bmj.327.7414.557 PMC192859 12958120

[36] J. A. C. Sterne , J. Savovic , M. J. Page , et al., “RoB 2: A Revised Tool for Assessing Risk of Bias in Randomised Trials,” BMJ 366 (2019): l4898. 10.1136/bmj.l4898 31462531

[37] D. A. Cook and D. A. Reed , “Appraising the Quality of Medical Education Research Methods: The Medical Education Research Study Quality Instrument and the Newcastle‐Ottawa Scale‐Education,” Academic Medicine 90, no. 8 (2015): 1067–1076. 10.1097/ACM.0000000000000786 26107881

[38] A. A. Mitchell and E. R. Ivimey‐Cook , “Technology‐Enhanced Simulation for Healthcare Professionals: A Meta‐Analysis,” Frontiers in Medicine (Lausanne) 10 (2023): 1149048. 10.3389/fmed.2023.1149048 PMC10150956 37138752

[39] H. J. Schunemann , A. D. Oxman , J. Brozek , et al., “Grading Quality of Evidence and Strength of Recommendations for Diagnostic Tests and Strategies,” BMJ (Clinical Research ed.) 336, no. 7653 (2008): 1106–1110. 10.1136/bmj.39500.677199.AE PMC2386626 18483053

[40] G. H. Guyatt , A. D. Oxman , R. Kunz , et al., “GRADE Guidelines: 7. Rating the Quality of Evidence–Inconsistency,” Journal of Clinical Epidemiology 64, no. 12 (2011): 1294–1302. 10.1016/j.jclinepi.2011.03.017 21803546

[41] S. Prakash , N. Sheoran , B. G. Saraf , V. Sharma , W. Khan , and M. Shaw , “Comparison of Effectiveness of Virtual Reality and Clown Dentists as Anxiety Management Tools Among 3‐13‐Year‐Old Children for Dental Procedures: A Randomized Controlled Trial,” International Journal of Clinical Pediatric Dentistry 18, no. 1 (2025): 105–113. 10.5005/jp-journals-10005-2972 PMC11915406 40110446

[42] H. Koolivand , M. M. Shooreshi , R. Safari‐Faramani , et al., “Comparison of the Effectiveness of Virtual Reality‐Based Education and Conventional Teaching Methods in Dental Education: A Systematic Review,” BMC Medical Education 24, no. 1 (2024): 8. 10.1186/s12909-023-04954-2 PMC10765860 38172742

[43] L. A. Sorribes De Ramón , A. F. Ferrández Martínez , A. R. García Carricondo , F. Espín Gálvez , and R. Alarcón Rodríguez , “Effect of Virtual Reality and Music Therapy on Anxiety and Perioperative Pain in Surgical Extraction of Impacted Third Molars,” Journal of the American Dental Association 154, no. 3 (2023): 206–214. 10.1016/j.adaj.2022.11.008 36707274

[44] K. Padminee , R. Hemalatha , P. Shankar , D. Senthil , T. G. Jayakaran , and S. Kabita , “Effectiveness of Biofeedback Relaxation and Audio‐Visual Distraction on Dental Anxiety Among 7‐ to 12‐Year‐Old Children While Administering Local Anaesthesia: A Randomized Clinical Trial,” International Journal of Paediatric Dentistry 32, no. 1 (2022): 31–40. 10.1111/ipd.12787 33735517

[45] S. M. Bagher , O. M. Felemban , A. A. Alandijani , M. M. Tashkandi , G. Y. Bhadila , and A. M. Bagher , “The Effect of Virtual Reality Distraction on Anxiety Level During Dental Treatment Among Anxious Pediatric Patients: A Randomized Clinical Trial,” Journal of Clinical Pediatric Dentistry 47, no. 4 (2023): 63–71. 10.22514/jocpd.2023.036 37408348

[46] N. U. Zitzmann , L. Matthisson , H. Ohla , and T. Joda , “Digital Undergraduate Education in Dentistry: A Systematic Review,” International Journal of Environmental Research and Public Health 17, no. 9 (2020): 3269. 10.3390/ijerph17093269 PMC7246576 32392877

