# Supplementary material for: Augmented and Virtual Reality in Dental Medicine Updated: A Systematic Review and Meta‐Analysis
Source: J Periodontal Res. 2026 Apr 25;61(9):1017–32. doi: 10.1111/jre.70115 (PMC13768981; doi:10.1111/jre.70115)
Supplement: Supplementary file 1 [file JRE-61-1017-s001.docx]

**Supporting information**

**Figure S1.** Meta-analysis and forest plot between VR and TV techniques in relation to heart rate measured in paediatric patients (k = 4).

**Figure S2.** Meta-analysis and forest plot between virtual reality (VR) and Tell–Show–Do (TSD) techniques in relation to heart rate measured in adult patients (k = 6).

**Figure S3.** Meta-analysis and forest plot between VR and Tell-Show-Do techniques in relation to clinicians reported outcomes measured in adults and paediatric patients (k = 7).

**Figure S4.** Meta-analysis and forest plot between VR and TV techniques in relation to clinicians reported outcomes measured in paediatric patients (k = 3).

**Figure S5.** Meta-analysis and forest plot between VR and Tell-Show-Do techniques in relation to patient reported outcomes anxiety levels measured in adult patients (k = 3).

**Figure S6.** Meta-analysis and forest plot between VR and Tell-Show-Do techniques in relation to patient reported outcomes pain levels measured in paediatric patients (k = 7).

**Figure S7.** Meta-analysis and forest plot between VR and Tell-Show-Do techniques in relation to patient reported outcomes anxiety levels measured in paediatric patients -subgrouping analysis by intervention (k = 9).

**Figure S8.** Meta-analysis and forest plot between VR and Tell-Show-Do techniques in relation to patient reported outcomes pain levels measured in adult patients (k = 4).

**Figure S9.** Meta-analysis and forest plot between VR and TV techniques in relation to patient reported outcomes anxiety levels measured in paediatric patients (k = 2).

**Figure S10.** Risk of bias assessment of the non-randomized studies based in ROBINS-I tool.

**Table S1.** Excluded studies after full texts analysis and reasons for exclusion.

| Study | Reason for Exclusion |
| --- | --- |
| Articles | |
| Afrashtehfar et al. (2023) | 2 |
| Afrashtehfar et al. (2021) | 2 |
| Aljabry et al. (2023) | 1 |
| Almashat et al. (2021) | 1 |
| Alsabbagh et al. (2019) | 1 |
| Amini et al. (2021) | 3 |
| Anwar et al. (2024) | 1 |
| Arora et al. (2023) | 1 |
| Arroyo-Cruz et al. (2023) | 1 |
| Bartella et al. (2021) | 3 |
| Bartella et al. (2019) | 1 |
| Bayrak et al. (2020) | 2 |
| Bochet et al. (2024) | 1 |
| Bolding et al. (2022) | 2 |
| Borrelli et al. (2022) | 1 |
| Bouaoud et al. (2021) | 2 |
| Budhathoki et al. (2020) | 2 |
| Bussink et al. (2022) | 1 |
| Caratela et al. (2025) | 1 |
| Carpegna et al. (2023) | 1 |
| Ceccariglia et al. (2022) | 1 |
| Chang et al. (2022) | 1 |
| Chen et al. (2023) | 2 |
| Chen et al. (2020) | 1 |
| Colvenkar et al. (2022) | 1 |
| Condino et al. (2024) | 3 |
| Dixon et al. (2021) | 2 |
| Do et al. (2025) | 1 |
| Dolega-Dolegowski et al. (2022) | 1 |
| Dong et al. (2023) | 1 |
| Dragan et al. (2022) | 1 |
| Ejima et al. (2023) | 1 |
| Elbashti et al. (2020) | 2 |
| Engelschalk et al. (2024) | 1 |
| Fan et al. (2024) | 1 |
| Fan et al. (2024) | 1 |
| Gao et al. (2019) | 1 |
| García Franco et al. (2024) | 1 |
| Geers et al. (2024) | 1 |
| Geers et al. (2024) | 1 |
| Glas et al. (2021) | 1 |
| Han et al. (2022) | 1 |
| Han et al. (2022) | 2 |
| Hu et al. (2022) | 2 |
| Huang et al. (2025) | 1 |
| Jiwan et al. (2021) | 1 |
| Jyoti et al. (2024) | 2 |
| Kaplan et al. (2023) | 1 |
| Karthikeyan et al. (2019) | 1 |
| Karuppiah et al. (2024) | 1 |
| Kawakami et al. (2024) | 1 |
| Kim et al. (2020) | 1 |
| Kim-Berman et al. (2025) | 3 |
| Kim-Berman et al. (2019) | 2 |
| Kostovski et al. (2020) | 1 |
| Koyachi et al. (2021) | 1 |
| Kröplin et al. (2025) | 3 |
| Lamira et al. (2023) | 1 |
| Ledwoń et al. (2024) | 1 |
| Li et al. (2025) | 1 |
| Liebermann et al. (2024) | 5 |
| Lin et al. (2022) | 6 |
| Lin et al. (2024) | 3 |
| Lin et al. (2023) | 1 |
| Liu et al. (2025) | 3 |
| Liu et al. (2021) | 1 |
| Lysenko et al. (2021) | 2 |
| Mai et al. (2024) | 1 |
| Mai et al. (2024) | 2 |
| Manav et al. (2025) | 1 |
| Mangano et al. (2023) | 1 |
| Marchand et al. (2020) | 2 |
| Marhuenda Ramos et al. (2024) | 1 |
| Martinho et al. (2023) | 1 |
| Martinho et al. (2024) | 3 |
| Martinho et al. (2024) | 1 |
| Mladenovic et al. (2022) | 1 |
| Mladenovic et al. (2020) | 1 |
| Mladenovic et al. (2022) | 1 |
| Morales-Vadillo et al. (2019) | 1 |
| Mosch et al. (2025) | 1 |
| Nassief et al. (2024) | 1 |
| Neugarten et al. (2024) | 2 |
| Niloy et al. (2024) | 1 |
| Ochshorn et al. (2025) | 1 |
| Pande et al. (2020) | 2 |
| Parakh et al. (2023) | 1 |
| Pellegrino et al. (2019) | 1 |
| Pepe et al. (2019) | 1 |
| Pham Dang et al. (2021) | 3 |
| Philip et al. (2023) | 1 |
| Pietruski et al. (2019) | 1 |
| Pietruski et al. (2023) | 1 |
| Plaza De Miguel et al. (2023) | 1 |
| Ranauta et al. (2023) | 1 |
| Rantamaa et al. (2022) | 1 |
| Remschmidt et al. (2023) | 1 |
| Riad Deglow et al. (2023) | 1 |
| Rodrigues et al. (2022) | 1 |
| Rodrigues et al. (2023) | 1 |
| Romalee et al. (2024) | 1 |
| Rosa et al. (2025) | 1 |
| Sai Sanjith et al. (2022) | 1 |
| Sasaki et al. (2022) | 1 |
| Schreiber et al. (2021) | 1 |
| Seligman et al. (2023) | 1 |
| Serrano et al. (2023) | 1 |
| Serrano et al. (2020) | 1 |
| Shams et al. (2024) | 2 |
| Shi et al. (2024) | 2 |
| Shi et al. (2022) | 1 |
| Soltanimehr et al. (2019) | 2 |
| Song et al. (2024) | 1 |
| Speidel et al. (2023) | 3 |
| Ströbele et al. (2025) | 1 |
| Ströbele et al. (2023) | 1 |
| Suenaga et al. (2024) | 2 |
| Suenaga et al. (2023) | 2 |
| Sugahara et al. (2021) | 1 |
| Sugahara et al. (2023) | 1 |
| Sukotjo et al. (2023) | 2 |
| Sytek et al. (2021) | 1 |
| Tang et al. (2025) | 2 |
| Tang et al. (2021) | 3 |
| Touati et al. (2019) | 1 |
| Towers et al. (2022) | 1 |
| Tricio et al. (2022) | 1 |
| Uppal et al. (2022) | 1 |
| Urbankova et al. (2022) | 1 |
| Vannaprathip et al. (2025) | 1 |
| Wan et al. (2023) | 3 |
| Wan et al. (2024) | 3 |
| Wan et al. (2024) | 3 |
| Wang et al. (2021) | 1 |
| Wang et al. (2024) | 1 |
| Wilkat et al. (2024) | 3 |
| Wu et al. (2023) | 6 |
| Xue et al. (2024) | 1 |
| Yamashita et al. (2023) | 1 |
| Yao et al. (2020) | 3 |
| Yari et al. (2024) | 1 |
| Yaru Wei et al. (2024) | 1 |
| Zaragoza-Siqueiros et al. (2019) | 3 |
| Zhang et al. (2025) | 1 |
| Zhou et al. (2019) | 1 |
| Zorzal et al. (2021) | 1 |

| Trials | |
| --- | --- |
| Australian New Zealand Clinical Trials Registry (2021) | 6 |
| Australian New Zealand Clinical Trials Registry (2022) | 6 |
| Australian New Zealand Clinical Trials Registry (2024) | 6 |
| Iranian Registry of Clinical Trials (2020) | 6 |
| Iranian Registry of Clinical Trials (2022) | 6 |
| Iranian Registry of Clinical Trials (2023) | 6 |
| National Clinical Trial (2025) | 6 |
| National Clinical Trial (2025) | 6 |
| National Clinical Trial (2024) | 6 |
| National Clinical Trial (2024) | 6 |
| National Clinical Trial (2024) | 6 |
| National Clinical Trial (2024) | 6 |
| National Clinical Trial (2025) | 6 |
| National Clinical Trial (2025) | 6 |
| National Clinical Trial (202 2) | 6 |
| National Clinical Trial (2019) | 6 |
| National Clinical Trial (2019) | 6 |
| National Clinical Trial (2020) | 6 |
| National Clinical Trial (2020) | 6 |
| National Clinical Trial (2021) | 6 |
| National Clinical Trial (2021) | 6 |
| National Clinical Trial (2022) | 6 |
| National Clinical Trial (2023) | 6 |
| National Clinical Trial (2024) | 6 |
| National Clinical Trial (2023) | 6 |
| National Clinical Trial (2023) | 6 |
| National Clinical Trial (2020) | 6 |
| National Clinical Trial (2023) | 6 |
| National Clinical Trial (2021) | 6 |
| National Clinical Trial (2024) | 6 |
| Pan African Clinical Trials Registry (2022) | 6 |

## Reasons for Exclusion

1. Inadequate study type (animal studies, in-vitro investigations, case reports, abstract, case series with less than 15 participants etc.)

2. Insufficient information on defined AR/MR/VR-technology used

3. Studies not related to dentistry (exclude maxillofacial surgery not directly related to teeth or implants) or dental education (educational studies not evaluating dentists or dental students)

4. Articles not reported in English

5. Multiple publications on the same population

6. Trial registered, not yet published

# References

1. Afrashtehfar, K. I., Al-Sammarraie, A., Yang, J. W., Chen, H. & Saeed, M. H. Pre-clinical undergraduate students' perspectives on the adoption of virtual and augmented reality to their dental learning experience: A one-group pre- and post-test design protocol (2023)

2. Afrashtehfar, K. I., Yang, J. W., Al-Sammarraie, A., Chen, H. & Saeed, M. H. Pre-clinical undergraduate students' perspectives on the adoption of virtual and augmented reality to their dental learning experience: A one-group pre- and post-test design protocol (2021)

3. Aljabry, M. A., Alkadi, K. & Awawdeh, M. The impact of technology on patient knowledge acquisition and retention of oral hygiene instructions and associated risks (2023)

4. Almashat, R., Baakdah, R., Justinia, T., Ashour, F., AlBishi, N., Almotheby, F., Algafel, K., Alshahrani, A. & Alalowi, B. Paediatric dentists’ perception on the effect of virtual reality on patients (2021)

5. Alsabbagh, A. Y., Watt-Smith, S. & Leeson, R. Comparison of CT, 3D Reconstruction, and Virtual Reality in the Interpretation of Maxillofacial Fractures (2019)

6. Amini, H., Gregory, M. E., Abrams, M. A., Luna, J., Roland, M., Sova, L. N., Bean, C., Huang, Y., Pfeil, S. A., Townsend, J. & Lin, E. D. Feasibility and usability study of a pilot immersive virtual reality-based empathy training for dental providers (2021)

7. Anwar, M., Shaikh, T., Tahir, A., Falaq, R., Khalil, O. & Shafiq, A. Effectiveness of virtual reality (vr) to enhance the training and education of dental students, focusing on skill acquisition and retention (2024)

8. Arora, O., Sivaswamy, V., Ahmed, N. & Ganapathy, D. Effectiveness of digital visualization in teaching crown preparation to predoctoral dental students – A Pilot study (2023)

9. Arroyo-Cruz, G., Orozco-Varo, A., Domínguez-Cardoso, P., Árbol-Carrero, A. B. & Jiménez-Castellanos, E. FitJaw Mobile, a virtual reality device applied to dentistry: An analysis based on two patient treatments (2023)

10. Bartella, A. K., Kamal, M., Kuhnt, T., Hering, K., Halama, D., Pausch, N. C. & Lethaus, B. Mixed reality in oral and maxillofacial surgery: a symbiosis of virtual and augmented reality or a pointless technological gadget? (2021)

11. Bartella, A. K., Kamal, M., Scholl, I., Schiffer, S., Steegmann, J., Ketelsen, D., Hölzle, F. & Lethaus, B. Virtual reality in preoperative imaging in maxillofacial surgery: implementation of "the next level"? (2019)

12. Bayrak, M., Alsadoon, A., Prasad, P. W. C., Venkata, H. S., Ali, R. S. & Haddad, S. A novel rotation invariant and Manhattan metric-based pose refinement: Augmented reality-based oral and maxillofacial surgery (2020)

13. Bochet, Q., Raoul, G., Lauwers, L. & Nicot, R. Augmented reality in implantology: virtual surgical checklist and augmented implant placement (2024)

14. Bolding, S. L. & Reebye, U. N. Accuracy of haptic robotic guidance of dental implant surgery for completely edentulous arches (2022)

15. Borrelli, B., Endrighi, R., Jurasic, M. M., Hernandez, H., Jones, E., Ospina, J., Cabral, H. J., Quintiliani, L. M. & Werntz, S. A smoking cessation induction intervention via virtual reality headset during a dental cleaning: protocol for a randomized controlled trial (2022)

16. Bouaoud, J., El Beheiry, M., Jablon, E., Schouman, T., Bertolus, C., Picard, A., Masson, J. B. & Khonsari, R. H. DIVA, a 3D virtual reality platform, improves undergraduate craniofacial trauma education (2021)

17. Budhathoki, S., Alsadoon, A., Prasad, P. W. C., Haddad, S. & Maag, A. Augmented reality for narrow area navigation in jaw surgery: Modified tracking by detection volume subtraction algorithm (2020)

18. Bussink, T., Maal, T., Meulstee, J. & Xi, T. Augmented reality guided condylectomy (2022)

19. Caratela, N., Shykhon, M., Milward, M. & Yonel, Z. The use of 360-videos to bridge the gap between pre-clinical and clinical dental teaching (2025)

20. Carpegna, G., Scotti, N., Alovisi, M., Comba, A., Berutti, E. & Pasqualini, D. Endodontic microsurgery virtual reality simulation and digital workflow process in a teaching environment (2023)

21. Ceccariglia, F., Cercenelli, L., Badiali, G., Marcelli, E. & Tarsitano, A. Application of Augmented Reality to Maxillary Resections: A Three-Dimensional Approach to Maxillofacial Oncologic Surgery (2022)

22. Chang, A. H., Lin, P. C., Lin, P. C., Lin, Y. C., Kabasawa, Y., Lin, C. Y. & Huang, H. L. Effectiveness of Virtual Reality-Based Training on Oral Healthcare for Disabled Elderly Persons: A Randomized Controlled Trial (2022)

23. Chen, D., Liu, X., Liu, Y., Wang, X., Zheng, J. & Wu, L. Virtual reality used in undergraduate orthodontic education (2023)

24. Chen, G., Zeng, W., Yin, H., Yu, Y., Ju, R. & Tang, W. The Preliminary Application of Augmented Reality in Unilateral Orbitozygomatic Maxillary Complex Fractures Treatment (2020)

25. Colvenkar, S. & Ali, M. M. Management of gag reflex with a virtual reality headset (2022)

26. Condino, S., Cutolo, F., Carbone, M., Cercenelli, L., Badiali, G., Montemurro, N. & Ferrari, V. Registration Sanity Check for AR-guided Surgical Interventions: Experience from Head and Face Surgery (2024)

27. Dixon, J., Towers, A., Martin, N. & Field, J. Re-defining the virtual reality dental simulator: Demonstrating concurrent validity of clinically relevant assessment and feedback (2021)

28. Do, N. M., Manière, M. C., Lefebvre, F. & Clauss, F. Efficacy of virtual reality hypnosis versus conscious sedation with nitrous oxide in the management of dental anxiety in paediatric dentistry: protocol for a prospective randomized controlled trial (2025)

29. Dolega-Dolegowski, D., Proniewska, K., Dolega-Dolegowska, M., Pregowska, A., Hajto-Bryk, J., Trojak, M., Chmiel, J., Walecki, P. & Fudalej, P. S. Application of holography and augmented reality-based technology to visualize the internal structure of the dental root - a proof of concept (2022)

30. Dong, S., Du, C., He, B., Zhu, Z., Han, D., Jin, W., Lin, H. & Shi, B. Application of an effective marker-less augmented reality image guidance method in dental implant surgery (2023)

31. Dragan, I. F., Hamza, T. & Bhadila, G. Integrating a mixed reality platform in the diagnosis and treatment planning of periodontal conditions for adults and paediatric patients (2022)

32. Ejima, K. I., Sasaki, T., Honda, K. & Arai, Y. Development of practical education system for intraoral radiography using Extended Reality (2023)

33. Elbashti, M. E., Itamiya, T., Aswehlee, A. M., Sumita, Y. I., Ella, B. & Naveau, A. Augmented Reality for Interactive Visualization of 3D Maxillofacial Prosthetic Data (2020)

34. Engelschalk, M., Al Hamad, K. Q., Mangano, R., Smeets, R. & Molnar, T. F. Dental implant placement with immersive technologies: A preliminary clinical report of augmented and mixed reality applications (2024)

35. Fan, X., Feng, Y., Tao, B., Shen, Y., Wu, Y. & Chen, X. A hybrid robotic system for zygomatic implant placement based on mixed reality navigation (2024)

36. Fan, X., Feng, Y., Tao, B., Shen, Y., Wu, Y. & Chen, X. A hybrid robotic system for zygomatic implant placement based on mixed reality navigation (2024)

37. Gao, Y., Lin, L., Chai, G. & Xie, L. A feasibility study of a new method to enhance the augmented reality navigation effect in mandibular angle split osteotomy (2019)

38. García Franco, C., Riad Deglow, E., Montero, J., Abella Sans, F., Nagendrababu, V., Dummer, P. M. H., Lobo Galindo, A. B. & Zubizarreta-Macho, Á. Endodontic access with different computer navigation systems in calcified root canals (2024)

39. Geers, A. L., Seligman, L. D., Pituch, K. A., Colagiuri, B., Marusak, H. A., Rabinak, C. A., Al-Ado, S. L., Turner, N. & Nedley, M. A test of pre-exposure spacing and multiple context pre-exposure on the mechanisms of latent inhibition of dental fear: A study protocol (2024)

40. Geers, A. L., Seligman, L. D., Pituch, K. A., Colagiuri, B., Marusak, H. A., Rabinak, C. A., Turner, N., Al-Ado, S. L. & Nedley, M. A study protocol testing pre-exposure dose and compound pre-exposure on the mechanisms of latent inhibition of dental fear (2024)

41. Glas, H. H., Kraeima, J., van Ooijen, P. M. A., Spijkervet, F. K. L., Yu, L. & Witjes, M. J. H. Augmented Reality Visualization for Image-Guided Surgery: A Validation Study Using a Three-Dimensional Printed Phantom (2021)

42. Han, B., Li, R., Huang, T., Ma, L., Liang, H., Zhang, X. & Liao, H. An accurate 3D augmented reality navigation system with enhanced autostereoscopic display for oral and maxillofacial surgery (2022)

43. Han, J. J., Sodnom-Ish, B., Eo, M. Y., Kim, Y. J., Oh, J. H., Yang, H. J. & Kim, S. M. Accurate Mandible Reconstruction by Mixed Reality, 3D Printing, and Robotic-Assisted Navigation Integration (2022)

44. Hu, S. & Lai, B. W. P. Increasing empathy for children in dental students using virtual reality (2022)

45. Huang, Y. T., Addab, S., Bertolizio, G., Hamdy, R., Thorstad, K. & Tsimicalis, A. Use of Virtual Reality in the Paediatric Perioperative Setting and for Induction of Anaesthesia: Mixed Methods Pilot Feasibility Study (2025)

46. Jiwan, N., Ark, A., Middup, R., Rasaiah, S., Davies, R., Hyde, T. P. & Keeling, A. A Comparative study on the Effectiveness of Augmented Reality on Denture Tooth Selection (2021)

47. Jyoti, D., Shams, S. A., Anand, P., Sagar, S., Raj, N. & Singh, S. An In Vivo Study to Evaluate and Compare Anxiety and Behaviour Management of Paediatric Patients Using Distraction Techniques (2024)

48. Kaplan, N., Yang, K., Zhao, L., Purnell, C., Patel, P. & Alkureishi, L. Virtual Reality Surgical Planning for Free Fibula Mandibular Reconstructions (2023)

49. Karthikeyan, G. & Bennis, M. A. Effect of visual reality on the operators experience during local anesthetic administration for paediatric patients (2019)

50. Karuppiah, M., Balamurugan, S. R., Rajashekaran, S., Chowdhary, N., Vundala, R. R. & Shaji, N. E. Evaluation of Effect of Distraction Techniques Using Virtual Reality and Eight-dimension Audio Analgesia Methods on Pain Perception and Anxiety Levels in Children During Restorative Procedures: a Comparative In Vivo Study (2024)

51. Kawakami, H., Suenaga, H., Sakakibara, A. & Hoshi, K. Computer-assisted surgery with markerless augmented reality for the surgical removal of mandibular odontogenic cysts: report of two clinical cases (2024)

52. Kim, S. H., Lee, S. J., Choi, M. H., Yang, H. J., Kim, J. E., Huh, K. H., Lee, S. S., Heo, M. S., Hwang, S. J. & Yi, W. J. Quantitative Augmented Reality-Assisted Free-Hand Orthognathic Surgery Using Electromagnetic Tracking and Skin-Attached Dynamic Reference (2020)

53. Kim-Berman, H., Herremans, J., Bonine, B., Aronovich, S. & Ramaswamy, V. Accuracy and user experience of dental diagnosis of a patient with cleidocranial dysplasia using immersive virtual reality and cone-beam computed tomography multiplanar reconstructions (2025)

54. Kim-Berman, H., Karl, E., Sherbel, J., Sytek, L. & Ramaswamy, V. Validity and User Experience in an Augmented Reality Virtual Tooth Identification Test (2019)

55. Kostovski, M., Mazhenkovska, L. K., Petrova, I. K. & Mitevska, E. Evaluation of dentistry students' perceptions about implementing virtual reality in undergraduate histology education (2020)

56. Koyachi, M., Sugahara, K., Odaka, K., Matsunaga, S., Abe, S., Sugimoto, M. & Katakura, A. Accuracy of Le Fort I osteotomy with combined computer-aided design/computer-aided manufacturing technology and mixed reality (2021)

57. Kröplin, J., Friedrich, C., Harms, L., Lenz, J. H. & Frerich, B. A virtual reality training program to enhance surgical skills in dental education: Prospective comparative analysis of students and residents (2025)

58. Lamira, J. M., Wilson, C. S., Leppek, N. C., Orr, C. M., De la Rosa, L. M. & Greany, T. J. A pilot study of local anaesthesia training using a mixed-reality haptic fidelity model (2023)

59. Ledwoń, A., Dębski, P., Jędrusik, P., Mielcarska, S., Misiolek, H., Meisner, M., Łopacińska, M. & Skucha-Nowak, M. An Attempt to Use Virtual Reality as a Tool to Reduce Patient Anxiety During Dental Treatment (2024)

60. Li, Y., Chen, X., Huang, Y. D., He, Q., Li, D., Hu, S., Xu, P., Chen, T. & Ran, X. Comparing between virtual reality based pre-clinical implantation training and traditional learning methods (2025)

61. Liebermann, A., Bjelopavlovic, M., Rauch, A., Schlenz, M. A. & Erdelt, K. Assessment of a virtual prosthetic case planning environment for dental education - A multicentric analysis (2024)

62. Lin, L., Liu, X., Gao, Y., Aung, Z. M., Xu, H., Wang, B., Xie, L., Yang, X. & Chai, G. The application of augmented reality in craniofacial bone fracture reduction: study protocol for a randomized controlled trial (2022)

63. Lin, P. C., Wung, S. F., Lin, P. C., Lin, Y. C., Lin, C. Y. & Huang, H. L. Virtual reality-based simulation learning on geriatric oral health care for nursing students: a pilot study (2024)

64. Lin, S., Hu, L. H., Zhang, W. B., Lin, Y., Di, P. & Peng, X. Single-stage computer-assisted approach for tumour resection and functional restoration of Brown class III maxillary defects—titanium mesh and zygomatic implants combined with vascularized anterolateral thigh flap (2023)

65. Liu, H., Zhu, Z., Huang, L., Liu, L., Liu, Y., Liu, X., Wu, C. & Luo, E. Virtual Reality in Preclinical Orthognathic Surgery Education for Dental Students (2025)

66. Liu, K., Gao, Y., Abdelrehem, A., Zhang, L., Chen, X., Xie, L. & Wang, X. Augmented reality navigation method for recontouring surgery of craniofacial fibrous dysplasia (2021)

67. Lysenko, A. V., Yaremenko, A. I., Ivanov, V. M., Strelkov, S. V. & Ivanova, E. A. Experience in the use of a radio-oriented augmented reality navigation system for biopsy of a neoplasm of the lower jaw (2021)

68. Mai, H. N., Ngo, H. C., Cho, S. H. & Lee, D. H. Automated scoring and augmented reality visualization software program for evaluating tooth preparations (2024)

69. Mai, H. N., Ngo, H. C., Cho, S. H. & Lee, D. H. Automated scoring and augmented reality visualization software program for evaluating tooth preparations (2024)

70. Manav, E. Y., Akbiyik, S. Y., Ceylan, A. B., Çakiroğlu, A. E. Y. & Tuncer, D. Effects of virtual reality and layered tooth model training on manual dexterity in preclinical dental education (2025)

71. Mangano, F. G., Admakin, O., Lerner, H. & Mangano, C. Artificial intelligence and augmented reality for guided implant surgery planning: A proof of concept (2023)

72. Marchand, L., Touati, R., Fehmer, V., Ducret, M. & Sailer, I. Latest advances in augmented reality technology and its integration into the digital workflow (2020)

73. Marhuenda Ramos, M. T., Faus-Matoses, I., Zubizarreta-Macho, Á, Riad Deglow, E., Lobo Galindo, A. B., Abella Sans, F., Belanche Monterde, A. & Faus Matoses, V. Influence of augmented reality technique on the accuracy of autotransplanted teeth in surgically created sockets (2024)

74. Martinho, F. C., Griffin, I. L., Price, J. B. & Tordik, P. A. Augmented Reality and 3-dimensional Dynamic Navigation System Integration for Osteotomy and Root-end Resection (2023)

75. Martinho, F. C., Qadir, S. J., Griffin, I. L., Melo, M. A. S. & Fay, G. G. Augmented Reality Head-Mounted Device and Dynamic Navigation System for Postremoval in Maxillary Molars (2024)

76. Martinho, F. C., Qadir, S. J., Griffin, I. L., Melo, M. A. & Fay, G. G. Augmented Reality Head-Mounted Device and Dynamic Navigation System for Post-Removal in Maxillary Molars (2024)

77. Mladenovic, R., AlQahtani, S., Mladenovic, K., Bukumiric, Z. & Zafar, S. Effectiveness of technology-enhanced teaching methods of undergraduate dental skills for local anaesthesia administration during COVID-19 era: students' perception (2022)

78. Mladenovic, R., Dakovic, D., Pereira, L., Matvijenko, V. & Mladenovic, K. Effect of augmented reality simulation on administration of local anaesthesia in paediatric patients (2020)

79. Mladenovic, R., Matvijenko, V., Subaric, L. & Mladenovic, K. Augmented reality as e-learning tool for intraoral examination and dental charting during COVID-19 era (2022)

80. Morales-Vadillo, R., Guevara-Canales, J. O., Flores-Luján, V. C., Robello-Malatto, J. M., Bazán-Asencios, R. H. & Cava-Vergiú, C. E. Use of virtual reality as a learning environment in dentistry (2019)

81. Mosch, R., Alevizakos, V., Ströbele, D. A., Schiller, M. & von See, C. Exploring Augmented Reality for Dental Implant Surgery: Feasibility of Using Smartphones as Navigation Tools (2025)

82. Nassief, S., Al Ali, H., Towers, A., Field, J. & Martin, N. Dental students' perceptions of the use of two-dimensional and three-dimensional vision in dental education using a three-dimensional haptic simulator: A qualitative study (2024)

83. Neugarten, J. M. Accuracy and Precision of Haptic Robotic-Guided Implant Surgery in a Large Consecutive Series (2024)

84. Niloy, I., Liu, R. H., Pham, N. M. & Yim, C. M. R. Novel Use of Virtual Reality and Augmented Reality in Temporomandibular Total Joint Replacement Using Stock Prosthesis (2024)

85. Ochshorn, J., Daly, K. A., Zaninovic, V., Heyman, R. E., Smith Slep, A. M. & Wolff, M. S. Examining barriers and facilitators of dental fear treatment adoption: A qualitative study of practicing dentists (2025)

86. Pande, P., Rana, V., Srivastava, N. & Kaushik, N. Effectiveness of different behaviour guidance techniques in managing children with negative behaviour in a dental setting: A randomized control study (2020)

87. Parakh, H. & Thosar, N. Evaluation and comparison of the effectiveness of kaleidoscope and virtual reality goggles to reduce dental anxiety in young children undergoing administration of local anaesthesia (2023)

88. Pellegrino, G., Mangano, C., Mangano, R., Ferri, A., Taraschi, V. & Marchetti, C. Augmented reality for dental implantology: a pilot clinical report of two cases (2019)

89. Pepe, A., Trotta, G. F., Mohr-Ziak, P., Gsaxner, C., Wallner, J., Bevilacqua, V. & Egger, J. A Marker-Less Registration Approach for Mixed Reality-Aided Maxillofacial Surgery: A Pilot Evaluation (2019)

90. Pham Dang, N., Chandelon, K., Barthélémy, I., Devoize, L. & Bartoli, A. A proof-of-concept augmented reality system in oral and maxillofacial surgery (2021)

91. Philip, N., Ali, K., Duggal, M., Daas, H. & Nazzal, H. Effectiveness and Student Perceptions of Haptic Virtual Reality Simulation Training as an Instructional Tool in Pre-Clinical Paediatric Dentistry: A Pilot Pedagogical Study (2023)

92. Pietruski, P., Majak, M., Światek-Najwer, E., Żuk, M., Popek, M., Mazurek, M., Świecka, M. & Jaworowski, J. Supporting mandibular resection with intraoperative navigation utilizing augmented reality technology – A proof of concept study (2019)

93. Pietruski, P., Majak, M., Świątek-Najwer, E., Żuk, M., Popek, M., Świecka, M., Jaworowski, J. & Mazurek, M. Replacing cutting guides with an augmented reality-based navigation system: A feasibility study in the maxillofacial region (2023)

94. Plaza De Miguel, C., Sánchez-Margallo, J. A., Sánchez-Margallo, J. A., Moreno Vázquez, J. C. & Sánchez-Margallo, F. M. Use of 3D printed markers for Mixed Reality-guided maxillofacial surgery (2023)

95. Ranauta, A., Audsley, B. & Coulthard, P. The integration of haptic training into the QMUL dental curriculum (2023)

96. Rantamaa, H. R., Kangas, J., Jordan, M., Mehtonen, H., Mäkelä, J., Ronkainen, K., Turunen, M., Sundqvist, O., Syrjä, I., Järnstedt, J. & Raisamo, R. Evaluation of voice commands for mode change in virtual reality implant planning procedure (2022)

97. Remschmidt, B., Rieder, M., Gsaxner, C., Gaessler, J., Payer, M. & Wallner, J. Augmented Reality-Guided Apicoectomy Based on Maxillofacial CBCT Scans (2023)

98. Riad Deglow, E., Zubizarreta-Macho, Á, González Menéndez, H., Lorrio Castro, J., Galparsoro Catalán, A., Tzironi, G., Lobo Galindo, A. B., Alonso Ezpeleta, LÓ & Hernández Montero, S. Comparative analysis of two navigation techniques based on augmented reality technology for the orthodontic mini-implants placement (2023)

99. Rodrigues, P., Esteves, A., Botelho, J., Machado, V., Zagalo, C., Zorzal, E. R., Mendes, J. J. & Lopes, D. S. Usability, acceptance, and educational usefulness study of a new haptic operative dentistry virtual reality simulator (2022)

100. Rodrigues, P., Nicolau, F., Norte, M., Zorzal, E., Botelho, J., Machado, V., Proença, L., Alves, R., Zagalo, C., Lopes, D. S. & Mendes, J. J. Preclinical dental students’ self-assessment of an improved operative dentistry virtual reality simulator with haptic feedback (2023)

101. Romalee, W., Tsai, F. T., Hsu, Y. C., Hsu, M. L. & Wang, D. H. Effectiveness of mobile augmented reality-integrated oral health education for community-dwelling older adults: A randomized controlled trial (2024)

102. Rosa, A., De Angelis, R., Pujia, A. M., Cardelli, P. & Arcuri, C. Virtual reality in specialized dentistry: employing virtual reality for the alleviation of pain and anxiety in hereditary angioedema patients (2025)

103. Sai Sanjith, A. & Sadeep, H. Evaluation of knowledge, attitude and practice of dental educators on virtual reality-based technology in dental education (2022)

104. Sasaki, T., Dehari, H., Ogi, K. & Miyazaki, A. Application of a mixed reality device to oral surgery (2022)

105. Schreiber, S., Sukotjo, C., Yuan, J. C. C. & Santoso, M. Virtual reality program of dental implant surgery: Student perception (2021)

106. Seligman, L. D., Geers, A. L., Kramer, L., Clemens, K. S., Pituch, K. A., Colagiuri, B., Marusak, H. A., Rabinak, C. A., Turner, N. & Nedley, M. Study protocol of an investigation of attention and prediction error as mechanisms of action for latent inhibition of dental fear in humans (2023)

107. Serrano, C. M., Bakker, D. R., Zamani, M., de Boer, I. R., Koopman, P., Wesselink, P. R., Berkhout, E. & Vervoorn, J. M. Virtual reality and haptics in dental education: Implementation progress and lessons learned after a decade (2023)

108. Serrano, C. M., Wesselink, P. R. & Vervoorn, J. M. First experiences with patient-centered training in virtual reality (2020)

109. Shams, S. A., Reddy, A., Vaghela, L. L., Jain, M., Naik, H. & Krishnan, P. Comparison of Effectiveness of Audio and VR Distraction Techniques in Managing Paediatric Dental Patients (2024)

110. Shi, J. Y., Liu, B. L., Wu, X. Y., Liu, M., Zhang, Q., Lai, H. C. & Tonetti, M. S. Improved positional accuracy of dental implant placement using a haptic and machine-vision-controlled collaborative surgery robot: A pilot randomized controlled trial (2024)

111. Shi, J., Liu, S., Zhu, Z., Deng, Z., Bian, G. & He, B. Augmented reality for oral and maxillofacial surgery: The feasibility of a marker-free registration method (2022)

112. Soltanimehr, E., Bahrampour, E., Imani, M. M., Rahimi, F., Almasi, B. & Moattari, M. Effect of virtual versus traditional education on theoretical knowledge and reporting skills of dental students in radiographic interpretation of bony lesions of the jaw (2019)

113. Song, S. J., Tang, M., Gwartzman, B., Lee, D., Romandini, P., Salem, M., Kwon, P., Feiner, S. K. & Sailer, I. Augmented-reality-assisted intraoral scanning: A proof-of-concept study (2024)

114. Speidel, R., Schneider, A., Walter, S., Grab-Kroll, C. & Oechsner, W. Immersive medium for early clinical exposure - knowledge acquisition, spatial orientation and the unexpected role of annotation in 360° VR photos (2023)

115. Ströbele, D. A., Othman, A., Heiermann, K., Samih, H. M. & von See, C. Experienced versus unexperienced dentists in bracket placement using an Augmented Reality (AR) conventional mobile devices (2025)

116. Ströbele, D. A., Othman, A., Meier, M. A., Aboulazm, K. & von See, C. Augmented reality in orthodontics for bracket placement using conventional mobile devices: Technical note (2023)

117. Suenaga, H., Sakakibara, A., Koyama, J. & Hoshi, K. A clinical presentation of markerless augmented reality assisted surgery for resection of a dentigerous cyst in the maxillary sinus (2024)

118. Suenaga, H., Sakakibara, A., Taniguchi, A. & Hoshi, K. Computer-Assisted Preoperative Simulation and Augmented Reality for Extraction of Impacted Supernumerary Teeth: A Clinical Case Report of Two Cases (2023)

119. Sugahara, K., Koyachi, M., Koyama, Y., Sugimoto, M., Matsunaga, S., Odaka, K., Abe, S. & Katakura, A. Mixed reality and three-dimensional printed models for resection of maxillary tumor: A case report (2021)

120. Sugahara, K., Koyachi, M., Tachizawa, K., Iwasaki, A., Matsunaga, S., Odaka, K., Sugimoto, M., Abe, S., Nishii, Y. & Katakura, A. Using mixed reality and CAD/CAM technology for treatment of maxillary non-union after Le Fort I osteotomy: a case description (2023)

121. Sukotjo, C., Bertucci, D. E., Patel, J. Y., Yuan, Jc- C. & Santoso, M. Evaluating augmented reality e-typodont to improve a patient's dental implant health literacy (2023)

122. Sytek, L., Inglehart, M. R., Ramaswamy, V., Aronovich, S., Edwards, S. & Kim-Berman, H. Comparisons of orthodontic residents' performance and attitudes using 2D, 3D, and virtual reality surgical simulation methods (2021)

123. Tang, T., Li, X., Lin, Y. & Liu, C. Comparing digital real-time versus virtual simulation systems in dental education for preclinical tooth preparation of molars for metal-ceramic crowns (2025)

124. Tang, Z. N., Hu, L. H., Soh, H. Y., Yu, Y., Zhang, W. B. & Peng, X. Accuracy of Mixed Reality Combined with Surgical Navigation Assisted Oral and Maxillofacial Tumor Resection (2021)

125. Touati, R., Richert, R., Millet, C., Farges, J. C., Sailer, I. & Ducret, M. Comparison of Two Innovative Strategies Using Augmented Reality for Communication in Aesthetic Dentistry: A Pilot Study (2019)

126. Towers, A., Dixon, J., Field, J., Martin, R. & Martin, N. Combining virtual reality and 3D-printed models to simulate patient-specific dental operative procedures-A study exploring student perceptions (2022)

127. Tricio, J. A., Kleiman, S. E., Eiriksson, V. I., Vicuña, D. P., Cacciuttolo, F. R., Jorquera, G. A., Córdova, C. G., Gualda, J. I., Gutiérrez, M. F., Villalón, P. A. & Orsini, C. A. Students' and tutors' perceptions of a deliberate simulated practice using patient-specific virtual and three-dimensional printed teeth models: A pilot study (2022)

128. Uppal, S. & Chegini, S. 63. A qualitative evaluation of the perspectives of oral surgeons and dentists with special interests on the clinical applications of Augmented Reality in oral surgery (2022)

129. Urbankova, A., Palomo, L. & Engebretson, S. P. A complex haptic exercise to predict pre-clinic operative dentistry performance: A prospective study (2022)

130. Vannaprathip, N., Haddawy, P., Schultheis, H. & Suebnukarn, S. SDMentor: A virtual reality-based intelligent tutoring system for surgical decision making in dentistry (2025)

131. Wan, T., Liu, K., Li, B. & Wang, X. Validity of an immersive virtual reality training system for orthognathic surgical education (2023)

132. Wan, T., Liu, K., Li, B. & Wang, X. Effectiveness of immersive virtual reality in orthognathic surgical education: A randomized controlled trial (2024)

133. Wan, T., Liu, K., Li, B. & Wang, X. Effectiveness of immersive virtual reality in orthognathic surgical education: A randomized controlled trial (2024)

134. Wang, C., Peng, C., Hou, Y. & Chen, M. Augmented Reality Research of Measuring X-Ray Dental Film Alveolar Bone Based on Computer Image Analysis System (2021)

135. Wang, X., Shujaat, S., Shaheen, E. & Jacobs, R. Quality and haptic feedback of three-dimensionally printed models for simulating dental implant surgery (2024)

136. Wilkat, M., Schrader, F., Trusch, J., Karnatz, N., Becker, K., Saigo, L. & Rana, M. Enhancing surgical occlusion setting in orthognathic surgery planning using mixed reality technology: a comparative study (2024)

137. Wu, W., Le May, S., Hung, N., Fortin, O., Genest, C., Francoeur, M., Guingo, E., St-Arneault, K., Sylfra, A., Vu, A. K. & et al., Effects of a Virtual Reality Game on Children's Anxiety During Dental Procedures (VR-TOOTH): protocol for a Pilot Randomized Controlled Trial (2023)

138. Xue, F., Zhang, R., Dai, J., Zhang, Y. & Luan, Q. X. Clinical application of mixed reality holographic imaging technology in scaling and root planing of severe periodontitis: a proof of concept (2024)

139. Yamashita, Y., Aijima, R. & Danjo, A. Clinical effects of different virtual reality presentation content on anxiety and pain: a randomized controlled trial (2023)

140. Yao, J., Zeng, W., Zhou, S., Cheng, J., Huang, C. & Tang, W. Augmented Reality Technology Could Be an Alternative Method to Treat Craniomaxillofacial Foreign Bodies: A Comparative Study Between Augmented Reality Technology and Navigation Technology (2020)

141. Yari, A., Fasih, P., Goodarzi, A., Nouralishahi, A. & Nikeghbal, D. The effect of augmented reality book on the proficiency of local anaesthesia administration of the inferior alveolar nerve (2024)

142. Yaru Wei, Zhengjun Peng. Application of Simodont virtual simulation system for preclinical teaching of access and coronal cavity preparation (2024)

143. Zaragoza-Siqueiros, J., Medellin-Castillo, H. I., de la Garza-Camargo, H., Lim, T. & Ritchie, J. M. An integrated haptic-enabled virtual reality system for orthognathic surgery planning (2019)

144. Zhang, Y., Li, X., Wei, X., Xiao, J., Zhang, Q. & Mai, S. A pilot study on the application of a digital dental teaching evaluation system in Class II cavity preparation teaching (2025)

145. Zhou, Y., Yoo, P., Feng, Y., Sankar, A., Sadr, A. & Seibel, E. J. Towards AR-assisted visualisation and guidance for imaging of dental decay (2019)

146. Zorzal, E. R., Paulo, S. F., Rodrigues, P., Mendes, J. J. & Lopes, D. S. An immersive educational tool for dental implant placement: A study on user acceptance (2021)

## References for Excluded Trial Registrations

1. Australian New Zealand Clinical Trials Registry. Efficacy of virtual reality and anaglyph 3D glasses on children's behaviour (2021)

2. Australian New Zealand Clinical Trials Registry. Effect of virtual reality and music on anxiety and perioperative pain in impacted third molar extraction under local anaesthesia (2022)

3. Australian New Zealand Clinical Trials Registry. Evaluating the effect of virtual reality distraction techniques on pain and anxiety in paediatric dental patients during local anaesthesia administration (2024)

4. Iranian Registry of Clinical Trials. Estimation of the Effects of Virtual Reality on Reducing Pain and Anxiety in Paediatric Dentistry (2020)

5. Iranian Registry of Clinical Trials. Application vs virtual reality effect on kids fear & anxiety (2022)

6. Iranian Registry of Clinical Trials. The effect of Virtual reality headsets on reducing pain and anxiety in implant (2023)

7. National Clinical Trial. The Use of Virtual Reality Glasses to Reduce Anxiety in Fixed Orthodontic Treatments (2025)

8. National Clinical Trial. Evaluation of the Effect of Relaxation Exercise and Virtual Reality Application on Pain, Trismus, Sleep, Kinesiophobia and Anxiety Level After Impacted Third Molar Dental Surgery (2025)

9. National Clinical Trial. Effectiveness of Metaverse-Based Immersive Technology Intervention for Oral Health Education: A Quasi-Experimental Study (2024)

10. National Clinical Trial. Investigating the Impact of Virtual Reality Glasses on Sedation Requirement and Patient Experience in Patients Undergoing Oral Surgery (2024)

11. National Clinical Trial. Effect of Active and Passive Augmented Reality Glasses in Behavioral Children Management for Inferior Alveolar Nerve Block - A Randomized Controlled Trial (2024)

12. National Clinical Trial. The Feasibility and Clinical Utility of the Use of Virtual Reality for the Management of Paediatric Dental Anxiety (2024)

13. National Clinical Trial. Effects of Virtual Reality Relaxation on the Anxiety Levels of Adolescents and Adults During Orthodontic Bonding: A Randomized Controlled Trial (2025)

14. National Clinical Trial. The Effects of Virtual Reality and External Cooling and Vibration on Dental Anxiety and Pain During Inferior Alveolar Nerve Block in Children: a Randomized Clinical Trial (2025)

15. National Clinical Trial. Virtual Reality Distraction and Dental Anxiety Among Anxious Paediatric Patients (2022)

16. National Clinical Trial. Effect of Virtual Reality Glasses as a Distraction Method on Child's Anxiety During Dental Treatment (2019)

17. National Clinical Trial. Use of Virtual Reality Glasses During Anaesthesia in Behavior, Anxiety and Pain Perception of Children (2019)

18. National Clinical Trial. Virtual Reality Technology on Pain Perception and Anxiety Levels in Adolescents and Young Adults During Third Molar Germ Avulsion Surgery (VIDAMO) (2020)

19. National Clinical Trial. Virtual Reality Distraction During Dental Local Anaesthesia Among Children (2020)

20. National Clinical Trial. Virtual Reality and Anxiety in Paediatric Dentistry (2021)

21. National Clinical Trial. Efficacy of VR Distraction During Local Anaesthesia in Children (2021)

22. National Clinical Trial. Virtual Reality in Paediatric Dentistry (2022)

23. National Clinical Trial. Virtual Reality Glasses Integrated with Sign Language on Dental Anxiety Among Children with Hearing Impairment During Pulpotomy Procedure (2023)

24. National Clinical Trial. Oral Surgery Virtual Reality Glasses Study (2024)

25. National Clinical Trial. Virtual Reality Distraction for Dental Anxiety (PILOT) (2023)

26. National Clinical Trial. Virtual Reality Distraction for Dental Anxiety (RCT) (2023)

27. National Clinical Trial. The Effectiveness of Audiovisual Distraction Behaviour Guidance Technique in Children with Dental Anxiety (2020)

28. National Clinical Trial. Virtual Reality vs Nitrous Oxide in Paediatric Dentistry (2023)

29. National Clinical Trial. Effect of VR vs Screens on Children's Dental Anxiety, Pain and Behaviour (2021)

30. National Clinical Trial. Virtual Reality Distraction in Paediatric Patients (2024)

31. Pan African Clinical Trials Registry. Virtual Reality Distraction versus Oral Dexmedetomidine to Minimize Preoperative Anxiety in Pediaric Patients Undergoing Dental Surgery (2022)

**TABLE S2** Study characteristics of included studies.

| **N** | **Publication, year/** | **Journal** | **Study type/ Study design** | **Investigated field** | **Study settings** | **Dental discipline** | **Target population and total number of participants** | **Total number of models/ tests** | **Type of reality** | **Brand and model of device** | **Outcome(s), Primary** | **Outcome(s), Secondary** | **Type of primary outcome(s) and application domain(s)** | **Type of secondary outcome(s) and application domain(s)** | **Key findings** |
| --- | --- | --- | --- | --- | --- | --- | --- | --- | --- | --- | --- | --- | --- | --- | --- |
| Clinical studies | | | | | | | | | | | | | | | |
| 1 | Almugait et al., 2021/ Saudi Arabia | Scientific reports | Clinical /RCT, split-month, 2 arms | Dental practice | Clinical | Conservative dentistry | 21 adults | 42 dental aesthetic injections (21 with topical anaesthetic gel and tell-show-do explanations & 21 with VR headset) | VR | 128 GB Oculus Quest®K3:K4 (Facebook, USA) | Efficacy of VR pain management and pain evaluation via Wong-Baker Faces Pain-rating Scale | Preference for future injection & patient’s heart rate before and after injection | Patient-reported | Clinical | The effectiveness of VR in reducing pain during dental injections is comparable with pain level of topical anaesthesia. |
| 2 | Alshatrat et al., 2019 /Jordan | International Journal of Dental Hygiene | Clinical /RCT, split-months, 2 arms | Dental practice | Clinical | Periodontology | 50 adults | 50 scaling and root planning without anaesthesia (one -half of the mouth with VR & one-half of the mouth without tell-show-do) | VR | iWear Video Headphones, Vuzix®, (Rochester, USA) | Efficacy of VR pain management and pain evaluation via VAS | Patient’s heart rate and blood pressure & patient perception of VR via unstandardised questionnaire | Patient-reported | Clinical | The immersive VR has the potential in becoming a future method for pain control during scaling and root planning procedures. |
| 3 | Alshatrat et al., 2022 / Jordan | International Journal of Paediatric Dentistry | Clinical /RCT, parallel, 2 arms | Dental practice | Clinical | Paediatric dentistry | 54 children, 5-12 years old | 28 children dental treatment with VR & 26 patients’ treatment with tell-show-do | VR | iWear Video Headphones, Vuzix® (Rochester, USA) | Efficacy of VR pain management by subjective patient measurements via Wong-Baker Faces Pain-rating scale | Efficacy of VR pain management by subjective clinicians' measurements via Wong-Baker Faces Pain-rating scale i | Patient-reported | Clinician-reported | VR is an effective and clinically viable technique to reduce pain and anxiety associated with various dental treatments in paediatric dentistry practice. |
| 4 | Aly, et al., 2025/ Egypt | International Journal of Paediatric Dentistry | Clinical /RCT, parallel, 3 arms | Dental practice | Clinical | Paediatric dentistry | 48 children, 7-10 | 48 children underwent dental treatment (16 VR & 16 white noise &16 tell-chow-do) | VR | Shinedown VR glasses (Shinedown®, Dongguan, China). | Efficacy of distraction evaluated by objective measurements (heart rate) | Efficacy of distraction evaluated by subjective measurements of anxiety (Face Image Scale) | Clinical | Patient-reported | VR and white noise could be beneficial in managing dental anxiety and improving behaviour in children with ADHD and could be used as adjunctive strategies to basic BMTs. |
| 5 | Aminabadi et al., 2022/ Iran | JMIR perioperative medicine | Clinical /RCT, parallel, 4 arms | Dental practice | Clinical | Paediatric dentistry | 60 children, 4-6 years old | 60 oral health education section & amalgam restorations (15 pts immersive VR & 15 pts semi-immersive VR & 15 pts non-immersive VR & 15 pts tell-show-do group) | VR | Immersive group: I-glasses 920HR (Iliac Inc, USA); Semi-immersive group: webOS TV (LH590V, LG, Seoul, South Korea); No immersive VR group: Galaxy Tab A7 Lite (Samsung, South Korea) | Efficacy of different levels of VR in comparison with conventional behaviour on children's anxiety during amalgam restoration via SCARED questionnaire. | Efficacy of different levels of VR in comparison with conventional behaviour on children's oral health education. | Patient-reported | Clinical | Sequence of positive behaviour among groups: Immersive VR → Semi-immersive VR → Non-immersive VR→ tell-show-do. Oral health education using VR resources can improve oral health status in children. |
| 6 | Aziz et al., 2020/ Pakistan | Journal of the college of physicians and surgeons Pakistan | Clinical / RCT, parallel, 2 arms | Dental practice | Clinical | Conservative dentistry | 50 adults | 100 topical anaesthesia (50 administration with VR & 50 infiltration without audiovisual distraction via TV) | VR | Bobo VRZ4-VRBOX (Shenzhen Ikeemi Technology Co., China) | To determine the effect of audiovisual distraction on intensity of pricking pain at the intraoral injection site. | Comparison of pain perception (VAS) between the control side (topical anaesthesia alone) and the experimental side (topical anaesthesia) | Patient-reported | Patient-reported | Audiovisual device was found effective in decreasing the pricking pain sensed by the patients during infiltrate anaesthesia, but the score remained within the same pain category. |
| 7 | Bagher et al., 2023/ Saudi Arabia | Journal of clinical paediatric dentistry | Clinical / RCT, parallel, 2 arms | Dental practice | Clinical | Paediatric dentistry | 36 children, 6-14 years old | 18 VR group & 18 treatments during watching a video on normal screen during dental prophylaxis | VR | Lucky Goldstar 360 virtual reality headset (LG Electronics, South Korea) | Efficacy of VR distraction evaluated by objective measurements (heart rate and salivary cortisol levels) | Efficacy of VR distraction evaluated by subjective measurements (Venham Anxiety and Behavioural Rating Scale) | Clinical | Clinician-reported | The VR distraction significantly reduced the salivary cortisol levels of 6 to14 year- old children during dental prophylaxis treatment. No statistically significant difference in heart rate and VABRS between groups observed. |
| 8 | Anholonomy et al., 2024/ Iran | International journal of dentistry | Clinical / RCT, split-mouth, 2 arms | Dental practice | Clinical | Paediatric dentistry | 30 children, 6–8 years | 15 VR group & 15 conventional group during pulpotomy | VR | Large VR glasses (LEJI VR Mini glasses, China) | Efficacy of VR distraction evaluated by objective measurements (heart rate) | Efficacy of VR distraction evaluated by subjective measurements (Modified Child Dental Anxiety Scale - for anxiety levels & Wong–Baker Faces Pain Scale for pain perception before and after the intervention). | Clinical | Patient-reported | VR distraction significantly reduced pain and anxiety levels in children undergoing pulpotomy. No significant difference in pulse rate was observed between the VR and control groups. |
| 9 | Borrelli et al., 2021/ USA | Translational Behavioural Medicine | Clinical/ non-randomized clinical trial (pilot study) | Dental practice | Clinical | Conservative dentistry | 23 adults | 46 smoking cessation videos via VR headset during dental cleaning | VR | V2 VR headset (Knoxlabs, USA) | Feasibility and patients’ satisfaction, accessed by questionnaire, of a smoking cessation video through VR headset | Changes in smoking behaviour and motivation to quit smoking at a 1-month follow-up via questionnaire and interview | Patient-reported | Patient-reported | Implementation of the VR intervention did not interfere with care delivery or patient-provider communication. Participants indicated satisfaction with the experience and a positive impact on their willingness to quit and knowledge of smoking's health effects. |
| 10 | Buldur et al., 2020/ Turkey | Pesquisa Brasileiras em odontopediatria e clinical integrate | Clinical/ RCT, crossover, 2 arms | Dental practice | Clinical | Paediatric dentistry | 76 children, 7-11 years old | 38 restorative treatments with VR distraction & 38 restorative treatment with attention protective glasses only. | VR | PlayStation 4 VR, (Sony Inc, Japan) | Dental anxiety and pain evaluation via objective measurements (heart rate score) and subjective measurements (self‐administered questionnaires) | Behaviour during dental visit subjectively evaluated by clinician via Frankl Behaviour Scale | Clinical | Clinician-reported | VR significantly reduced pain and anxiety during local anaesthesia (as measured by heart rate) compared to the protective glasses group. Self-reported measures did not show statistically significant differences between the VR and APC groups. |
| 11 | Ceylan et al., 2024/Turkey | International journal of dental hygiene | Clinical/ RCT, split-mouth, 2 arms | Dental practice | Clinical | Periodontology | 60 adults | 30 scaling and root planning started with VRD and continued without VRD & 30 started without VRD and continued with VRD | VR | Vive Focus Plus (HTC Corporation, Taiwan) | Efficacy of VR distraction evaluated by objective measurements (blood pressure and heart rate) | Subjective evaluation of efficacy of VRD patients’ anxiety, pain and discomfort via VAS | Clinical | Patient-reported | Using VR glasses during scaling and root planning procedures reduced patients' reported pain and discomfort levels. The order of starting the procedure - with or without VR glasses - affected the patients' dental anxiety, with lower anxiety when starting without the glasses first. |
| 12 | Deshmukh et al., 2024 / India | Pesquisa Brasileira em Odontopediatria e Clínica Integrada | Clinical / RCT, parallel, 2 arms | Dental practice | Clinical | Paediatric dentistry | 32 children, 6–8 years | 32 children, divided equally into two groups of 16 each. Group I (Control): conventional manual toothbrush and fluoridated toothpaste using Fone’s technique Group II (Experimental): Colgate Magik AR-assisted sensor toothbrush with interactive mobile application | AR | Colgate Magik AR-assisted toothbrush with embedded sensor; smartphone application (download via QR code) | To determine the effect of Augmented Reality (AR) -based toothbrushing on oral hygiene practices among children. | Reduction in plaque scores and gingival bleeding scores. | Clinical | Clinical | 1)An improvement in oral hygiene status with a significant reduction in the AR-assisted brushing group was observed. 2)The percentage reduction in plaque and gingival bleeding scores was higher in the AR-assisted brushing group 3)Augmented reality guided toothbrushing is an effective method to teach positive oral hygiene behaviour in children. compared to the conventional brushing group. |
| 13 | Du et al., 2022/China |  | Clinical/ RCT, parallel, 2 arms | Dental practice | Clinical | Paediatric dentistry | 128 children, 4 - 9 years | 128 children in need of teeth extraction ( 60 VR helmets & 64 tell-show-do) | VR | VR HTC customized (Vividu Chengdu, China). | Children's dental anxiety (measured by CFSS-DS) and pain perception (measured by Wong-Baker FACES Pain Scale) | Children's behaviour during the procedure (measured by Houpt Scale) and the occurrence of simulator sickness (measured by SSQ). | Patient-reported | Clinician-reported | The use of VR helmets significantly reduced dental anxiety in children, as measured by the CFSS-DS score, which decreased significantly in the VR group after the dental treatment, but not in the control group. |
| 14 | Felemban et al., 2021/Saudi Arabia | Clinics and Practice | Clinical/ RCT, parallel, 2 arms | Dental practice | Clinical | Paediatric dentistry | 50 children, 6-12 years | 50 children in need of infiltration anaesthesia (25 VR& 25 TV) | VR | VR goggles (LG 360 virtual reality headset, LG Electronics, South Korea) | Anxiety, measured by heart rate, and pain, measured by the FLACC behavioural pain assessment scale | Subjective perceptions of the participants evaluated by Wong-Baker FACES pain rating scale | Clinician-reported | Patient-reported | 1. The use of virtual reality (VR) distraction had a similar effect to screen distraction on heart rate levels and pain during buccal infiltration anaesthesia in paediatric patients. 2. Younger subjects and female subjects reported higher pain scores during local anaesthesia administration regardless of the type of distraction used. |
| 15 | Ghobadi et al., 2024/Iran | BMC oral health | Clinical/ RCT, crossover, 2 arms | Dental practice | Clinical | Prosthodontics | 73 adults | 73 patients with implant surgeries (37 VR&36 tell-show-do) | VR | Oculus Quest 2 (Meta Platforms, USA) | Effects of VR on reducing patients' anxiety and pain via subjective assessment via MDAS and numerical rating scale | Patient satisfaction, surgeon distress, memory vividness, and time perception. | Patient-reported | Patient-reported | 1. Virtual reality (VR) effectively reduced patients' anxiety and pain during dental implant surgery compared to no VR. 2. VR reduced the vividness of patients' memories of the procedure one week later. 3. VR reduced patients' perception of the duration of the procedure. |
| 16 | Gómez-Polo et al., 2021/Spain | Journal of clinical medicine | Clinical/ RCT, parallel, 2 arms | Dental practice | Clinical | Paediatric dentistry | 80 children, 5 - 10 years | 80 children during various dental treatment (40 VR& 40 tell-show-do) | VR | Zeiss Cinemizer (Carl Zeiss AG, Germany) | Effectiveness of VR to manage the anxiety and behaviour of paediatric dental patients via Facial Image Scale | Child behaviour assessment via Frankl behaviour rating scale | Patient-reported | Clinician-reported | 1. The use of a VR headset during dental treatment significantly reduced anxiety and improved behaviour in paediatric patients compared to the control group. 2. Baseline anxiety and behaviour were significant predictors of final anxiety and behaviour, independent of the use of VR. |
| 17 | Gujjar et al., 2019/ Malaysia | Journal of anxiety disorders | Clinical/ RCT, parallel, 2 arms | Dental practice | Clinical | Conservative dentistry | 30 adults | 30 patients with dental phobia (15 VR & 15 Informational pamphlet) | VR | Oculus Rift Development Kit 2 (Facebook Inc, USA) | Subjective evaluation of dental anxiety by patients via VAS, dental trait anxiety measured by MDAS and DFS scores. | Behavioural avoidance, heart rate changes, VR experience, dental treatment acceptance, and the number of patients no longer meeting the criteria for dental phobia. | Patient-reported | Patient-reported | VRET was associated with a significantly greater reduction in patients' state anxiety and dental trait anxiety compared to the informational pamphlet (IP) condition and associated with a significantly greater reduction in behavioural avoidance compared to the IP condition |
| 18 | Gurbuz et al., 2024/Turkey | Journal of aesthetic and restorative dentistry | Clinical/ RCT, parallel, 2 arms | Dental practice | Clinical | Periodontology | 58 adults | 58 patients undergoing gingivectomy or genioplasty (28 VR & 30 tell-show-do) | VR | HTC Vive Focus Plus (HTC, New Taipei, Taiwan) | 1. Anxiety levels measured using the MDAS scale 2. Anticipated pain measured using a VAS scale | 1. Pain and discomfort associated with the surgery measured using a VASP scale 2. Surgical procedure time 3. Patient's perception of time | Patient-reported | Patient-reported | 1. VR application did not affect anxiety and pain levels in patients undergoing periodontal surgery. 2. Young adult participants who used VR during their surgery reported high satisfaction and a preference to use it again in the future. 3. The study did not find significant differences in anxiety levels at different time points (immediately after surgery vs. 1 week later), contrary to some previous studies. |
| 19 | Hamdy et al., 2024/Egypt | Journal of Dental Education | Clinical/ RCT, parallel, 3 arms | Dental practice | Clinical | Paediatric dentistry | 45 children, 4-6 years old | 15 children used the tell-show-do technique, 15 children engaged in passive distraction by watching cartoons using a VR headset, and 15 children participated in active distraction by playing games using a controller with the VR headset. | VR | Samsung gear VR, Model-SM-R325NZVAXAR (Samsung, South Korea) | Efficacy of distraction evaluated by objective measurements (heart rate) | Efficacy of distraction evaluated by subjective measurements of anxiety (Wong-Baker Faces Pain-rating Scale) | Clinician-reported | Patient-reported | VR reduced pain and anxiety levels in its passive and active forms. |
| 20 | Heena et al., 2024/ India | Journal of Clinical and Experimental Dentistry | Clinical/ RCT, parallel, 4 arms | Dental practice | Clinical | Paediatric dentistry | 120 children, 4–10-year-old | 120 children randomly divided in 4 Groups(Group 1- LA Administered with While Using Wireless Headphones, Group 2: LA Administer While using Mobile Phone with earphones. Group 3 : LA Administered while incorporating the 3D virtual reality eyewear method . Group 4: LA Applied while using stress ball technique) | VR | VR glasses (NR) | Evaluation and comparison of the efficacy of different behaviour management techniques in reducing pain perception and anxiety in children during injectable local anaesthesia administration. | Assessment of anxiety levels using MCDAS and VARS, pain scores using WBPRS, behavioural scores using HBRS, and physiological parameters such as pulse rate and oxygen saturation levels. | Patient-reported | Clinician-reported | 1. The virtual reality group was the most effective in reducing anxiety and pain in children during dental procedures compared to other methods. 2. The virtual reality group had the lowest pain scores and the highest behaviour scores, indicating better patient comfort and cooperation. 3.The use of VR devices significantly reduced fear and anxiety, improving oral health care delivery. |
| 21 | Kasimoglu et al.,2024/Turkey | European Journal of Paediatric Dentistry | Clinical /RCT, parallel, 3 arms | Dental practice | Clinical | Paediatric dentistry | 90 children, aged 6–10 years | Ninety children (30 per group): Active distraction group with VR glasses (AG) Passive distraction group watching cartoons on a mounted screen (PG) Control group with no distraction (CG) | VR | Custom-designed VR glasses with mobile-based interactive software (Istanbul Technical University & Istanbul University, Istanbul, Turkey) | Evaluation of the effect of VR on pulse rate in children undergoing dental treatment under local anaesthesia. | Improvement in psychological measures (e.g., FIS, CFSS-DS, FBRS) and evaluation of children's perception of VR. | Clinician-reported | Patient-reported | The use of VR during dental treatment significantly reduced anxiety as indicated by a decrease in pulse rates. VR positively contributed to children's adaptation to dental treatment and created a positive attitude by providing a safe atmosphere. The study found improvements in behaviour and anxiety levels across all groups, but the physiological response to VR was most notable. |
| 22 | Kaswindiarti et al., 2022/ Indonesia | Revista Latinoamericana de Hypertension | Clinical/ non-randomized clinical trial | Dental practice | Clinical | Paediatric dentistry | 30 children, 7-9 years | 30 children with extraction indications divided in 2 groups. Group A was treated with virtual reality distraction during the anaesthetic procedure with a jet injector, while group B was not treated with virtual reality distraction during the anaesthetic procedure with a jet injector. In both groups, pain perception was measured after the anaesthetic procedure, utilizing the Wong-Baker faces pain rating scale. | VR | VR Box headset (generic device, manufactured in China) | Pain perception of children during anaesthesia procedures with jet injectors in dental treatment. | Anxiety perception of children during anaesthesia procedures with jet injectors in dental treatment. | Patient-reported | Patient-reported | Virtual reality distraction significantly reduced pain perception in children aged 7-9 years during anaesthesia procedures with jet injectors in dental treatment, with the distraction group having a mean pain score of 3.17 compared to 5.87 in the control group. |
| 23 | Kosem et al ., 2024/Turkey | Paediatric Dental Journal | Clinical/RCT, parallel, 2 arms | Dental practice | Clinical | Paediatric dentistry | 120 children | 120 children aged 7-10 years randomly divided (60 in Experimental Group using VR, 60 in Control Group). Participants underwent tooth extraction or extirpation under local anaesthesia EG used VR glasses with selected cartoons during treatment; CG received standard care Anxiety and fear measured before and after treatment using Children’s Fear Scale (CFS) and Child Anxiety Scale (CAS) | VR | Samsung Gear VR headset with Galaxy S7 smartphone (Samsung Electronics Co., Ltd., Seoul, South Korea). | The effect of virtual reality glasses on anxiety and fear in children during dental treatment. | Comparison of anxiety and fear scores before and after treatment to assess the effectiveness of virtual reality in reducing anxiety and fear. | Patient-reported | Patient-reported | The study found a statistically significant difference in anxiety and fear scores between the experimental and control groups after dental treatment, with the experimental group having lower scores. - Virtual reality glasses were effective in reducing anxiety and fear levels in children aged 7-10 during tooth extraction and extirpation treatment. |
| 24 | Lahti et al., 2020/Finland | JDR clinical and translational research | Clinical/RCT, parallel, 2 arms | Dental practice | Clinical | General dentistry | 277 adults | A randomized controlled single-centre trial was conducted with 2 parallel arms :VR and treatment as usual (TAU). Groups were randomized, following consent, with an allocation ratio of 1:1. | VR | Samsung Gear VR headset with Samsung Galaxy S7 smartphone (Samsung Electronics Co., Ltd., Seoul, South Korea) | The primary outcome was the total score on the Modified Dental Anxiety Scale (MDAS) measured after the intervention. | The secondary outcomes were the scores on the two subscales of the MDAS - anticipatory dental anxiety and treatment-related dental anxiety. | Patient-reported | Patient-reported | 1. A short virtual reality relaxation (VRR) intervention was effective in reducing total and anticipatory dental anxiety in patients attending a primary dental care clinic. 2. In women, the VRR intervention was effective in reducing total dental anxiety and treatment-related dental anxiety compared to the control group. |
| 25 | Majidi et al., 2021/Iran | Iranian journal of psychiatry and behavioural sciences | Clinical/ non-randomized clinical trial | Dental practice | Clinical | General dentistry | 30 adults | Thirty dental clients with odontophobia randomly divided in (15 experimental & 15 control group) | VR | Samsung Gear 360 camera, 2017 Edition (Samsung Electronics Co., Ltd., Seoul, South Korea); HTC VIVE Pro headset (HTC Corporation, Taoyuan, Taiwan) | The effectiveness of virtual reality exposure therapy (VRET) on reducing odontophobia in the experimental group, as measured by the Modified Dental Anxiety Scale (MDAS) at pre-test, post-test, and 45-day follow-up. | The secondary outcome was the stability of the VRET treatment effect, as assessed by the comparison of post-test and follow-up scores in the experimental group. | Patient-reported | Patient-reported | 1. Virtual reality exposure therapy (VRET) effectively decreased odontophobia (fear of dental procedures) in the experimental group compared to the control group. 2. The reduction in odontophobia symptoms in the experimental group was stable at the 45-day follow-up. |
| 26 | Martinez-Bernal et al., 2024/USA | Oral surgery, oral medicine, oral pathology and oral radiology | Clinical/ Feasibility study | Dental practice | Clinical | Oral surgery | 30 adult patients and 8 dental practitioners | 30 patients and 8 medical staff: Patients completed a demographic survey and self-rated their anxiety levels immediately before the VR intervention and then at 1 and 2 minutes afterward. | VR | Oculus Quest VR headset (Meta Platforms, Inc., Menlo Park, CA, USA) | 1. The feasibility and acceptability of the pre-operative VR intervention, as assessed by the patients and medical staff. 2. The change in pre-operative anxiety in adult patients before and after the VR intervention. | 1. Differences in acceptability of the VR intervention across demographic variables such as age, race, and previous VR experience. 2. Differences in pre-operative anxiety across demographic variables such as sex. | Patient-reported | Patient-reported | 1) The pre-operative VR intervention was highly accepted by patients, with patients reporting high levels of acceptability. 2) The VR intervention was effective in reducing pre-operative anxiety in patients. 3) However, the feasibility and acceptability of the VR intervention was mixed among the medical staff, with only 50% finding it feasible. |
| 27 | Mehrotra et al., 2023/India | Journal of the Indian society of pedodontics and preventive dentistry | Clinical / RCT, crossover, 2 arms | Dental practice | Clinical | Paediatric Dentistry | 40 children | Forty children (6–14 years) were divided into two groups: **Group I (mild intellectual disability) ** and **Group II (healthy children) **. Each group was further split into two subgroups (10 children each) based on the distraction technique used. After one month, a cross-over was performed. Anxiety levels were measured using physiological and observational parameters at three time points. | VR | PROCUS ONE Virtual Reality Headset (PROCUS, New Delhi, India) | 1. Pulse rate (beats/minute) 2. Oxygen saturation levels (%) 3. Venham's anxiety rating scale | the comparison of the effectiveness of audio and VR distraction techniques between children with mild intellectual disability and healthy children. | Clinical | Clinician-reported | 1. Audio and VR distraction techniques were effective in reducing dental anxiety in both healthy children and children with mild intellectual disabilities. 2. Audio and VR distractions were more effective in reducing anxiety in healthy children compared to children with mild intellectual disabilities. |
| 28 | Mehrotra et al., 2024/India | Special care in dentistry | Clinical / RCT, crossover, 2 arms | Dental practice | Clinical | Paediatric Dentistry | 20 children | 20 children diagnosed with mild intellectual disability were equally divided into two groups. A cross-over study design consisting of two appointments 1 month apart was employed. In the first appointment, audio and VR distractions were provided to participants in Groups 1 and 2, respectively | VR | PROCUS ONE Virtual Reality Headset (PROCUS, New Delhi, India) | Effectiveness of audio and virtual reality (VR) distraction techniques in reducing dental anxiety in children with mild intellectual disability, | Comparison of the effectiveness of audio and VR distraction techniques, as well as the feasibility and acceptability of these techniques in children with mild intellectual disability. | Patient-reported | Patient-reported | 1. Both audio and virtual reality (VR) distraction techniques were effective in reducing dental anxiety in children with mild intellectual disability. 2. Neither audio nor VR distraction was superior to the other in reducing dental anxiety in this population. |
| 29 | Mladenovic et al., 2021/Serbia | Journal of Stomatology, Oral and Maxillofacial Surgery | Clinical/ non-randomized clinical trial | Dental practice | Clinical | Oral Surgery | 74 adults | 74 respondents (≥20 years) Procedure: Bilateral surgical extraction of impacted mandibular third molars in two phases (2 weeks apart) Phase 1 (Non-VR): Standard procedure Phase 2 (VR): VR goggles used for distraction | VR | Samsung Gear VR headset (Samsung Electronics Co., Ltd., Suwon, South Korea); Relax River VR app (Polyraw Development, Google Play Store) | Pain Levels: Assessed using a face scale Anxiety Levels: Measured via heart rate (wristbands) | Patient Satisfaction: Evaluated through a post-clinical questionnaire | Patient-reported | Patient-reported | The VR distraction concept applied during impacted mandibular third molar surgery under local anaesthesia can help reduce the patients’ anxiety and acute pain levels. This type of distraction can also be applied in other everyday clinical procedures. |
| 30 | Nunna et al., 2019/India | Journal of dental anaesthesia and pain medicine | Clinical/RCT, parallel, 2 arms | Dental practice | Clinical | Paediatric Dentistry | 70 children aged 7-11 | 70 anxious children requiring local anaesthesia for dental procedures were divided into two groups. Group CS (n = 35) received conventional sedation (CS), while Group VR (n = 35) used VR distraction with ANTVR glasses during the procedure. | VR | ANTVR Phone Glass T2 (ANTVR Technology Co., Ltd., Beijing, China); Lenovo Vibe K4 Note smartphone (Lenovo, Beijing, China) (Lenovo Vibe K4 Note, Beijing, China) and earphones (Sennheiser CX 180 Street II (Black), Sennheiser Electronic GmbH & Co. KG. India) | measuring dental anxiety and pain perception in children during dental procedures. | Evaluating patient satisfaction and the effectiveness of VR distraction compared to traditional counter-stimulation methods | Patient-reported | Patient-reported | Pain during LA was less in the VR group measured with VAS, |
| 31 | Ougradar et al., 2019/United Kingdom | British dental journal | Clinical / Cross-sectional | Dental practice | Clinical | Oral surgery | 50 adults | Fifty patients were screened for extractions. All participants had previous experience of extractions without VR. Pre-treatment anxiety levels were recorded using the Modified Dental Anxiety Scale (MDAS). During the procedure, each patient wore a Virtoba VR headset, | VR | Virtoba Virtual Reality Headset (Virtoba, Shenzhen, China); Aquarium VR app (Apple iPhone 6, Apple Inc., Cupertino, USA). | Reduction in patient anxiety levels during dental extractions when using VR as a distraction tool. | Patient willingness to use VR in future dental visits. | Patient-reported | Patient-reported | the views of participants were positive. The majority of participants made comments on how the VR system managed to divert their attention away from the procedure which helped reduce their anxiety levels. Out of all participants, there weren’t any patients who found the VR to be of ‘no benefit’ which suggests that VR holds at least some value as a distraction and anxiety-reducing |
| 32 | Padminee et al., 2022/India | International Journal of Paediatric Dentistry | Clinical/RCT, parallel, 2 arms | Dental practice | Clinical | Paediatric Dentistry | 70 children | 70 children requiring dental treatment under (LA) for three visits were enrolled in RCT. divided into two groups: Group A received BR distraction and Group B received AV distraction. during LA delivery in the first two visits. No intervention was provided during the third visit. Outcomes were measured using heart rate and the Chotta Bheem-Chutki (CBC) cartoon-based anxiety scale. | VR | iTek vision virtual reality 3D goggles (iTek™, H.K. Wenpod Industrial Co., Ltd., Hong Kong, China); Apple iPhone 7 Plus (Apple Inc., Cupertino, CA, USA) | Heart Rate (HR) | Chotta Bheem-Chutki (CBC) scale scores | Clinician-reported | Patient-reported | AV group had statistically significant higher mean heart rates than BR group (P < .001) during needle penetration and post-intervention, respectively. CBC scale showed no statistical differences between the groups. |
| 33 | Prakash et al., 2025/India | International Journal of Paediatric Dentistry | Clinical/RCT, parallel, 3 arms | Dental practice | Clinical | Paediatric Dentistry | 159 children | 159 children (, 3-13 years) underwent dental treatment (53 VR & 53 clown dentist & 53 tell-show-do) | VR | PA15LF53A Phone Glass T2 (ANTVR, China) | Subjective evaluation of dental anxiety by patients via Chhota Bheem-Chutki (CBC) Scale | Objective evaluation of children anxiety via heart rate | Clinician-reported | Patient-reported | The anxiety and hemodynamic parameter were lower in both intervention groups (VR and dental clown) than those of the control group. |
| 34 | Qin et al., 2022/China | Journal of dental research | Clinical /RCT, parallel ,2-arm | Dental practice | Clinical | Oral surgery | 96 adults | 96 eligible patients with controlled hypertension who needed tooth extraction were randomized to the VR or standard care group by stratified randomization of anxiety grade and gender | VR | Pico G2 VR headset (Pico Interactive Inc., San Francisco, USA and Beijing, China) | The primary outcome was the change in mean arterial pressure | the secondary outcome was the change in heart rate during tooth extraction. | Patient-reported | Clinician-reported | 1) The VR technique was effective and feasible for managing blood pressure and heart rate in patients with hypertension undergoing tooth extraction. 2) Compared to standard care, VR significantly reduced the increase in blood pressure and heart rate during the procedure, without increasing the risk of adverse events. |
| 35 | Ran et al., 2021/China | Trials | Clinical /RCT, parallel, 2 arms | Dental practice | Clinical | Paediatric dentistry | 120 children | 120 children aged between 4 and 8 years to identify the comparative efficacy of VR and tell-show-do (TSD) to improve behavioural management during dental procedures. | VR | HTC VIVE VR headset (HTC Corporation, Taoyuan, Taiwan); ASUS gaming notebook (ASUSTeK Computer Inc., Taipei, Taiwan) | The primary outcomes were anxiety, pain, and compliance scores as measured by (CFSS-DS), (WBFS), and (FBRS). | The secondary outcome was the length of the dental procedure. | Clinician-reported | Clinician-reported | 1. The use of virtual reality (VR) significantly reduced the anxiety and pain of children undergoing short-term dental procedures and improved their compliance. 2. The length of the dental procedure was shorter in the VR group compared to the control group. 3. The overall patient satisfaction was significantly higher in the VR group compared to the control group. |
| 36 | Romalee et al.,2023/Taiwan | Archives of gerontology and geriatrics | Clinical /RCT, parallel, 3 arms | Dental practice | Clinical | General dentistry | 75 adults | 75 patients (61 completed)- Participants randomly assigned to lecture-based, MAR, and control groups. - Lecture-based group received a one-hour lecture; MAR group used a mobile app. - Control group did not receive education. - | AR | MAKAR app (Taipei, Taiwan) | Oral healthcare-related knowledge and self-efficacy | Oral health status (plaque index, gingival index, tongue coating index), System usability score | Educational | Educational | 1.MAR-integrated oral health education showed potential in improving oral health-related knowledge, self-efficacy, and oral health status among community-dwelling older adults. 2. The MAR approach led to superior retention of oral healthcare-related knowledge and self-efficacy compared to the lecture-based approach. 3. The MAR group had a slightly greater increase in self-efficacy compared to the lecture-based group. |
| 37 | Salamal et al., 2024 / Egypt | BMC Oral Health | Clinical/ RCT parallel, 2 arms | Dental practice | Clinical | Paediatric dentistry | 40 children, aged 5–7 years | Two groups of 20 each: Group I (Intervention): VR glasses (SHINECON VR) showing cartoons in sign language Group II (Control): Conventional Show-Tell-Do technique with positive reinforcement. Procedures: Local anaesthesia, pulpotomy, and stainless-steel crown restoration under rubber dam isolation | VR | VR headset: SHINECON VR (Shenzhen Shinecon Digital Technology Co., Ltd., China) Cartoons with sign language preloaded via QR-code app interface | Assess the effectiveness of virtual reality glasses in reducing dental anxiety in children with hearing impairment during pulpotomy treatment compared to conventional management techniques. | 1)Physiological parameter: Heart rate 2) Objective parameter: Venham Clinical Anxiety Rating Scale 3)Subjective parameter: Modified Facial Affective Scale | Patient-reported | Clinician-reported | 1) No significant differences were found in heart rate or Venham Clinical Anxiety Scale scores between the VR group and the control group during dental procedures.2) Anxiety levels by Facial Affective Scale remained comparable between the groups |
| 38 | Shams et al.,2023/India | Journal of Pharmacy and Bioallied Sciences | Clinical /RCT, parallel ,3 arms | Dental practice | Clinical | Paediatric dentistry | 45 children aged 6-10 years. | Three groups: control (tell-show-do), audio distraction, and VR distraction. - Anxiety measured using modified Venham's anxiety rating scale (MVARS) pre- and post-treatment. - Distraction techniques: audio stories/music and VR boxes. - Topical anaesthetic gel and local anaesthetic solution administered. | VR | Shinecon VR Play headset (Shinecon, Shenzhen, China) | Assess the effectiveness of audio and virtual reality (VR) distraction techniques in managing paediatric dental patients by reducing anxiety | Compare the efficiency of audio and VR distraction techniques in reducing anxiety levels before and after treatment | Operator-related | Operator-related | 1.All three groups (control, audio distraction, and VR distraction) showed a statistically significant reduction in anxiety levels from pre-treatment to post-treatment. 2.The VR distraction group experienced the greatest reduction in anxiety, outperforming the other methods. 3. All three methods were similarly effective at lowering anxiety, but VR distraction was more effective in reducing anxiety levels. |
| 39 | Shetty et al., 2019/India | Journal of clinical paediatric dentistry | Clinical /RCT, parallel ,2-arm | Dental practice | Clinical | Paediatric dentistry | 120 children aged 5–8 years | 120 children, with ASA I and II scheduled for invasive dental procedures, were randomly assigned to a control group (no VR distraction) and a study group (VR distraction), with 60 children in each. State anxiety levels were assessed in both groups. | VR | i-glasses 920HR (Ilixco Inc., Menlo Park, CA, USA). | 1. Pain perception during dental treatment, as measured by the Wong Baker Faces pain rating scale. 2. State anxiety levels during dental treatment, as measured by the revised version of the Modified Child Dental Anxiety Scale | Salivary cortisol levels before, during, and after dental treatment. | Patient-reported | Patient-reported | VR distraction significantly reduced pain perception and state anxiety in children during short dental treatments compared to conventional behaviour modification techniques VR distraction led to a greater decrease in salivary cortisol levels, a physiological measure of stress, compared to conventional techniques. |
| 40 | Sorribes et al., 2023/Spain | Journal of the American dental association | Clinical / RCT, parallel, 3 arms | Dental practice | Clinical | Oral Surgery | 275 adults | 275 patients who had to undergo surgery for third-molar extraction participated in a randomized controlled trial and were divided into 3 parallel groups: music therapy intervention (n 91), VR intervention (n 93), and control (n 91). | VR | Bnext 3D VR Headset (Bnext, China) | to evaluate the effects of virtual reality (VR) and music therapy on reducing anxiety and perioperative pain in patients undergoing surgical extraction of impacted third molars | to analyse the state and trait anxiety levels before and after the surgery, determine the degree of postoperative pain, and evaluate the effects of music therapy and VR on physiological parameters such as blood pressure, heart rate, and oxygen saturation. | Operator-related | Operator-related | 1. Patients in the music therapy and VR groups showed a greater reduction in anxiety levels after third-molar extraction surgery compared to the control group. 2. The intensity of pain after the intervention was lower in patients in the music therapy group compared to the control group. |
| 41 | Sukotjo et al., 2025/USA | The Journal of Prosthetic Dentistry | Clinical/ RCT, parallel, 2 arms | Dental practice | Clinical | Prosthodontics | 60 adults | 30 participants per intervention group Intervention 1: Traditional implant typodont - representing a 3-unit fixed prosthesis on 3 implants and a single implant-supported crown. Intervention 2: AR e-typodont application with 3D implant model using marker-based AR approach | AR | Samsung Galaxy A20S smartphone (Samsung Electronics Co., Ltd, South Korea) AR Software: Vuforia Engine 10.14 (AR software development kit) 3D Model Source: TurboSquid; Shutterstock | To determine whether the AR e-typodont improves patient understanding of dental implant treatment procedures, components, and sequences compared to a traditional typodont. | To assess the perceived usefulness and interactivity of the e-typodont compared to the traditional typodont. | Educational | Educational | Both the traditional typodont and the AR e-typodont improved participants' knowledge of dental implants. AR e-typodont was significantly more effective in enhancing understanding of implant treatments, components, and sequences compared to the traditional typodont. |
| 42 | Yamashita et al., 2020/Japan | Journal of Oral and Maxillofacial Surgery | Clinical / RCT, parallel, 2 arms | Dental practice | Clinical | Oral surgery | 100 adults | 100 patients were randomly allocated to one of three groups scheduled for impacted mandibular third molar extraction under local anaesthesia: a non-VR group (n = 49), a natural landscape VR group (n = 51), and a video game VR group (n = 80). | VR | Oculus Go, (Oculus VR, Menlo Park, CA). | The study was the change in anxiety levels during the dental procedure, as measured by the visual analog scale (VAS) score. | the change in pain threshold during the dental procedure, as measured using the PainVision device | Patient-reported | Clinician-reported | 1. The content of the virtual reality (VR) presentation affected the degree to which it reduced anxiety and pain during dental procedures. A video game VR presentation was more effective than a natural landscape VR presentation in reducing anxiety and pain. |

| Educational studies | | | | | | | | | | | | | | | |
| --- | --- | --- | --- | --- | --- | --- | --- | --- | --- | --- | --- | --- | --- | --- | --- |
| 1 | Ali et al., 2024/United Kingdom | Journal of Dental Education | Educational / True Experimental | Dental education and research | Educational | Restorative Dentistry | 24 dental students | 24 dental students performing 3 operative tooth-cutting tasks on a VR haptic dental simulator | VR | Virteasy Dental Simulator (HRV Simulation, France) | Accuracy of tooth cutting measured as the percentage of target volume removal within the defined target area. | Volume of outside target area removal (undesired removal of sound tooth structure), and (b) total tooth cutting time. | Operator-related | Educational | 1. Stereoscopic 3D vision significantly improved accuracy in dental tasks compared to 2D vision. 3.The use of 3D vision did not affect task completion time, but it enhanced depth perception and accuracy in tooth cutting. 2.There was a significant reduction in outside target area removal in the first task when using 3D vision. |
| 2 | Alsufyani et al., 2023/ Saudi Arabia | Journal of dental education | Educational / True Experimental | Dental education and research | Educational | Dental radiology | 69 dental students | 34 student VR-based learning & 35 students lecture-based learning | VR | Oculus Quest 2 (Meta, Inc. USA) | Efficacy of VR utilization in dental radiographic anatomical interpretation | Student engagement, learning, and performance with VR via 5-point scale questionnaire | Educational | Educational | Lecture-based students showed better performance in panoramic radiographic anatomy. The positive feedback of VR experience encourages future implementation in education. |
| 3 | Azhari et al.,2024/Saudi Arabia | Journal of Dental Education | Educational / True Experimental | Dental education and research | Educational | Restorative Dentistry | 150 dental students and dental practitioners | 150 (49 dental students, 52 dental interns, 49 faculty members) performed simulating Class I cavity preparation with VR glasses and Meta Quest headset | VR | Meta Quest headsets (Meta Platforms Inc., USA) | Development of a VR dental system for tooth preparation training. | Assessment of pre-VR experiences and perceptions, evaluation of system usability, usefulness, system assessment, and participant attitudes. | Educational | Educational | The virtual-reality dental module system shows promise for improving dental education by enhancing motor skills and procedural dexterity. No significant differences in usability or usefulness scores between the three user groups |
| 4 | de Boer et al., 2019/Netherlands | Simulation in healthcare | Educational / True Experimental | Dental education and research | Educational | Conservative dentistry | 126 dental students | 126 manual dexterity tests (drilling with the standard force feedback and an altered level) | VR | Simodont dental trainer (Moog, Netherlands) | Technical performance of students evaluated via Board of Examinations' requirement | Students' subjective experience and opinions about working with different FFB levels evaluated via questionnaire | Educational | Educational | Students were able to transfer a manual dexterity skill acquired at one level of force feedback to another level. Students noticed the differences in force feedback levels but did not perceive the altered levels as more difficult. |
| 5 | Bui et al. , 2024/USA | Journal of Dental Education | Educational / True Experimental | Dental education and research | Educational | Dental radiology | 60 dental students | 60 first-year dental students (30 VR; 30 MP) Analysed: 28 MP; 27 VR (5 lost to follow-up) MCQs: 10-item test at three time points (T1 pre, T2 immediate post, T3 two-week follow-up) Surveys: Pre- and post-survey, Game Engagement Questionnaire, Presence questionnaire, NASA-TLX. Educational intervention: One-on-one, 30-minute session identifying 30 anatomic landmarks in CBCT data. | VR, MR | Oculus Rift headset + Touch controllers (Meta, USA). VR Software: Immersive View 1.0 (ImmersiveTouch Inc., USA) MP Software: Dolphin Imaging 11.95 Premium (Dolphin Imaging Inc., USA) | To compare the effectiveness of immersive virtual reality (VR) and three-dimensional multiplanar (MP) reconstructions in teaching cone beam computed tomography (CBCT) interpretation to dental students. | To assess subjective experiences such as distraction, realism, involvement, and physical demand using questionnaires like the Presence and NASA-TLX. | Educational | Educational | 1. Both VR and MP educational methods significantly improved student learning outcomes in terms of test performance and informational recall. 2.VR and MP were equally effective in terms of objective learning outcomes, with no significant differences in performance between the two groups. 3.The VR group experienced decreased distraction, increased realism, and increased involvement compared to the MP group, indicating a better quality of learning experience. |
| 6 | Bandiaky et al.,2025/France | International Endodontic Journal | Educational / True Experimental | Dental education and research | Educational | Endodontics | 90 dental students | 90 third-year dental students in two parallel groups completed exercises both with and without a VR headset. | VR | Meta Quest (Meta Platforms Technologies, USA) | Evaluate the impact of using the VirTeaSy Dental simulator with a VR headset on student performance during access cavity preparation, focusing on haptic parameters (total time, milling time, overall progress, accuracy, internal volume, outside volume). | Assess students' perceptions of the experience; examine the side effects associated with VR headset use. | Operator-related | Operator-related | 1.Students performed better in access cavity preparation without the VR headset, showing greater accuracy and fewer errors. 2.The use of VR headsets led to more failures to complete exercises within the time limit and reported side effects such as dizziness and nausea. 3.The study concludes that full immersion with VR headsets negatively impacts performance in complex tasks, suggesting that non-immersive setups are more effective for motor skills acquisition. |
| 7 | Cano-Martins et al., 2025 / Brazil | Dentomaxillofacial Radiology | Educational / True Experimental | Dental education and research | Educational | Dental radiology | 32 dental students | 32 dental students (first and second year) Theoretical 90-minute lecture on panoramic imaging for all participants. Group 1 (n=16): traditional method only Group 2 (n=16): semi-immersive VR training using Qbion AB software Practical training: each student acquired one panoramic radiograph on two anthropomorphic phantoms using VistaVoxS 3D device | VR | VR Software: Qbion AB-Umeå (Qbion AB, Sweden) Panoramic Device: VistaVoxS 3D (Dürr Dental SE, Germany) Input Device: 3D mouse Display: Barco NioColor 2MP Dental monitor (Barco, Belgium) | Effectiveness of semi-immersive VR software in reducing positioning errors in panoramic radiography. | Retention of knowledge in identifying anatomical structures and detecting positioning errors. | Educational | Educational | The semi-immersive VR software demonstrated potential benefits for dental students by reducing positioning errors in panoramic radiography. The VR group exhibited better retention of knowledge, with a higher ability to identify positioning errors (68.8%) compared to the traditional group. |
| 8 | Cheng et al., 2025/China | Journal of Dental Education | Educational / True Experimental | Dental education and research | Educational | Periodontology | 90 dental students | Participants randomly assigned in 90 students divided in three groups: VR teaching group, traditional jaw model teaching group, and combined VR + jaw model group -All groups learned scaling and root planning (SRP) techniques Final -post-exam teaching survey questionnaire assessing satisfaction and perceived learning focus areas operation examination on jaw model including scoring by three blinded raters | VR | Beijing Zhonghui Virtual Reality Technology Research Institute, China (Oral digital virtual simulation training system) | Performance in the final operation examination (total scores and specific scores such as force mode, direction, and motion amplitude). | Results from the teaching survey questionnaire (ability to grasp course knowledge and integration with practice). | Educational | Educational | 1.Combined VR and jaw model group achieved the highest total scores and significantly outperformed single-method groups on force mode, direction, and motion amplitude 2.Model group scored significantly higher than VR only group on fulcrum scores; VR group scored higher than model group on instrument angle scores 3.Questionnaire showed VR group rated higher in fully grasping course knowledge, while model and combined groups rated better on integration with practice |
| 9 | Choi et al , 2025 / South Korea | BMC Medical Education | Educational / True Experimental | Dental education and research | Educational | Oral surgery | 40 dental students | 40 student trained tooth extraction (20 VR & 20 conventional learning) | VR | MetaQuest 2 (Meta, USA) | Understanding of surgical procedure evaluated via questionnaire on a 5-point scale | Satisfaction with observation experience via questionnaire | Educational | Educational | VR clinical observation using 360° videos might be an effective teaching method for students. However, to allow interaction between professors and students during clinical observations, using it along with conventional clinical observation is necessary. |
| 10 | Collaço et al., 2021/Brazil | Journal of dental education | Educational / True Experimental | Dental education and research | Educational | Oral surgery | 163 dental students | 163 trainings of inferior alveolar anaesthesia across 4 groups across 2 phases using a haptic VR simulator, with haptic feedback On or Off. | VR | VIDA Odonto® (Intercompany, Brazil) | Objective technical skill measured by the simulator, including task execution time, insertion accuracy, needle angle, and needle depth | Subjective perceptions of the participants regarding syringe handling and simulator sickness via questionnaire | Educational | Educational | Immersive training, either in the preceptorship or training phase, led to better performance in administering the inferior alveolar nerve block anaesthesia, in terms of accuracy, confidence, execution time, needle insertion point, and insertion accuracy. |
| 11 | Daud et al., 2023/Qatar | BMC medical education | Educational / True Experimental | Dental education and research | Educational | Conservative dentistry | 23 dental students | 46 class I cavity preparation (23 with VRHS, then repeated with phantom head & 23 with phantom head and then using VRHS) | VR | SIMtoCARE Dente®, (SD001, Netherlands) | Evaluation of the impact of VRHS on the learning experiences and perceptions of dental students via questionnaire | Comparison of student performance on cavity preparation tasks between the VRHS and conventional simulation environment (CSE) groups. | Educational | Educational | Dental students generally perceived VRHS as a useful tool for enhancing their manual dexterity and learning experiences in restorative dentistry. |
| 12 | Daud et al.,2024/Qatar | BMC Oral Health | Educational / True Experimental | Dental education and research | Educational | Restorative Dentistry | 21 dental students | Two operative tasks per student: Class I cavity preparation on typodont teeth in a conventional simulated environment (CSE) and on a virtual lower first molar (#36) using a VRHS; plus, manual dexterity block exercises (“channel” and “cross” shapes) on VRHS. Study Method: Homogeneous purposive sampling; three face-to-face focus groups; audio-recorded and transcribed; inductive thematic analysis | VR MR | SIMtoCARE Dente® (SD001) mixed-reality haptic simulator; Vreeland, The Netherlands | To understand dental students' perceptions of the impact of Virtual Reality Haptic Simulators (VRHS) in pre-clinical restorative dentistry. | To assess the effectiveness of VRHS in enhancing students' learning experience, particularly in preparing a standard class I cavity, focusing on themes such as manual dexterity, assessment, sequence, training, and realism. | Educational | Educational | 1.The study identified five primary themes related to VRHS in pre-clinical restorative dentistry: manual dexterity, assessment, sequence, training, and realism. 2.The most frequently mentioned theme by students was the need for enhanced realism in VRHS to better simulate real-life dental procedures and learning environments. 3.The study suggests that VRHS are valuable for training and feedback but require further development to improve realism and interactive learning experiences. |
| 13 | Diegritz et al.,2024/Germany | International Endodontic Journal | Educational / Quasi-Experimental | Dental education and research | Educational | Endodontics | 57 dental students | Developed XR app featuring μCT scanned human teeth with interactive 3D models viewable on mobile devices (AR) and VR headsets Application allows manipulation of tooth models, tissue transparency, and detailed anatomical information Students pre- and post-evaluated via Likert scale questionnaires assessing usability, motivation, learning success, and comparisons with traditional methods | AR MR VR | VR headset: Pico Neo 3 (Pico Interactive, USA) Mobile devices: Samsung Galaxy Tab 8A (South Korea) Application developed using Unity software | Evaluation of whether the application meets students' expectations and its effectiveness in teaching root canal anatomy. | Assessment of user-friendliness, motivation for learning, contribution to learning success, and comparison to traditional learning methods. | Educational | Educational | The app met students’ expectations regarding usability and learning support (no significant difference between pre- and post-use expectations) Rated highly user-friendly (98.2%) and motivating (100%) for learning root canal anatomy Students appreciated interactive 3D visualization aiding deeper understanding beyond conventional methods like illustrations/radiographs |
| 14 | Duan et al.,2024/China | BMC Medical Education | Educational / Quasi-Experimental | Dental education and research | Educational | Conservative Dentistry | 98 dental students | 98 students: – Access cavity preparation on (a) plastic teeth, (b) 3D-printed teeth, (c) virtual simulation system, each for 20 min in random sequence – Expert scoring of physical teeth, machine scoring of virtual procedure – Two student questionnaires on training preferences and tool advantages/disadvantages | VR | Unidraw virtual simulation machine (China); | Evaluate the effect of 3D printed teeth and virtual simulation systems on pre-clinical access cavity preparation training. | Comparison of scores between plastic and 3D printed teeth. Evaluation of the virtual simulation system's performance (valid and invalid area removal ratios). Detailed scores for access cavity preparation aspects (cavity location, size, outline, etc.) | Educational | Educational | 1. No significant differences were found between plastic and 3D printed teeth scores, while virtual simulation achieved a high valid area removal ratio. 2. Most students preferred the combination of all three training methods, with virtual simulation suggested as the first step. 3. The optimal training sequence is virtual simulation followed by plastic teeth and then 3D printed teeth, with virtual simulation not fully replacing traditional methods. |
| 15 | Dundar et al., 2025 / | BMC Oral Health | Educational / Quasi-Experimental | Dental education and research | Educational | Prosthodontics | 200 dental students | 200 dental students total: A)100 fourth-year students (VR training group) B)100 fifth-year students (control group - no VR training Additional: 25 students from each group (50 total) for phantom model comparison Survey participants: 124 fourth-year students. 50 phantom model preparations (plastic typodont teeth) 200 clinical preparation samples (STL files from patient work) 101 virtual reality practice sessions recorded v | VR | Simodont Dental Trainer (SDT) (Moog Inc., Nieuw-Vennep, Netherlands) | Investigate the impact of virtual reality simulations on students' clinical preparation skills. | Compare tooth preparation skills between students trained with virtual reality simulators and those trained with traditional methods | Educational | Operator-related | 1. The study found that dental students trained with virtual reality simulators demonstrated better tooth preparation skills compared to those trained with traditional methods. 2. Fourth-year students who used virtual reality simulators achieved preparation angles closer to the ideal than fifth-year students who only received traditional training. 3. The results suggest that integrating virtual reality simulators into the dental education curriculum could enhance students' readiness skills |
| 16 | Farag et al., 2022/Saudi Arabia | BMC Oral Health | Educational / Pre-Experimental | Dental education and research | Educational | Restorative dentistry | 21 dental students | 21 dental students performed Class I cavity preparations with HVRS | VR | HVRS Simodont dental trainer (Moog Industrial Group, Netherlands) | Impact of HVRS on dental students' psychomotor skills acquisition | Improvements in cavity preparation performance, including decreased preparation time and increased overall quality scores, as well as improvements in specific cavity design features | Operator-related | Educational | A statistically significant decrease in the time taken for cavity preparation after HVRS training. 2. A statistically significant increase in the overall quality of the cavity preparations after HVRS training. |
| 17 | Felszeghy et al., 2023/Finland | International journal of computerized dentistry | Educational / Quasi-Experimental | Dental education and research | Educational | Prosthodontics | 40 dental students | 40 students (20 HVRS trainings & 20 phantom head training) | VR | Simodont® Dental Trainers (Nissin Dental Products Inc., Japan) | Effectiveness of each training modality, success rate and utilised time | Evaluation of tooth preparation metrics, such as occlusal reduction depth, preparation margin depth, and convergence angle, for a crown preparation task in both the VR-haptic simulator and on physical Frasco teeth. | Educational | Educational | VR-haptic training before conventional phantom head simulation laboratory exercises had a significant positive effect on the accuracy and success of the tooth preparation process for dental preclinical undergraduates. |
| 18 | Fu et al., 2024/China | BMC medical education | Educational / pre-experimental | Dental education and research | Educational | Conservative dentistry | 411 dental students and dental practitioners (355 students& 54 teachers) | 157 students completed online and VR education. 101 student - traditional education. 97 students - blended learning group. Feedback of 54 teachers on online teaching | VR | Simodont® Dental Trainers (Nissin Dental Products Inc., Japan) | Students' and teachers' perceptions of online theoretical teaching and virtual simulation teaching. | Comparison of student performance between the blended teaching method of online teaching combined with virtual simulation teaching and the traditional teaching method. | Educational | Educational | 1. Students were generally satisfied with the online courses and virtual simulation platform teaching, while teachers held more conservative and neutral attitudes towards online teaching. 2. The blended learning approach combining online teaching and virtual reality technology did not significantly improve students' overall performance compared to traditional teaching, but the virtual simulation platform was well-received by students. |
| 19 | Fudalej et al., 2024/Poland | Orthodontics & Craniofacial Research | Educational / True Experimental | Dental education and research | Educational | Orthodontics | 38 dental students & dental practitioners | 38 participants including orthodontists (44.7%), dentists (34.2%), dental students (15.8%), and dental technicians (5.3% Development of MR visualization application for impacted canine teeth using cone-beam CT (CBCT) scan data. Visualization used in clinical course participants for treatment planning and workflow communication Feedback obtained via 14-item questionnaire with 5-point Likert scale responses assessing perceived benefits and limitations | MR | HoloLens 2 goggles (Microsoft, USA) | Effectiveness of mixed reality-based technology in improving treatment outcomes for impacted teeth. | Patient satisfaction, perceived benefits such as precise planning, reduced need for orthodontist presence, and improved patient education. | Clinician-reported | Patient-reported | High acceptance and perceived advantages for precise planning of attachment placement, surgical procedure, traction direction, appliance design, and reduced adverse effects. Visualization improved stakeholder communication including patient education and dental student learningç Major disadvantages noted were high cost of HoloLens 2 and manual labour for CBCT data segmentation |
| 20 | Grad et al., 2023/Poland | Anatomical sciences education | Educational / True Experimental | Dental education and research | Educational | Conservative dentistry | 32 dentist (24 certified dentists, 8 trainee dentists) | 99 anatomical models of molar crowns (96 specimens and 3 reference model) | MR | HoloLens 1 (Microsoft, USA) | Quantitatively evaluated by measuring the maximum Hausdorff distance between the superimposed scans of the anatomical reference tooth model and the model after reconstruction | Assessment of aspects affecting the outcomes - clinical experience, tooth anatomy and participants perception via questionnaire | Educational | Educational | The results showed that the most accurate but slowest reconstruction was achieved with the use of 3D-printed reference models and that the results were not affected by other aspects considered. For this method, the Hmax was observed to be 630 μm (p = 0.004). |
| 21 | Hamama et al.,2024/ Hong Kong | BMC Medical Education | Educational / True Experimental | Dental education and research | Educational | Conservative Dentistry | 76 dental students | 76 students randomly divided into two groups (38 each). VR-Start group: VR simulator first, then natural teeth practice. Natural Tooth-Start group: natural teeth practice first, then VR simulator. 4-point Likert scale questionnaire evaluated self-confidence, clinical skills perception, and patient care views. | VR | Simodont Dental Trainer (Moog B.V., Netherlands) | Enhancement of perceived self-efficacy (confidence) and perceived learning outcome (clinical skills) | Students' perception of providing better care to patients in the future and facilitation of haptic feedback for dental procedures | Educational | Educational | 1.The study found no significant difference in students' responses between the VR-Start group and the Natural Tooth-Start group. 2.The majority of students preferred practicing on natural carious teeth, indicating that while VR is useful, it does not replace traditional methods. 3.The VR haptic-enhanced simulator improved clinical skills and confidence but lacked the tactile sensation of natural teeth. |
| 22 | Haas-Stroede et al., 2025/USA | Journal of Dental Education | Educational / True Experimental | Dental education and research | Educational | Oral surgery | 44 dental students | 44 Second-year dental students were randomized into VR and control groups. - Both groups received traditional lecture instruction at baseline. - The VR group underwent additional training with VRIAN, completing 10 sequential injections. - Both groups participated in a peer-to-peer injection exercise. | VR | VRIAN （ Immersive Touch., Chicago, USA) | Determine construct validity of VRIAN injection simulation to improve needle positioning for IANB delivery. | Evaluate transfer validity of VRIAN simulation to assess transferability of skills to simulated clinical experience. | Educational | Educational | 1.The VR group improved their injection positioning by a mean of 0.63 mm in 3D distance and 0.55 mm in the x dimension over 10 attempts. 2.The VR group showed a significant increase in confidence compared to the control group. 3.Both groups were equally successful in providing anaesthesia during the peer-to-peer exercise. |
| 23 | Hu et al., 2025/China | BMC Medical Education | Educational / True Experimental | Dental education and research | Educational | Endodontics | 123 dental students | Experimental group received REPs training using a virtual simulation (VS) platform featuring interactive demonstration, decision-making, operative steps, and gamification Control group received traditional teaching with lecture plus videos and guidelines Both groups took pre- and post-intervention theoretical and practical exams; students completed questionnaires on perceptions | VR | Custom-developed VS platform (The Virtual Simulation Experimental Teaching Centre of Stomatology, Chengdu, China) | Improvement in theoretical and operational examination scores. | Improvement in students' perceptions and attitudes towards the REPs-VS platform. | Educational | Educational | 1. The VS experimental group scored significantly higher on post-teaching theoretical (82.1 vs. 72.3) and practical exams (90.7 vs. 87.7) compared to traditional group 2.High student acceptance (98%+) of VS platform for enhancing knowledge, skills, communication, and motivation 3.VS modules provided interactive, gamified, and self-paced learning reducing stress and enhancing engagement |
| 24 | Huang et al., 2022/China | Journal of dental education | Educational / pre-experimental | Dental education and research | Educational | Orthodontics | 82 dental students | A survey was designed to collect feedback information about the experiences and efficacy of the VR application on the orthodontic training of undergraduate dental students. At the end of the training, every participant was asked to answer the questionnaire on SoJump | VR | VR system (Shanghai VR-Sens Intelligent Technology, Shanghai, China) | VR session was to help dental students become familiar with the VR operating procedures. | Providing opportunities for participants to be familiar with the standard operating procedures of orthodontic treatment. | Educational | Educational | 1. The VR system had an above-average system usability score of 82.5 ± 10.1. 2. The participants provided positive feedback on the VR system's user experience, perceived usefulness for learning orthodontics, and ease of manipulation. 3. Male students exhibited stronger interest and were more capable in manipulating the VR system compared to female students. |
| 25 | Huang et al., 2023/China | Peer J | Educational / pre-experimental | Dental education and research | Educational | Implant dentistry | 119 dental students | A survey instrument was designed to collect feedback information about the experiences and usability of the VR technology on the dental implant learning experience of undergraduate dental students. Every participant was asked to answer the questionnaire on SoJump (Ranking, Changsha, China), | VR | VR-SENS VR Implant Tutorial Software (Shanghai VR-Sens Intelligent Technology, Shanghai, China) | to evaluate the usability and feasibility of the VR application for training dental implants among undergraduate dental students | to investigate the dental students' perceptions and acceptance of the VR application for learning dental implants. | Educational | Educational | 1. The VR system used for dental implant training was highly usable, with an average SUS score of 82.00 ± 10.79, indicating it was ranked in the top 10th percentile of usability. 2. The dental students generally had a positive perception of the VR system for dental implant training, acknowledging its usefulness and expressing interest in using it. |
| 26 | Im et al.,2025/South Korea | BMC Medical Education | Educational / True Experimental | Dental education and research | Educational | Dental radiology | 79 dental students | 79 dental hygiene students of 2–3 years were randomly assigned to two group. control group (n = 39): used Google Cardboard for VR, experimental group (n = 40): used 3DOVR-DR User experience assessed via 22-item Likert-scale questionnaire | VR | 3DOVR-DR: customed developed; HMD: Oculus Quest 2 (Meta, USA) Development engine: Unity 3D; 3D assets modified in Blender | Overall user experience rating (mean Likert score) of VR tool | Domain-specific ratings for usefulness, ease of use, ease of learning, and satisfaction | Educational | Educational | The 3DOVR-DR tool provided an immersive experience for simulating and learning the dental radiography process within a VR setting. User-experience ratings were significantly higher in the experimental group using 3DOVR-DR The 3DOVR-DR tool shows potential as a learning medium for intraoral radiography education. compared to the control group using 360° VR video. |
| 27 | Iwanaga et al., 2021/Japan | Clinical Anatomy | Educational / True Experimental | Dental education and research | Educational | Anatomy | 30 dental students | Experience of a distant online lecture on a surgical procedure and related anatomy in a 3D VR workspace and to compare it with reported teaching methods, that is, an in-person class and a Zoom online class. | VR | Oculus Quest 2 headset (Meta Platforms, Inc., Menlo Park, CA, USA); Spatial VR workspace (Spatial Systems Inc., New York, USA). | The primary purpose of this study is to share the experience of a distant online lecture on a surgical procedure and related anatomy in a 3D VR workspace | To compare it with reported teaching methods, that is, an in-person class and a Zoom online class. | Educational | Educational | 1. The use of a 3D virtual reality (VR) workspace with a live communication tool can be an effective alternative teaching method for anatomy and surgical education, especially during the COVID-19 pandemic. 2. The VR workspace allows for active participation and engagement by attendees, which can improve their understanding of the surgical procedures and anatomy. |
| 28 | Kaluschke et al., 2023/ Finland | PLoS ONE | Educational / Quasi-Experimental | Dental education and research | Educational | Endodontics | 40 dental students | 40 participants divided randomly into one of the 4 groups: Group 1: Stereoscopic 3D & hand-tool alignment • Group 2: Monoscopic 3D & hand-tool alignment • Group 3: Stereoscopic 3D & hand-tool misalignment • Group 4: Monoscopic 3D & hand-tool misalignment | VR | HTC Vive headset (HTC Corporation, Taoyuan, Taiwan); Geomagic Touch X haptic devices (3D Systems, Rock Hill, SC, USA); Unity3D-based simulator (Unity Technologies, San Francisco, USA). | 1. Learning gains between pre-and post-testing on realistic plastic teeth (to measure teaching effectiveness) 2. Correlation between real-world pre-testing score and initial simulator performance (to measure suitability for assessment) | The secondary outcomes are the impact of stereoscopic 3D rendering and hand-tool alignment on these primary outcomes. | Educational | Educational | 1. Hand-tool alignment has a positive impact on virtual and real learning gains and improves the accuracy of skill assessment. 2. Stereoscopic 3D has a negative impact on virtual and real learning gains but improves the accuracy of skill assessment. 3. Eye-tooth distance is a confounding variable that explains the counter-intuitive influence of stereoscopic 3D on learning gain. |
| 29 | Kihara et al., 2024/USA | Journal of Dentistry | Educational / pre-experimental | Dental education and research | Educational | Prosthodontics | 24 dental students | Tooth preparation performed on mandibular models with first molars replaced by custom 3D printed teeth Testing three visualization/navigation methods: (1) Traditional (no AR assistance), (2) AR Overlay (ideal tooth shape overlaid), (3) AR Cross-Sectional (additional cross-sectional views and angle guidance) | AR | HoloLens2 headset (Microsoft Corp., Redmond, WA, USA); Unity 3D-based AR navigation system (Unity Technologies, San Francisco, USA) | Accuracy of tooth preparation: measured by surface deviation (distance to ideal tooth surface) and angle differences between prepared and ideal axis | User experience and system usability as measured by SUS and VRSQ scores, and workload metrics such as mental demand, physical demand, temporal demand, performance, effort, and frustration. | Educational | Educational | No significant difference in average surface deviation among the three methods AR cross-sectional approach significantly reduced angle deviation compared to traditional method — better guidance of bur angle AR cross-sectional approach resulted in less over-reduction than traditional and overlay methods, enhancing safety |
| 30 | Kim-Berman et al., 2023/ USA | Journal of dental education | Educational / Quasi-Experimental | Dental education and research | Educational | Anatomy | 90 dental students | All 90 participants completed a 10-MCQs and a pre-survey prior to the educational intervention. Following a brief video orientation on CBCT and anatomy | VR | Immersive View VR software (ImmersiveTouch Inc., Chicago, IL, USA); Oculus Rift headset and Touch controllers (Meta Platforms, Inc., Menlo Park, CA, USA) | the students' performance on multiple-choice questionnaires (MCQs) testing their knowledge of 3D imaging concepts and head and neck anatomy before, immediately after, and 2 weeks after the VR/CBCT educational intervention | the students' subjective experiences and perceptions, as measured by post-intervention surveys, the NASA task load index, and presence questionnaires | Educational | Educational | 1. Dental students showed a significant improvement in their test scores on 3D imaging concepts and head and neck anatomy after the VR/CBCT educational intervention, with the improvement being maintained 2 weeks later. 2. The VR/CBCT educational intervention was highly engaging and contributed to a strong sense of presence for the students. 3. The VR/CBCT educational intervention had a very large effect size on student learning. |
| 31 | Liebermann et al., 2024/Germany | European journal of dental education | Educational / Quasi-Experimental | Dental education and research | Educational | Prosthodontics | 122 dental students & dental practitioners | 59 lecturers and 63 students, Lecturers: Mean age of 36.2 years (±9.0), with a gender distribution of 42.4% male, 55.9% female, and 1.7% diverse. Students: Mean age of 24.3 years (±3.0), with a gender distribution of 34.9% male and 65.1% female. | VR | VR glasses Oculus Quest 2 All-in- one (Meta Quest, Menlo Park, CA, USA) | Participants' evaluations of the virtual prosthetic case planning environment (VCPE) in terms of usability and effectiveness. | Differences in evaluations between various dental schools and between lecturers and students. | Educational | Educational | 1. There were significant differences in the assessment of the virtual prosthetic case planning environment (VCPE) between the four dental schools analysed, as well as between lecturers and students within each dental school. 2. Overall, both lecturers and students evaluated the VCPE positively and recommended its use for teaching, though there were some differences in perception between the two groups. |
| 32 | Liebermann et al., 2020/Germany | Journal of dental education | Educational / pre-experimental | Dental education and research | Educational | Prosthodontics | 63 dental students | Creation of the VR dental teaching environment was divided into & sections: (a) generation of the digital data; (b) creation of the VR dental learning environment for tooth morphologies; and (c) evaluation by preclinical students through questionnaires combined with visual analogue scale and fixed answer options. | VR | HTC Vive Pro headset (HTC Vive, San Francisco, CA, USA) | Students' understanding of dental morphologies compared to traditional textbook learning. | Willingness to invest in VR equipment for personal use. Evaluation of different teaching elements (haptic, auditory, visual) within the VR environment. | Educational | Educational | 1) The VR dental learning environment showed a high level of acceptance among preclinical dental students, who stated they could better understand dental morphologies using it. 2) The haptic and auditory teaching elements of the VR environment were rated most positively by students, more so than the visual information boards. 3) The VR dental learning environment should be integrated as a fixed element in the dental curriculum. |
| 33 | Liebermann et al., 2024/Germany | European journal of dental education | Educational / Quasi-Experimental | Dental education and research | Educational | Prosthodontics | 98 dental students | After using the VCPE, participants completed questionnaires to evaluate various outcomes, including: Perceived immersion Cognitive load Authenticity Learning motivation | VR | Oculus Quest 2 All- in- one (Meta Quest; USA) | The primary outcomes assessed in this study were perceived immersion/presence, cognitive load, motivation, | authenticity of the virtual prosthetic case planning environment. | Educational | Educational | 1. The virtual prosthetic case planning environment was well-received by dental students, with positive results for perceived immersion, cognitive load, authenticity, and high student motivation. 2. Limitations of the virtual environment include the need to reduce cognitive load and address issues of cyber sickness and internet connectivity for some students. |
| 34 | Liebermann 2022/Germany | Journal of dental education | Educational/ True Experimental | Dental education and research | Educational | Prosthodontics | 82 dental students | 82 students were randomly assigned to two groups for cross-over testing of tooth morphology knowledge. Group 1 used VR glasses for learning anterior tooth morphologies, while Group 2 used them for posterior tooth morphologies, in addition to conventional learning. Both groups used VR glasses for 1 week at their convenience. After 3 weeks (anterior test) and 6 weeks (posterior test), students identified teeth and recorded their number and characteristics. | VR | Oculus Quest 2 headset (Meta Platforms, Inc., Menlo Park, CA, USA) | The primary outcome of this study was the effect of virtual teaching using VR on test performance of preclinical dental students, both with and without prior dental experience. | the students' ability to recognize and identify anterior and posterior teeth, as well as their characteristics, as measured by the test scores. | Operator-related | Educational | 1. Additional learning of tooth morphologies using the VR learning environment did not significantly improve test scores compared to conventional learning methods. 2. Students performed significantly better on tests of anterior teeth compared to posterior teeth. 3. Students with prior dental professional experience performed significantly better on the tests than those without. |
| 35 | Liebermann et al., 2023/Germany | Journal of dental education | Educational / pre-experimental | Dental education and research | Educational | Prosthodontics | 82 dental students | Students used a mobile VR learning environment at home for one week. After the intervention, students completed a 21-item questionnaire assessing intuitive handling, supplemental learning, and included 9 predefined questions, 9 VAS questions, and 3 open-ended questions. Responses were analysed descriptively, with normality checked using the Kolmogorov-Smirnov test. | VR | HTC Vive (San Francisco, USA), Oculus Quest # All-in-one (Menlo Park, CA, USA) | Students' perception of understanding dental morphologies using the VR learning environment compared to traditional methods | Usability and technical aspects of the mobile VR learning environment, including handling, technical difficulties, and resolution quality | Educational | Educational | 1. 75.7% of students felt the VR learning environment improved their understanding of dental morphologies compared to traditional methods. 2. Students see significant potential (77.5%) in using VR for teaching other dental topics beyond just tooth morphologies. |
| 36 | Liu et al.,2024/China | Anatomical Sciences Education | Educational / Quasi-Experimental | Dental education and research | Educational | Endodontics | 434 dental students | 434 fourth-year undergraduate dental students (214 control group; 220 virtual simulation–trained group) | VR | Virtual Simulation Training System for Apicoectomy (developed by the Endodontic Faculty, West China Hospital of Stomatology, Sichuan University, Chengdu, China; copyright No. 2024SR0214015) | training enhancement in preclinical training, measured by improved task scores and performance in apicoectomy. | Trainee confidence and satisfaction with preclinical training | Educational | Educational | Virtual simulation training (VSTG) showed significantly greater training enhancement compared to traditional methods (CG). VSTG resulted in higher confidence levels and greater satisfaction with the training compared to CG. - Supplementing traditional training with virtual simulation enhanced participants' theoretical and surgical performance in apicoectomy. |
| 37 | Liu et al.,2025/China | BMC Medical Education | Educational / Quasi-Experimental | Dental education and research | Educational | Orthodontics | 26 dental students | Online training: Virtual Learning Network Platform (VLNP), unlimited practice followed by formative and final tests Offline training: Virtual Simulation Training and Evaluation System (VSTES), haptic VR simulator, weekly sessions for 6 weeks Practical bracket bonding scored with 3D scanner before and after VR training; all procedures scored (cleaning, etching, bracket positioning, etc.) | VR | Virtual Simulation Training and Evaluation System (VSTES v.01, Unidraw Co. Ltd., Beijing, China); Virtual Learning Network Platform (VLNP v.01, Nanjing Medical University, Nanjing, China) | Improvement in total score of bracket bonding, with a 50% increase from pre-training to post-training scores. | Student feedback on system usability, preference for online/offline modules, skill plateau analysis | Educational | Operator-related | 1.Post-training bracket bonding scores increased by ~50% compared to baseline (P<0.05) 2. Significant improvements observed in etching, rinsing, bracket positioning, and total scores online and offline 3.Offline/haptic training preferred by majority for three-dimensional adjustment and real-time feedback 4. Most students found the VR environment realistic and useful for repeated, self-paced practice; noted limitations in haptic sensitivity and online module realism |
| 38 | Lucio et al.,2024/Spain | Healthcare technology letters | Educational / pre-experimental | Dental education and research | Educational | Oral Surgery | 33 dental students and dental practitioners (30 students & 3 dental practitioners) | Developed a VR educational tool (“IMPLANTIGRAPH”) featuring: 3D anatomical models of jaws Conventional labels and interactive knowledge maps (structured as directed acyclic graphs – DAGs) Tasks included memorization phases followed by quizzes on anatomical landmarks and relationships Quantitative data: quiz completion time, quiz accuracy (scores) Qualitative data: user satisfaction, system usability (SUS), workload (NASA-TLX), interviews | VR | Oculus Quest headset (Meta Platforms, Menlo Park, CA, USA) | Effectiveness of knowledge maps in VR for learning and teaching surgical anatomy (measured by quiz completion times and scores). | User satisfaction, preferences, perceived usability, and task workload (assessed through SUS, NASA-TLX, and other questionnaires). | Educational | Operator-related | Knowledge maps combined with 3D models help visualize hierarchical and topological anatomical relationships, aiding comprehension Students using VR with knowledge maps showed fewer incorrect answers on more complex tasks, though overall quiz score differences were inconclusive Completion times did not significantly differ between groups |
| 39 | Mahrous et al., 2021/USA | Journal of dental education | Educational / Quasi-Experimental | Dental education and research | Educational | Anatomy | 80 dental students | Eighty first-year dental students in a dental anatomy course visited four stations featuring different versions of a mandibular first molar: an extracted natural tooth, a 3D-printed model, a 3D virtual model, and a model shown via AR. After exploring each station, they completed an electronic survey. | AR | Merge AR/VR Headset (Merge Labs Inc., San Antonio, TX, USA); iPhone (Apple Inc., Cupertino, CA, USA) | The realism and fidelity of each model compared to a natural mandibular first molar. | Ease of use and overall user experience Educational value and student satisfaction with each modality | Educational | Educational | 1. Natural extracted teeth were rated by students as having the highest educational value. 2. 3D printed teeth were rated as the easiest to use. 3. The augmented reality (AR) model was rated as the most interesting by students. |
| 40 | Mansoory et al., 2022/Iran | BMC medical education | Educational / True Experimental | Dental education and research | Educational | Prosthodontics | 50 dental students | 50 dental students randomly divided into experimental (n = 25) and control (n = 25) groups. Students’ performance in both groups was assessed using tests. A questionnaire was used to assess the usability of VR technology and students’ satisfaction with it. | VR | Samsung Gear VR headset (Samsung Electronics Co., Ltd., Seoul, South Korea); custom Unity-based VR simulation. Samsung Company in cooperation with Oculus Company (owned by Facebook). | 1. The effect of using VR technology on teaching the procedures of neutral zone design and teeth arrangement in complete dentures. 2. The effect of VR technology on the academic performance of dental students compared to traditional teaching methods. | 1. The usability of VR technology in dental education from the perspective of faculty members. 2. The students' satisfaction with the use of VR technology in the learning process. | Operator-related | Educational | 1. The majority of faculty members (100%) confirmed the usability of VR technology in dental education. 2. The majority of students (76%) were highly satisfied with the use of VR technology in their learning process. |
| 41 | Marhuenda Ramos et al.,2024/Spain | BMC Oral Health | Educational/ True Experimental | Dental education and research | Educational | Oral Surgery | 40 Dental students and dental practitioners | 40 samples were divided into two groups: augmented reality (AR) and conventional free-hand technique (FT). - Preoperative and postoperative CBCT scans were used to assess accuracy. - Digital impressions and 3D planning software were used for planning. - AR group used Hololens2 for real-time guidance; FT group performed drilling manually. - Diamond bur was used for drilling. - Postoperative CBCT scans were aligned with preoperative plans to measure deviations. | AR | HoloLens2 mixed reality headset (Microsoft Corp., Redmond, WA, USA) Vuforia AR platform (PTC Inc.; integration in Unity) | Evaluate the reliability of an augmented reality drilling approach compared to a freehand drilling technique for auto transplantation of single-rooted teeth. | Measurements of coronal, apical, and angular deviations between planned and actual positions of autotransplanted teeth using both techniques. | Operator-related | Operator-related | 1.The augmented reality technique provided higher accuracy in the positioning of single-root autotransplanted teeth compared to the conventional free-hand technique. 2. Statistically significant differences were found in apical 3. The study rejected the null hypothesis, indicating that there are differences in accuracy between AR and FT techniques for auto transplantation. deviations between the AR and FT study groups, with AR showing better accuracy at the apical level. |
| 42 | McAlpin et al., 2022/USA | European journal of dental education | Educational / True Experimental | Dental education and research | Educational | Oral surgery | 58 dental students | 58 second- year students were randomly assigned to one of two groups, VR Sim or PM Sim. Both groups completed the same pre- post survey | VR | Oculus Quest VR headset (Meta Platforms, Inc., Menlo Park, CA, USA) | 1. Evaluating the performance of students in each group on a treatment test using their respective simulation methods. | Evaluating the transfer of skills from the simulation training to performing the procedure on a live patient. | Educational | Educational | 1. The plastic manikin simulation with a teaching assistant (PM Sim) resulted in higher learning gains compared to the virtual reality (VR) simulation (VR Sim). 2. The VR Sim group spent twice as long on average practicing compared to the PM Sim group before taking the treatment test. |
| 43 | Mladenovic et al., 2019/Serbia | Journal of Dental Education | Educational / True Experimental | Dental education and research | Educational | Oral Surgery | 41 dental students | Participants: 41 fourth- and fifth-year students (Control: 19, Experimental: 22) Intervention: Experimental group used a mobile AR simulator in a dental office (2h/week for 4 weeks) alongside standard instruction. | VR | Dental Simulator mobile app (Blantus Endodontic Centre, Campinas, Brazil) used with VR goggles and a plastic model; | Performance Metrics: Time taken to administer IANB and success rate of anaesthesia. Physiological Response: Heart rate monitored five minutes before and during anaesthesia administration to assess stress levels. | Knowledge and Skill Assessment: Post-clinical questionnaire evaluating self-perceived knowledge and skills after the procedure. | Educational | Educational | students in the experimental group who used the AR simulation spent significantly less time than the control group on the anaesthetic procedure, which suggests they recognized the point of injection for IANB in a faster and simpler way |
| 44 | Murbay et al., 2020/Hong Kong | European journal of dental education | Educational / True Experimental | Dental education and research | Educational | Conservative dentistry | 32 dental students | Thirty-two second-year undergraduate students were randomly divided into two groups: Group 1 (VR using Simodont dental trainer) and Group 2 (no VR exposure). Students performed a SISTA Classification—Site 1 assessment, evaluated by three assessors using both a manual approach and digital software in a single-blinded manner. | VR | Simodont Dental Trainer, V.4 software (Moog B.V., Nieuw-Vennep, Netherlands) | o evaluates the students' ability to perform Class I cavity preparations after training, comparing those who used the VR simulator to those who did not. | The study also aimed to determine if there was a significant difference between manual and digital evaluation methods in assessing the quality of the cavity preparations. | Educational | Educational | Enhanced Performance with VR Training: Students trained with the VR simulator demonstrated a higher rate of satisfactory cavity preparations (12 out of 16 students) compared to those without VR training (7 out of 16 students |
| 45 | Nauman et al.,2025/Pakistan | BMC Medical Education | Educational/Cross-sectional survey | Dental education and research | Educational | Conservative dentistry | 229 dental students | 229 dental students (2nd–4th year and house officers) Parallel preclinical curriculum: both VR simulation (Virteasy Dental haptic system) and manikin-based training Weekly group sessions for 7 weeks; same exercises in both formats (Class I–V preparations, endodontic access, isolation, etc.) v | VR | Virteasy Dental VR simulator (HRV Simulation, Montpellier, France). | To compare dental students' perceptions of VR simulation with traditional dental manikins as teaching tools at Ziauddin University. | To evaluate the impact of VR simulation on students' confidence and perceived preparedness for clinical practice. | Educational | Educational | 1. Both VR and manikin training were effective in improving learning outcomes for dental students. 2.Manikins were preferred for realistic clinical scenarios and confidence building, while VR was favoured for engagement and tactile learning. 3.The majority of students found both methods equally useful for reinforcing knowledge, suggesting that both are valuable tools in dental education. |
| 46 | Rehman et al.,2025/Malaysia | BMC Medical Education | Educational / pre-experimental | Dental education and research | Educational | General dentistry | 29 dental students | Virtual Dental Clinics (VDCs) developed using case scenarios and simulations based on clinical themes Students completed virtual clinical cases and quizzes over 2 weeks on an eLearning platform Student perceptions assessed via a 30-question Likert scale questionnaire (USE Questionnaire) evaluating usefulness, ease of use, ease of learning, and satisfaction | VR | Virtual Dental Clinics (developed with Articulate 360, Articulate Global Inc., New York, USA; hosted on IMU e-learning LMS, Kuala Lumpur, Malaysia). | Assess students' perceptions regarding the usefulness of Virtual Dental Clinics (VDCs) in simulated clinical training. | Evaluate VDCs as an enhancement to simulated learning, providing a holistic approach to case-based learning. | Patient-reported | Clinician-reported | Significant differences in perceived usefulness compared to ease of learning and satisfaction, indicating usefulness rated highest but room for improvement in learnability and satisfaction. No significant differences among ease of use, ease of learning, and satisfaction domains, suggesting comparable positive user experience there |
| 47 | Reymus et al., 2020/Germany | International endodontic journal | Educational / pre-experimental | Dental education and research | Educational | Endodontics | 42 dental students | Extracted human teeth were digitized using a CBCT scan and converted into STL files. The corresponding files were either 3D printed or imported into a VR software program. Subsequently, forty-two third-year undergraduate dental students in preclinical training was asked to respond to a questionnaire analysing their ability to detect all the anatomic features of the replica teeth and their comprehension of the underlying root canal anatomy. | VR | HTC VIVE headset (HTC, Taoyuan, Taiwan); VR visualization software VRED 2018 (Autodesk, San Rafael, CA, USA). HTC, Taoyuan, Taiwan) | to evaluate the feasibility and effectiveness of using virtual reality (VR) to teach root canal anatomy to dental students, compared to traditional methods like 2D radiographs and CBCT. | to assess student feedback and perceptions on the different teaching methods. | Educational | Educational | 1) CBCT and VR were both superior to 2D radiographs in helping students understand root canal anatomy. 2) Students preferred the VR method over CBCT for learning root canal anatomy, likely due to the interactive nature of the VR experience. 3) The majority of students had no problems using the VR system. |
| 48 | San Diego et al., 2022/UK | Dentistry journal | Educational/ True Experimental | Dental education and research | Educational | Conservative dentistry | 264 dental students | 264 The students in each study were randomly assigned to the phantom and haptics condition groups. | VR | hapTEL haptic dental simulator (King’s College London, London, UK; University of Reading, Reading, UK; Birmingham City University, Birmingham, UK). | The effectiveness of the haptic dental task trainer simulator compared to the traditional mannequin simulator in learning cavity preparation skills, as evaluated by comparing the students' performance between the two simulator conditions | whether there was a difference in performance between the two simulator conditions in removing artificial caries, based on specific marking criteria including total scores and ratings on 5 different criteria. | Patient-reported | Clinical | 1) There was no significant difference in overall performance between the haptic dental simulator group and the traditional phantom head simulator group in removing artificial caries, though the phantom head group performed slightly better on the more difficult task. 2) Students in the haptic simulator group were less likely to be perceived as "holding the instruments appropriately" compared to the phantom head group. |
| 49 | Schneider et al., 2025/Austria | Oral and Maxillofacial Surgery | Educational / pre-experimental | Dental education and research | Educational | Implant Dentistry | 40 dental students | 40 pre-clinical dental students (24 male, 16 female; mean age 23.5 years) Digitally designed 3D-printed Frasco training model with milled Saw bone insert. Four pilot drillings per student: two freehand (quadrants 21 and 23) and two AR-guided (quadrants 11 and 13) Drill depth standardized at 10 mm; model positioned in phantom head Accuracy assessed via D800 3D scanning and best-fit alignment to digital plan (distance and angulation deviations) | AR | Smartphone: Samsung Galaxy S22 (Samsung, South Korea) running custom AR app | Usefulness of smartphone-based AR for CAIS regarding angulation and positioning of dental implants. | Evaluation of drilling accuracy (parallelling post position). | Educational | Educational | 1)The study found no significant difference in distance between freehand and AR-guided implant placements. 2) Implant angulation was significantly more accurate with AR guidance compared to freehand placement. |
| 50 | Singh et al., 2024 / India | Journal of Pharmacy and Bioallied Sciences | Educational / True Experimental | Dental education and research | Educational | Conservative dentistry | 60 dental students | VR Group (n=30): VR dental simulation platform covering cavity preparation, crown placement, and periodontal procedures Control Group (n=30): Traditional hands-on practice with dental models under instructor supervision Clinical skills assessed via standardized checklist (0–100% | VR | VR stimulator (India) | Improvement in clinical skills | Increase in student confidence | Patient-reported | Clinical | The VR-based simulation training group showed a significant improvement in clinical skills, with a mean score increase from 65% to 85%, a 20.4% increase |
| 51 | Slaczka et al.,2024/USA | International Endodontic Journal | Educational / True Experimental | Dental education and research | Educational | Endodontics | 40 dental students | 40 dental students (from initial 46, after dropouts) randomly divided .Test group (n=22) trained using Simodont® haptic virtual reality simulator (HVRS) Control group (n=18) trained on artificial teeth (AT) mounted in phantom heads (PH) Pre- and post-training assessments done on AT mounted in PH, graded using a rubric scale (0–17) by blinded examiners | VR | Simodont dental trainer (Nissin dental products, Japan) | Evaluate the evolution in the performance of learners trained to prepare access cavities using Simodont compared to those trained using artificial teeth (AT) mounted on a phantom head. | Determine learner acceptability of the haptic virtual reality simulator (HVRS). | Educational | Educational | No statistically significant difference in improvement between Simodont and AT training groups (23% improvers in Simodont group vs. 39% in AT group; p=0.315) Number of pulpal floor perforations post-training was not significantly different (5 in Simodont group vs. 2 in AT group; p=0.427) Students found Simodont helpful for visualizing the pulp chamber anatomy and enjoyed the training |
| 52 | Sladina et al., 2023/ Latvia | European journal of dental education | Educational / pre-experimental | Dental education and research | Educational | Prosthodontics | 48 dental students | 48patient-specific virtual simulation tasks (one per student) | VR | Simodont dental trainer (Nissin dental products, Japan) | Student acceptance of patient-specific VR simulation training and satisfaction with workflow | Technical performance of evaluated system and impact on clinical skills | Educational | Educational | Patient-specific virtual simulation was well-received by students, enhancing engagement and interest in training tasks. Technical issues with Simodont Dental Trainer and realism concerns were noted, suggesting a need for improvements. |
| 53 | Song et al., 2024/USA | Journal of Prosthodontics | Educational/ True Experimental | Dental education and research | Educational | Prosthodontics | 31 dental students | 31 dental students – Four complete-arch optical scans per participant (two ARIOS, two IOS) of typodont in phantom head – Entry and exit surveys; NASA-TLX workload survey – Accuracy comparison via 3D cast RMSE against laboratory scanner reference | AR | AR headset: Epson Moverio BT-35E (Epson America Inc., Los Alamitos, CA, USA) Video capture device: StarTech USB 3.0 Video Capture Device (StarTech.com, Groveport, OH, USA) AR tracking software: Vuforia Augmented Reality SDK (PTC Inc., Boston, MA, USA) Custom platform software: Unity 2019.4.37f1 (Unity Technologies, San Francisco, CA, USA) | Comparison of scanning time and image count between ARIOS and IOS and Accuracy of digital casts derived from ARIOS and IOS | Participant preference for ARIOS or IOS. Cognitive workload as measured by NASA-TLX | Educational | Educational | 1.Participants showed a significant increase in preference for ARIOS over IOS, with 54.8% favouring ARIOS due to improved ergonomics, scanner tracking, and ease of orientation. 2.The accuracy of digital casts derived from ARIOS and IOS was comparable in terms of root mean square error (RMSE). 3.ARIOS required more scanning time and image counts compared to IOS, but was advantageous in ergonomics and scanner orientation. |
| 54 | Su Yin et al., 2021/Thailand | Journal of Biomedical Informatics | Educational / True Experimental | Dental education and research | Educational | Endodontics | 30 dental students | 30 dental students from Thammasat University School of Dentistry, Thailand, participated. They were divided into 3 groups: Group 1 (G1): Trained using the VR simulator without feedback. Group 2 (G2): Trained using the VR simulator with video-based formative feedback. Group 3 (G3): Trained using traditional laboratory methods without the VR simulator | VR | PHANTOM Omni haptic device (SensAble Technologies Inc., Wilmington, MA, USA) Software: Developed using OpenHaptics SDK 2.0 (3D Systems, Rock Hill, SC, USA) and Optimized Collision Detection (OPCODE, France) | The primary outcome measure was the average outcome score from pre- and post-training assessments by a panel of two expert raters, with scores ranging from 1 to 100 and 70 being the clinically acceptable passing score | The secondary outcome measures were the scores on the four axial walls (Mesial, Distal, Buccal, Lingual) and the pulp chamber floor. | Patient-reported | Clinical | 1. Skill training using the simulator with video-based formative feedback is more effective than training using the simulator without feedback. 2. Skill training using the simulator with video-based formative feedback is more effective than the traditional training approach. |
| 55 | Sytek et al., 2021/ USA | Journal of dental education | Educational / True Experimental | Dental education and research | Educational | Orthodontics | 20 dental students | 20 postgraduate dental students | VR | VR system (Oculus VR headset, Menlo Park, CA) | Objective performance accuracy of orthodontic residents using 2D, 3D, and virtual reality surgical simulation methods. | Residents’ attitudes, perceptions, and preferences toward 2D, 3D, and virtual reality simulation methods, and task completion time. | Operator-related | Educational | Simulation methods of increased fidelity (3D and VR) are appropriate alternatives to 2D conventional orthognathic surgical simulation methods when combined with traditional records. |
| 56 | Tamayo-Estebaranz et al., 2024/Spain | Journal of Personalized Medicine | Educational/ True Experimental | Dental education and research | Educational | Oral Surgery | 60 Dental students and dental practitioners | 60 osteotomy site preparations were assigned to two groups: AR device (n=30) and conventional free-hand method (n=30). Preoperative and postoperative CBCT scans were used. Intraoral scans and specialized software were used for virtual planning and segmentation. AR device was used for visualization in the AR group. Trephine bur was used for osteotomy preparations. | AR | – HoloLens 2 (Microsoft, USA) – Software: NemoScan® (Nemotec, Spain) – INNOAREA AR device (Valencia, Spain) also mentioned as visualization platform – Vuforia AR platform integration | Assess the accuracy of apical location using an AR device compared to a free-hand method. | Measurements of accuracy at the coronal, apical, and angular levels. | Educational | Educational | 1.The study found no statistically significant differences in accuracy between the augmented reality (AR) technique and the conventional free-hand method (FHM) for apical location in apicoectomy procedures. 2. Both the AR and FHM techniques were found to be equally accurate for apical location, confirming the null hypothesis. 3. The AR technique did not show statistically significant improvements in accuracy at the coronal, apical, and angular levels compared to the FHM technique. |
| 57 | Usta et al., 2025 / Turkey | International Endodontic Journal | Educational / True Experimental | Dental education and research | Educational | Endodontics | 40 dental students | 40 third-semester dental students total: Group 1 (VR Haptic Simulator group): 20 students Group 2 (Control group): 20 students. Initial screening: 55 volunteer students (40 met inclusion criteria | VR | VirTeaSy Dental simulator(VirTeaSy Dental© France) | Evaluate the impact of VR haptic simulators on manual dexterity, self-confidence, and stress levels of dental students during preclinical endodontic training. | Determine the most effective timing for introducing VR simulators into the curriculum, whether before or after training with artificial teeth | Educational | Educational | 1/ Group 1 demonstrated significantly higher manual dexterity scores and self-assessed proficiency compared to Group 2. 2. Stress levels were reduced in both groups after using the simulator, with Group 1 reporting lower anxiety levels during preclinical training with artificial teeth. 3. Self-confidence was higher in Group 1, indicating that using VR haptic simulators before training with artificial teeth enhances confidence in performing clinical skills. |
| 58 | Vincent et al., 2022/France | Journal of dental education | Educational / True Experimental | Dental education and research | Educational | Implant dentistry | 88 dental students | 88 First-year dental students were randomly assigned to two groups. Group 1 prepared an implant site for a left first mandibular molar on a haptic simulator with assistance, while Group 2 performed the same task without assistance. | VR | Virteasy haptic simulator (HRV Simulation, Changé, France) | 1) Determining if haptic simulator assistance can improve drilling ability in implantology 2) Determining if repeated training sessions can highlight a learning curve | The secondary outcomes were the various objective parameters measured by the simulator, including position, angulation, drilling depth, drilling time, total time, and presence of cortical bone perforation. | Educational | Educational | 1. Haptic simulator assistance can improve drilling ability in implantology. 2. Repetition of training sessions highlights a learning curve where students gradually acquire the necessary skills to perform the task without virtual aids. |
| 59 | Vincent et al., 2019/France | Journal of dental education | Educational / True Experimental | Dental education and research | Educational | Restorative Dentistry | 88 dental students | In early 2019, 88 first-year dental students in France were randomly assigned to two groups: Group 1 (n=45) used a haptic simulator (Virteasy) for cavity preparations, while Group 2 (n=43) performed conventional work on plastic analogue teeth (Kavo). | VR | Virteasy haptic simulator (HRV Simulation, Changé, France) | to evaluate the contribution of virtual reality to the conventional analogic training environment and show the complementarity of conventional techniques and virtual reality in the learning of dental students | the objective parameters measured by the simulator (tissue removal, drilling time) and the subjective parameters evaluated by the teachers (cavity shape, depth, floor regularity, iatrogenic milling). | Operator-related | Educational | 1. The use of a haptic simulator (virtual reality) for cavity preparation training led to similar results as the traditional training on plastic analogue teeth, with both groups showing improvement in their drilling skills over time. 2. The haptic simulator allowed for more objective assessment of student performance compared to the traditional plastic analogue teeth method. |
| 60 | Yaning Li et al., 2025 / China | Journal of Medical Internet Research | Educational / True Experimental | Dental education and research | Educational | Prosthodontics | 80 dental students | Participants stratified by gender and academic performance, then allocated to eight groups: Groups 1–3: exclusive training on Unidental VR simulator, Simodont VR simulator, or traditional phantom heads, respectively Groups 4–8: hybrid sequences combining VR simulators and phantom-head training (simulator-first and phantom-first sequences). Each participant performed veneer preparation on a maxillary central incisor. Evaluations used a 100-point rubric assessing marginal integrity, preparation depth, proximal contour, and surface smoothness. | VR | Unidental VR simulator (Unidraw, China; version V1.0) Simodont VR simulator (Nissin, Netherlands) | Comparison of the effectiveness of VR simulators (Unidental and Simodont) with traditional phantom heads for veneer tooth preparation training. | Evaluation of user perceptions of simulator realism, haptic feedback, and educational value; assessment of the impact of training sequence (simulator-first vs phantom-head-first) on skill acquisition. | Educational | Educational | 1) there were no statistically significant differences in preparation quality among groups using Unidental, Simodont, or traditional phantom heads for veneer tooth preparation training. 2) Significant differences were observed in user perceptions of realism and force feedback between Unidental and Simodont. |
| 61 | Yari et al.,2024/Iran | Journal of Dental Education | Educational / True Experimental | Dental education and research | Educational | Oral surgery | 40 dental students | Forty fourth-year dental students, randomly divided into control (conventional books) and study (AR Book) groups, 20 each. An AR Book was created from a textbook chapter on nerve block, with interactive 3D models and videos shown via a mobile app (Zappar). The control group used regular printed or digital books and class slides. Students studied 1.5 hours daily for a week and rated their focus during study. They took a written test, had their anaesthesia skills tested practically, and measured anaesthesia success and injection time. | AR | Zappar AR platform (software), created using 3D modelling software (Autodesk 3ds Max, Blender) | Improvement in theoretical knowledge and practical skills related to administering inferior alveolar nerve block | Concentration during study, success rate of anaesthesia, time efficiency | Educational | Educational | There was a significant difference in concentration levels, practical skill scores, and needed time for injection between the control and study groups. Success rate of local anaesthesia was similar between the two groups. Written exam scores were higher in the study group but not significantly so. |
| 62 | Zafar, Lai et al., 2020/Australia | International Journal of Paediatric Dentistry | Educational / pre-experimental | Dental education and research | Educational | Paediatric dentistry | 100 dental students | 100 dental students who completed training in pulpotomies and stainless-steel crowns using both the Simodont® VR simulator and conventional simulation methods completed a questionnaire assessing their experiences with both training methods | VR | Moog Simodont® Dental Trainer (Nissin Dental Products Europe BV, Netherlands) | the main outcome was to assess how students perceived the VR simulator (Simodont®) as an aid to their learning in paediatric dentistry training. | he studies evaluated how well the VR simulator helped students understand specific procedures like pulpotomies and stainless-steel crowns | Educational | Educational | 1) Simodont® can be a useful adjunct to conventional simulation training for paediatric dentistry procedures, helping to improve student confidence and understanding. 2) However, Simodont® should not completely replace conventional simulation training, as the majority of students prefer the traditional approach. |
| 63 | Zafar et al., 2021/Australia | European archives of paediatric dentistry | Educational / pre-experimental | Dental education and research | Educational | Paediatric dentistry | 71 dental students | 71 Participants completed questionnaires before and after interacting with the dental LA VR simulator. | VR | Oculus Quest (Oculus VR, Irvine, CA, USA) | The main focus was on evaluating how participants perceived the improvement in their local anaesthetic skills after using the VR simulator | The study assessed how engaging the VR simulator was compared to traditional training methods. | Educational | Educational | 1. The use of VR simulation can enhance student engagement and learning experience in paediatric dentistry local anaesthesia (LA) training. 2. VR simulation can be used as an additional tool for LA training, but it should not replace traditional phantom head training methods. 3. Over 50% of students felt the VR simulation added value and assisted their learning compared to traditional methods. |
| 64 | Zafar et al., 2020/Australia | European journal of dental education | Educational / pre-experimental | Dental education and research | Educational | Anatomy | 88 dental students | 88 students participated in a self-administered questionnaire prior to and after the use of AR. This was conducted during anatomy classes for second-year dentistry students. | AR | HoloLens (HoloHuman, 3D4 Medical Ltd., Ireland) | to evaluate the effectiveness of the HoloHuman augmented reality application in improving dental students' anatomy knowledge. | to compare the anatomy skills developed through HoloHuman versus traditional cadaver-based anatomy practical. | Educational | Educational | 1.Students had high expectations for the HoloHuman application prior to using it, with 82% excited about using it, 77.3% perceiving it as user-friendly, and 75% expecting it to assist their learning. 2. After using the HoloHuman application, students had more mixed perceptions, with only 36.5% finding it user-friendly, 36.5% feeling comfortable using it, and 50.6% feeling engaged in the learning activity. |
| 65 | Zhang et al., 2020/China | BMC medical education | Educational / True Experimental | Dental education and research | Educational | Implant dentistry | 80 dental students | 80 Students, randomized to either three experimental groups or one control group. The subjects undertook theoretical examinations to test their basic level of knowledge after training in similarly unified knowledge courses. Each student group then participated in an eight-hour operating training session The four groups included one control group and three experimental groups: (1) jaw simulation model as control, (2) virtual simulation, (3) virtual-jaw (V-J), (4) jaw-virtual (J-V). | VR | UniDental-MS01（Zhonghui Technology Institute, China) | The primary outcome was to examine the effect of VR goggles on pain perception during local anaesthesia administration, as measured by both self-reported pain | to examine the effect of VR goggles on pain perception during rubber dam placement, also measured by both self-reported and observed pain. | Educational | Educational | The use of VR goggles did not significantly reduce pain perception during the administration of local anaesthesia. 2. The use of VR goggles did significantly reduce pain perception during the placement of a rubber dam. 3. The participants all preferred the dental visits with the use of VR goggles. |
| 66 | Zhang et al., 2021/China | BMC medical education | Educational / True Experimental | Dental education and research | Educational | Periodontology | 60 dental students | Sixty stomatology students (sophomores and juniors) at Lanzhou University were randomly assigned to four groups (n = 15 each): traditional jaw model (control), virtual reality, virtual‐jaw, and jaw‐virtual. | VR | UniDental（Zhonghui Technology Institute, China) | The primary outcome of the study was the scores on the second theoretical assessment, which were significantly improved for the V-J and J-V groups compared to the first theoretical exam. | The secondary outcomes were the operation process scores and the supragingival scaling performance, which were both significantly higher for the V-J and J-V groups compared to the other groups. | Educational | Educational | 1. The combination of virtual reality (VR) and jaw model during periodontal preclinical training can increase students' grades and significantly improve their professional skills. 2. The jaw model should be applied prior to VR to optimize learning skills in basic periodontal education. |
| 67 | Zhou et al., 2021/China | Medicine (Baltimore) | Educational / True Experimental | Dental education and research | Educational | Implant dentistry | 30 dental practitioners | Thirty physicians were divided into two groups: Group A used a VR dental implant training system, and Group B received conventional theoretical training with clinical demonstration. After a 1-month training period, all physicians completed a questionnaire, and results were compared between groups. | VR | HTC vive helmet and its matching handle (HTC Co., Ltd., China) | The primary outcome was the subjective evaluation of the VR dental implant training system through a questionnaire survey on the fidelity and user-friendliness of the simulator, as well as the participants' sense of implant placement and mastery of the implantation process | The secondary outcome was the objective evaluation of the participants' performance in the VR training system compared to the control group, measured by the deviations in the implant's 3D position in the mesiodistal, buccal and tongue, depth, and angle dimensions. | Educational | Educational | 1. The VR dental implant training system resulted in significantly higher scores in the 5 dimensions of postoperative assessment compared to the control group. 2. The VR training group had significantly lower deviations in the 4 dimensions of mesiodistal, buccal and tongue, depth, and angle compared to the control group, indicating a higher operation success rate. |
| 68 | Ziane-Casenave et al., 2022/France | European journal of dental education | Educational / Quasi-Experimental | Dental education and research | Educational | Conservative dentistry | 56 dental students | Fifty- six volunteers divided into five groups (non- dentists, 1st/3rd/final- year dental students, recent graduates) had three attempts to prepare an occlusal amalgam cavity using the simulator. | VR | The VirTeaSy© haptic dental simulator (VirTeaSy, HRV Simulation, Changé, France) | To assess the capability of the VirTeaSy © haptic dental simulator to discriminate between users with different levels of prior experience, using a clinical simulation exercise based on the preparation of an occlusal amalgam cavity | The secondary outcome was to investigate the progression of performance after repetitions of the exercise. | Operator-related | Educational | 1. The VirTeaSy haptic simulator was not able to effectively discriminate between users with different levels of practical and clinical experience. 2. There were no significant differences in progression rates between the different groups using the simulator. |

**Table S3.** Study characteristics of included studies included in the network meta-analysis.

| **article** | **arm** | **Type of RCT** | **intervention/general treatment** | **intervention_gr_2** | **Type_of_patients** | **Age (range)** | **Male** | **Female** | **test** | **control** | **n_t** | **n_c** | **RoB** |
| --- | --- | --- | --- | --- | --- | --- | --- | --- | --- | --- | --- | --- | --- |
| Alshatrat et al., 2019 | 2 | Split-mouth | Scaling and root planing | Intermediate Invasive (Injection-Based / Restorative) | Adults |  | 22 | 28 | VR | Tell-Show-Do | 50,00 | 50,00 |  |
| Prakash et al., 2025 | 2 | Parallel | Restorative treatment | Intermediate Invasive (Injection-Based / Restorative) | Children | 3–13 | 53 | 53 | VR | Tell-Show-Do | 53,00 | 53,00 |  |
| Heena et al., 2024 | 2 | Parallel | Local anaesthsia | Minimally Invasive (Injection-Based) | Children | 4-10 |  |  | VR | TV | 30,00 | 30,00 |  |
| Aminabadi et al., 2022 | 2 | Parallel | Amalgam restoration | Intermediate Invasive (Injection-Based / Restorative) | Children | 4-6 | 13 | 17 | VR | Tell-Show-Do | 15,00 | 15,00 |  |
| Hamdy et al., 2024 | 2 | Parallel | Local anaesthsia | Minimally Invasive (Injection-Based) | Children | 4-6 | 15 | 15 | VR | Tell-Show-Do | 15,00 | 15,00 |  |
| Ran et al., 2021 | 2 | Parallel | Dental treatment | Intermediate Invasive (Injection-Based / Restorative) | Children | 4–8 | 35 | 25 | VR | Tell-Show-Do | 55,00 | 49,00 |  |
| Du et al., 2022 | 2 | Parallel | Local anaesthsia&teeth extraction | Surgical / Highly Invasive Procedures | Children | 4-9 | 68 | 60 | VR | Tell-Show-Do | 60,00 | 64,00 |  |
| Alshatrat et al., 2021 | 2 | Parallel | Dental treatment | Intermediate Invasive (Injection-Based / Restorative) | Children | 5-12 | 22 | 32 | VR | Tell-Show-Do | 14,00 | 17,00 |  |
| Ceylan et al., 2024 | 2 | Split-mouth | Scaling and root planing | Intermediate Invasive (Injection-Based / Restorative) | Adults |  | 31 | 29 | VR | Tell-Show-Do | 60,00 | 60,00 |  |
| Salama et al., 2024 | 2 | Parallel | Dental treatment | Intermediate Invasive (Injection-Based / Restorative) | Children | 5–7 | 20 | 20 | VR | Tell-Show-Do | 20,00 | 20,00 |  |
| Kasimoglu et al., 2024 | 3 | Parallel | Pulpotomy | Intermediate Invasive (Injection-Based / Restorative) | Children | 6-10 | 30 | 30 | VR | TV | 30,00 | 30,00 |  |
| Kasimoglu et al., 2024 | 3 | Parallel | Pulpotomy | Intermediate Invasive (Injection-Based / Restorative) | Children | 6-10 | 30 | 30 | VR | Tell-Show-Do | 30,00 | 30,00 |  |
| Felemban et al., 2021 | 2 | Parallel | Local anaesthsia | Minimally Invasive (Injection-Based) | Children | 6-12 | 29 | 21 | VR | TV | 25,00 | 25,00 |  |
| Bagher et al., 2023 | 2 | Parallel | Dental treatment | Intermediate Invasive (Injection-Based / Restorative) | Children | 6-14 | 18 | 18 | VR | TV | 18,00 | 18,00 |  |
| Bahrololoomi et al., 2024 | 2 | Split-mouth | Pulpotomy | Intermediate Invasive (Injection-Based / Restorative) | Children | 6-8 | 14 | 16 | VR | Tell-Show-Do | 30,00 | 30,00 |  |
| Aly et al., 2025 | 2 | Parallel | Dental treatment | Intermediate Invasive (Injection-Based / Restorative) | Children | 7-10 | 13 | 9 | VR | Tell-Show-Do | 16,00 | 16,00 |  |
| Almugaiti et al., 2021 | 2 | Split-mouth | Local anaesthsia | Minimally Invasive (Injection-Based) | Adults |  | 10 | 11 | VR | Tell-Show-Do | 21,00 | 21,00 |  |
| Kösem et al., 2024B | 2 | Parallel | Tooth extirpation | Intermediate Invasive (Injection-Based / Restorative) | Children | 7-10 | 25 | 35 | VR | Tell-Show-Do | 30,00 | 30,00 |  |
| Kösem et al., 2024A | 2 | Parallel | Tooth extraction | Surgical / Highly Invasive Procedures | Children | 7-10 | 25 | 35 | VR | Tell-Show-Do | 30,00 | 30,00 |  |
| Kösem et al., 2024B | 2 | Parallel | Tooth **e**xtirpation | Intermediate Invasive (Injection-Based / Restorative) | Children | 7-10 | 25 | 35 | VR | Tell-Show-Do | 30,00 | 30,00 |  |
| Kösem et al., 2024Ab | 2 | Parallel | Tooth extraction | Surgical / Highly Invasive Procedures | Children | 7-10 | 25 | 35 | VR | Tell-Show-Do | 30,00 | 30,00 |  |
| Buldur et al., 2020 | 2 | Cross-over | Dental treatment | Intermediate Invasive (Injection-Based / Restorative) | Children | 7-11 | 38 | 32 | VR | Tell-Show-Do | 36,00 | 34,00 |  |
| Ghobadi et al., 2024 | 2 | Cross-over | Implant placement | Surgical / Highly Invasive Procedures | Adults |  | 36 | 37 | VR | Tell-Show-Do | 37,00 | 36,00 |  |
| Gurbuz et al., 2024 | 2 | Parallel | Gingivectomy and gingivoplasty | Surgical / Highly Invasive Procedures | Adults |  | 17 | 41 | VR | Tell-Show-Do | 28,00 | 30,00 |  |
| Padminee et al., 2022 | 2 | Parallel | Local anaesthsia | Minimally Invasive (Injection-Based) | Children | 7-12 | 37 | 33 | VR | app | 35 | 35 |  |
| Alshatrat et al., 2021b | 2 | Parallel | Dental treatment | Intermediate Invasive (Injection-Based / Restorative) | Children |  | 22 | 32 | VR | Tell-Show-Do | 14,00 | 17,00 |  |
| Sorribes De Ramón et al., 2023 | 2 | Parallel | Extraction of 3 molar | Surgical / Highly Invasive Procedures | Adults |  | 85 | 99 | VR | Tell-Show-Do | 93,00 | 91,00 |  |
| Lahti et al. 2020 | 2 | Parallel | Before dental treatment | Intermediate Invasive (Injection-Based / Restorative) | Adults |  | 84 | 171 | VR | Tell-Show-Do | 129,00 | 126,00 |  |

**Table S4.** Risk of bias assessment for the educational studies with the Medical Education Research Study Quality Instrument.

|  | Authors | Study design | Sampling | Type of data | Validity evidence | Data analysis | Outcomes | Overall score | Methodological quality |
| --- | --- | --- | --- | --- | --- | --- | --- | --- | --- |
| 1 | Ali et al., 2024 | 2,5 | 1,5 | 3,0 | 1,0 | 3,0 | 1,5 | 12,5 | Moderate |
| 2 | Alsufyani et al., 2023 | 2,0 | 1,5 | 3,0 | 1,0 | 3,0 | 1,5 | 12,0 | Moderate |
| 3 | Azhari et al., 2024 | 2,0 | 1,5 | 3,0 | 1,0 | 3,0 | 1,5 | 12,0 | Moderate |
| 4 | de Boer et al., 2019 | 2,5 | 1,5 | 3,0 | 1,0 | 3,0 | 2,0 | 13,0 | High |
| 5 | Bui et al., 2024 | 2,0 | 1,5 | 3,0 | 1,0 | 3,0 | 1,5 | 12,0 | Moderate |
| 6 | Bandiaky et al.,2025 | 2,5 | 1,5 | 3,0 | 1,0 | 3,0 | 1,5 | 12,5 | Moderate |
| 7 | Cano-Martins et al., 2025 | 2,0 | 1,5 | 3,0 | 1,0 | 3,0 | 1,5 | 12,0 | Moderate |
| 8 | Cheng et al., 2025 | 2,0 | 1,0 | 3,0 | 1,0 | 3,0 | 1,0 | 11,0 | Moderate |
| 9 | Choi et al, 2025 | 2,0 | 1,0 | 3,0 | 1,0 | 3,0 | 1,0 | 11,0 | Moderate |
| 10 | Collaço et al., 2021 | 2,5 | 1,5 | 3,0 | 1,0 | 3,0 | 1,5 | 12,5 | Moderate |
| 11 | Daud et al., 2023 | 2,0 | 1,0 | 3,0 | 0,0 | 3,0 | 1,0 | 10,0 | Moderate |
| 12 | Daud et al.,2024 | 1,0 | 1,0 | 1,5 | 1,0 | 3,0 | 1,0 | 8,5 | Low |
| 13 | Diegritz et al.,2024 | 2,5 | 1,5 | 3,0 | 1,5 | 3,0 | 2,0 | 13,5 | High |
| 14 | Duan et al.,2024 | 2,5 | 1,5 | 3,0 | 1,5 | 3,0 | 1,5 | 13,0 | High |
| 15 | Dundar et al., 2025 | 2,0 | 1,0 | 3,0 | 1,0 | 3,0 | 1,0 | 11,0 | Moderate |
| 16 | Farag et al., 2022 | 2,0 | 1,0 | 3,0 | 1,0 | 3,0 | 1,0 | 11,0 | Moderate |
| 17 | Felszeghy et al., 2023 | 2,0 | 1,0 | 3,0 | 1,0 | 3,0 | 1,0 | 11,0 | Moderate |
| 18 | Fu et al., 2024 | 1,5 | 1,5 | 1,5 | 1,0 | 3,0 | 1,5 | 10,0 | Moderate |
| 19 | Fudalej et al., 2024 | 2,0 | 1,5 | 2,0 | 1,0 | 3,0 | 1,5 | 11,0 | Moderate |
| 20 | Grad et al., 2023 | 2,5 | 1,5 | 3,0 | 1,5 | 3,0 | 2,0 | 13,5 | High |
| 21 | Hamama et al.,2024 | 2,0 | 1,5 | 3,0 | 1,0 | 3,0 | 1,5 | 12,0 | Moderate |
| 22 | Haas-Stroede et al., 2025 | 2,5 | 1,5 | 3,0 | 1,5 | 3,0 | 2,0 | 13,5 | High |
| 23 | Hu et al., 2025 | 2,0 | 1,0 | 3,0 | 1,0 | 3,0 | 1,0 | 11,0 | Moderate |
| 24 | Huang et al., 2022 | 2,0 | 1,5 | 3,0 | 1,0 | 3,0 | 1,5 | 12,0 | Moderate |
| 25 | Huang et al., 2023 | 1,5 | 1,5 | 1,5 | 1,0 | 3,0 | 1,0 | 9,5 | Moderate |
| 26 | Im et al.,2025 | 2,5 | 1,5 | 3,0 | 1,5 | 3,0 | 1,5 | 13,0 | High |
| 27 | Iwanaga et al., 2021 | 1,5 | 1,0 | 1,5 | 1,0 | 1,5 | 1,0 | 7,5 | Low |
| 28 | Kaluschke et al., 2023 | 2,5 | 1,5 | 3,0 | 1,5 | 3,0 | 2,0 | 13,5 | High |
| 29 | Kihara et al., 2024 | 1,5 | 1,0 | 2,0 | 1,0 | 1,5 | 1,0 | 8,0 | Low |
| 30 | Kim-Berman et al., 2023 | 2,5 | 1,5 | 3,0 | 1,5 | 3,0 | 1,5 | 13,0 | High |
| 31 | Liebermann et al., 2024 | 2,5 | 2,0 | 3,0 | 1,5 | 3,0 | 1,5 | 13,5 | High |
| 32 | Liebermann et al., 2020 | 2,0 | 1,5 | 3,0 | 1,5 | 3,0 | 2,0 | 13,0 | High |
| 33 | Liebermann et al., 2024 | 2,0 | 1,5 | 3,0 | 1,5 | 3,0 | 1,5 | 12,5 | Moderate |
| 34 | Liebermann 2022 | 2,0 | 1,5 | 3,0 | 1,5 | 3,0 | 2,0 | 13,0 | High |
| 35 | Liebermann et al., 2023 | 2,0 | 1,5 | 3,0 | 1,5 | 3,0 | 2,0 | 13,0 | High |
| 36 | Liu et al.,2024 | 2,0 | 1,0 | 2,5 | 1,0 | 3,0 | 1,0 | 10,5 | Moderate |
| 37 | Liu et al.,2025 | 2,0 | 1,5 | 3,0 | 1,0 | 3,0 | 1,0 | 11,5 | Moderate |
| 38 | Lucio et al.,2024 | 1,5 | 1,5 | 1,5 | 1,0 | 1,5 | 1,0 | 8,0 | Low |
| 39 | Mahrous et al., 2021 | 2,0 | 1,5 | 3,0 | 1,5 | 3,0 | 1,5 | 12,5 | Moderate |
| 40 | Mansoory et al., 2022 | 2,0 | 1,0 | 2,0 | 1,0 | 3,0 | 1,0 | 10,0 | Moderate |
| 41 | McAlpin et al., 2022 | 2,0 | 1,0 | 2,0 | 1,0 | 3,0 | 1,0 | 10,0 | Moderate |
| 42 | Marhuenda Ramos et al.,2024 | 1,0 | 1,0 | 3,0 | 3,0 | 2,0 | 2,0 | 12,0 | Moderate |
| 43 | Mladenovic et al., 2019 | 2,0 | 1,0 | 2,0 | 1,0 | 3,0 | 1,0 | 10,0 | Moderate |
| 44 | Murbay et al., 2020 | 1,5 | 1,0 | 2,0 | 1,0 | 3,0 | 1,0 | 9,5 | Moderate |
| 45 | Nauman et al.,2025 | 1,5 | 1,0 | 1,5 | 0,5 | 1,5 | 1,0 | 7,0 | Low |
| 46 | Ougradar et al., 2019 | 1,5 | 1,5 | 2,0 | 1,0 | 2,0 | 1,0 | 9,0 | Moderate |
| 47 | Rehman et al.,2025 | 1,5 | 1,5 | 2,0 | 1,0 | 1,5 | 1,5 | 9,0 | Moderate |
| 48 | Reymus et al., 2020 | 1,5 | 1,5 | 2,0 | 1,0 | 3,0 | 2,0 | 11,0 | Moderate |
| 49 | San Diego et al., 2022 | 2,0 | 1,5 | 3,0 | 1,5 | 3,0 | 2,0 | 13,0 | High |
| 50 | Schneider et al., 2025 | 1,5 | 1,5 | 3,0 | 1,0 | 3,0 | 2,0 | 12,0 | Moderate |
| 51 | Singh et al., 2024 | 2,0 | 1,5 | 2,5 | 1,0 | 3,0 | 2,0 | 12,0 | Moderate |
| 52 | Slaczka et al.,2024 | 2,5 | 1,5 | 2,5 | 1,0 | 3,0 | 2,0 | 12,5 | Moderate |
| 53 | Sladina et al., 2023 | 2,0 | 1,5 | 2,5 | 1,0 | 3,0 | 2,0 | 12,0 | Moderate |
| 54 | Song et al., 2024 | 1,5 | 3,0 | 3,0 | 1,0 | 3,0 | 2,0 | 13,5 | High |
| 55 | Su Yin et al., 2021 | 2,0 | 1,5 | 2,0 | 1,0 | 3,0 | 2,0 | 11,5 | Moderate |
| 56 | Sytek et al., 2021 | 2,0 | 2,0 | 3,0 | 1,0 | 2,0 | 1,0 | 11,0 | Moderate |
| 57 | Tamayo-Estebaranz et al., 2024 | 1,5 | 2,0 | 3,0 | 1,0 | 2,0 | 1,5 | 11,0 | Moderate |
| 58 | Usta et al., 2025 | 2,0 | 1,5 | 2,0 | 1,0 | 3,0 | 1,5 | 11,0 | Moderate |
| 59 | Vincent et al., 2022 | 2,0 | 1,5 | 3,0 | 1,0 | 3,0 | 2,0 | 12,5 | Moderate |
| 60 | Vincent et al., 2019 | 2,0 | 1,5 | 2,5 | 1,0 | 3,0 | 2,0 | 12,0 | Moderate |
| 61 | Yaning Li et al., 2025 | 2,5 | 2,0 | 3,0 | 1,0 | 3,0 | 1,5 | 13,0 | High |
| 62 | Yari et al.,2024 | 2,0 | 1,5 | 2,5 | 1,0 | 3,0 | 2,0 | 12,0 | Moderate |
| 63 | Zafar, Lai et al., 2020 | 1,5 | 1,5 | 1,5 | 1,0 | 2,0 | 1,0 | 8,5 | Low |
| 64 | Zafar et al., 2021 | 1,5 | 1,5 | 1,5 | 1,0 | 2,0 | 2,0 | 9,5 | Moderate |
| 65 | Zafar et al., 2020 | 1,5 | 1,5 | 1,0 | 1,0 | 2,0 | 1,0 | 8,0 | Low |
| 66 | Zhang et al., 2020 | 2,0 | 2,5 | 3,0 | 1,5 | 3,0 | 2,0 | 14,0 | High |
| 67 | Zhang et al., 2021 | 2,0 | 1,5 | 2,5 | 1,0 | 3,0 | 2,0 | 12,0 | Moderate |
| 68 | Zhou et al., 2021 | 2,0 | 2,0 | 3,0 | 1,5 | 3,0 | 1,5 | 13,0 | High |
| 69 | Ziane-Casenave et al., 2022 | 1,5 | 1,5 | 2,5 | 1,5 | 3,0 | 2,0 | 12,0 | Moderate |

**Table S5.** Risk of bias assessment for quasi-experimental studies the Joanna Briggs Institute Critical Appraisal tool.

| **JBI critical appraisal checklist for quasi-experimental studies** | | | | | | | | | | |
| --- | --- | --- | --- | --- | --- | --- | --- | --- | --- | --- |
| **Reference** | **Question 1**  (Yes; No; Unclear; Not applicable) | **Question 2** (Yes; No; Unclear; Not applicable) | **Question 3** (Yes; No; Unclear; Not applicable) | **Question 4** (Yes; No; Unclear; Not applicable) | **Question 5** (Yes; No; Unclear; Not applicable) | **Question 6** (Yes; No; Unclear; Not applicable) | **Question 7** (Yes; No; Unclear; Not applicable) | **Question 8** (Yes; No; Unclear; Not applicable) | **Question 9** (Yes; No; Unclear; Not applicable) | **Overall appraisal** (Include; Exclude; Seek further info) |
| Martinez-Bernal et al., 2024 | Yes | No | Yes | Yes | Yes | No | Yes | Yes | Unclear | Include |

**Table S6.** Risk of bias assessment for cross-sectional studies the Joanna Briggs Institute Critical Appraisal tool.

| **JBI critical appraisal checklist for cross-sectional studies** | | | | | | | | | |
| --- | --- | --- | --- | --- | --- | --- | --- | --- | --- |
| **Reference** | **Question 1**  (Yes; No; Unclear; Not applicable) | **Question 2** (Yes; No; Unclear; Not applicable) | **Question 3** (Yes; No; Unclear; Not applicable) | **Question 4** (Yes; No; Unclear; Not applicable) | **Question 5** (Yes; No; Unclear; Not applicable) | **Question 6** (Yes; No; Unclear; Not applicable) | **Question 7** (Yes; No; Unclear; Not applicable) | **Question 8** (Yes; No; Unclear; Not applicable) | **Overall Risk of Bias** (Low; Moderate; High) |
| Ougradar et al., 2019 | Unclear | Yes | Unclear | No | No | No | Unclear | Yes | Moderate |

**Table S7.** Comparative table between current updated systematic review with pairwise meta-analysis and scoping reviews.

| Parameters | Current updated systematic review with pairwise meta-analysis | Sadilina S, Strauss FJ, Jung RE, Joda T, Balmer M. Use of optical see-through head-mounted display (OST-HMD) in dentistry: a scoping review. Int J Comput Dent. 2025 Jun 27;28(2):101-116. doi: 10.3290/j.ijcd.b5394865 | Sadilina S, Unkovskiy A, Jung R, Balmer M, Joda T. Augmented and mixed reality in implant dentistry: a scoping review exploring clinician- and patient-reported outcomes. Int J Comput Dent. 2026 Mar 10;0(0):0. doi: 10.3290/j.ijcd.b6952432 |
| --- | --- | --- | --- |
| Review type | Systematic | Non-systematic, scoping | Non-systematic, scoping |
| Following guideline | PRISMA 2020 | PRISMA-ScR | PRISMA-ScR |
| Objective | This updated systematic review aimed to synthesize and evaluate the available evidence on the use of augmented reality, mixed reality (MR), and virtual reality (VR) technologies in dental medicine and education. Both qualitative synthesis and quantitative analysis through pairwise meta-analysis, where applicable, were performed to assess their effectiveness and potential applications in clinical and educational settings. | The aim of the present scoping review was to identify the scientific evidence related to the utilization of optical see-through head-mounted display (OST-HMD) in dentistry, and to determine future research needs. | This scoping review aims to explore current trends in the scientific literature on clinician- and patient reported outcomes, as well as the feasibility of augmented reality (AR) and mixed reality (MR) technologies in implant-related dentistry and to assess future perspectives. |
| Investigated field | Dental medicine, including endodontics, cariology, orthodontics, periodontics, prosthodontics, pediatric dentistry, oral surgery, dental implantology, as well as oral pathology, oral radiology, dental public health, and oral medicine; and dental education, including undergraduate and postgraduate dental students; dental professionals. | Dentistry | Implant dentistry |
| Technologies evaluated | Augmented, mixed and virtual reality | Only optical see-through head-mounted display, as a particular device of augmented and mixed | Augmented and mixed reality |
| Data analysis and synthesis | Narrative synthesis, pairwise meta-analysis | Descriptive | Descriptive |
| Framework to formulate research question | modified PICOs framework | PCC framework | PCC framework |
| Meta-analysis | Yes | No | No |
| Risk of bias assessment | Yes | No | No |
| Quality of evidence | Yes | No | No |
| Number of included studies | 110 | 9 | 8 |
